# Potent and selective α-glucosidase inhibition by coumarin–triazole conjugates: design, *in vivo* evaluation, and computational insights

**DOI:** 10.1039/d6ra01115b

**Published:** 2026-06-01

**Authors:** Mahdis Sadeghi Moghadam, Bahareh Bayati, Fariba Peytam, Maryam Norouzbahari, Hayrettin Ozan Gülcan, Somayeh Mojtabavi, Fahimeh Ghasemi, Seyed Esmaeil Sadat-Ebrahimi, Maliheh Barazandeh Tehrani, Vahid Sheibani, Loghman Firoozpour, Alireza Foroumadi

**Affiliations:** a Department of Medicinal Chemistry, Faculty of Pharmacy, Tehran University of Medical Sciences Tehran Iran firoozpour@gmail.com aforoumadi@yahoo.com; b Drug Design and Development Research Center, The Institute of Pharmaceutical Sciences (TIPS), Tehran University of Medical Sciences Tehran Iran; c Faculty of Pharmacy, Final International University Catalkoy, Kyrenia *via* Mersin 10 Turkey; d Faculty of Pharmacy, Eastern Mediterranean University Famagusta, TRNC, *via* Mersin 10 Turkey; e Department of Pharmaceutical Biotechnology, Faculty of Pharmacy, Tehran University of Medical Sciences Tehran Iran; f Department of Medical Biotechnology, School of Advanced Technologies in Medicine, Tehran University of Medical Sciences Tehran Iran; g Neuroscience Research Center, Institute of Neuropharmacology, Kerman University of Medical Sciences Kerman Iran

## Abstract

Diabetes mellitus is a worldwide health problem, and high blood sugar is one of its hallmarks. Although α-glucosidase inhibitors such as acarbose are crucial for controlling postprandial blood glucose levels, their use is usually limited by digestive adverse effects. As a part of extensive efforts to identify potential anti-diabetic agents, a series of coumarin–triazole conjugates was designed and synthesized. They were evaluated for through *in vitro* inhibitory assays against α-glucosidase, achieving IC_50_ values ranging from 1.0 to 223 µM, significantly superior to that of acarbose (IC_50_ = 750 µM). Among them, compound 12q (bearing 3-CN) emerged as the most potent derivative (IC_50_ = 1.0 µM) and demonstrated selective inhibition of α-glucosidase over α-amylase. Kinetic studies confirmed 12q as a competitive inhibitor (*K*_i_ = 1000 nM), which allowed for its *in silico* evaluation in the active site. Molecular docking and dynamics simulations revealed the compounds' stable binding in the active site by interactions with critical catalytic residues such as Glu276 and Asp214. Fluorescence and circular dichroism studies supported high-affinity binding without major conformational changes in α-glucosidase. *In vivo* evaluation in a diabetic mouse model confirmed the significant antihyperglycemic efficacy of compound 12q, which outperformed acarbose in reducing fasting blood glucose and improving glucose tolerance. Collectively, these findings highlight this series of coumarin–triazole hybrids, particularly compound 12q, as promising candidates for the further structural development of safe and potent α-glucosidase inhibitors for diabetes management.

## Abstract 2

Compound 12q emerged as the most potent inhibitor (IC_50_ = 1.0 µM) and showed high selectivity toward α-glucosidase over α-amylase. The *in silico* studies further confirmed the activity. *In vivo* studies demonstrated that 12q outperformed acarbose.

## Graphical abstract

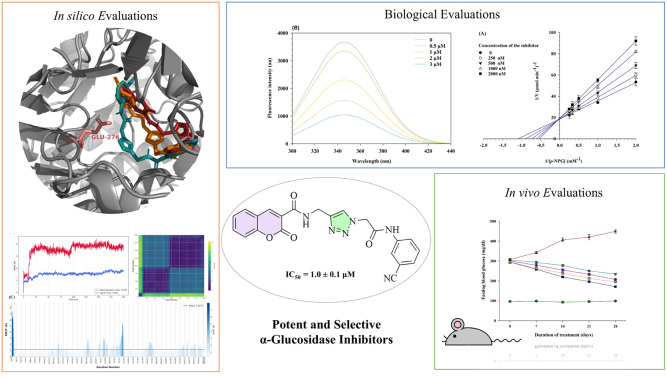

## 1 Introduction

According to the latest International Diabetes Federation (IDF) report, approximately 590 million adults between the ages of 20 to 79 years are living with diabetes mellitus. This number is expected to substantially rise to almost 853 million by 2050.^1^ This remarkable increase might be associated with risk factors such as urbanization, aging populations, decreasing physical activity, and rising obesity rates. Diabetes mellitus is characterized by chronic hyperglycemia resulting from defects in insulin secretion, insulin action, or both. Prolonged elevated blood glucose levels may cause damage to various organs, leading the development of disabling and life-threatening health complications, particularly microvascular (retinopathy, nephropathy, and neuropathy) and macrovascular complications, that significantly elevate the risk of developing cardiovascular diseases.^2^ Chronic hyperglycemia induces vascular damage through mechanisms such as oxidative stress, inflammation, and endothelial dysfunction, ultimately leading to impaired tissue and organ function.^3^

These complications remarkably increase the morbidity and mortality in individuals with diabetes and represent a major global health burden. One effective strategy for managing diabetes is controlling the postprandial hyperglycemia. α-Glucosidase is a hydrolase enzyme located at the brush border of the intestinal epithelium, where it catalyzes the cleavage of α (1 → 4) glycosidic bonds in starch and disaccharides, producing absorbable α-glucose from the non-reducing end of oligosaccharides. If the turnover rate of glucose produced by this enzyme is uncontrolled, pathological conditions such as type 2 diabetes and hyperglycemia can emerge.^4^

α-Glucosidase inhibitors (AGIs) are a class of oral anti-diabetic drugs that exert their effect by inhibiting this enzyme at the active or allosteric sites, thereby delaying carbohydrate digestion and glucose absorption in the intestine. This inhibition can retard the rise in postprandial blood glucose levels.^5^ Clinically approved AGIs, such as acarbose, miglitol, and voglibose, have been widely used and are particularly effective in patients with postprandial hyperglycemia.^6^ Among these approved AGIs, acarbose is the most common and effective drug, however, its clinical use is limited by several adverse gastrointestinal effects, most notably diarrhea, abdominal pain, bloating, and excessive gas. Consequently, extensive research efforts over the past decade have focused on the identification and development of novel AGIs with enhanced potency, improved safety, and promoted pharmacokinetic profiles as alternatives to acarbose. In this context, a wide variety of heterocyclic compounds have been introduced and evaluated as promising AGIs.^7–18^

**Fig. 1:**
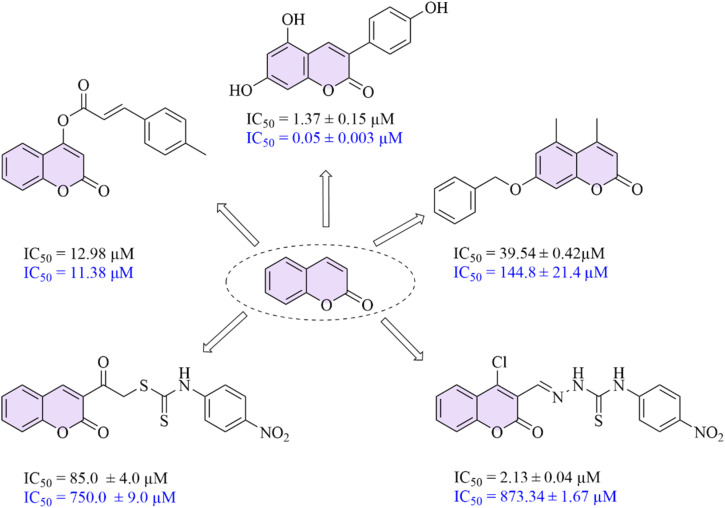
Coumarin incorporating scaffolds presenting *anti*-α-glucosidase activities (The IC_50_ values are written in black for inhibitors and blue for acarbose).

Coumarins, also known as 1,2-benzopyrones, belong to a class of phytochemicals widely found in natural sources. Following their discovery and initial isolation from *Coumarouna odorata*, numerous biological activities have been attributed to coumarin derivatives, leading to the development of a broad range of synthetic coumarin-based compounds. To date, the coumarin scaffold has been extensively employed in the design of biologically active compounds, including anticancer,^19^*anti*-carbonic anhydrase,^20^ antibacterial,^21^ antifungal,^22^ antiviral,^23^ anti-inflammatory,^24^ anti-Alzheimer,^25^ antioxidant,^26^ anticonvulsant,^27^ and anticoagulant^28^ agents. Furthermore, several coumarin-incorporating scaffolds have demonstrated significant α-glucosidase inhibitory activity, thereby exhibiting noticeable antidiabetic effects^29–33^ (Fig. 1).

**Fig. 2:**
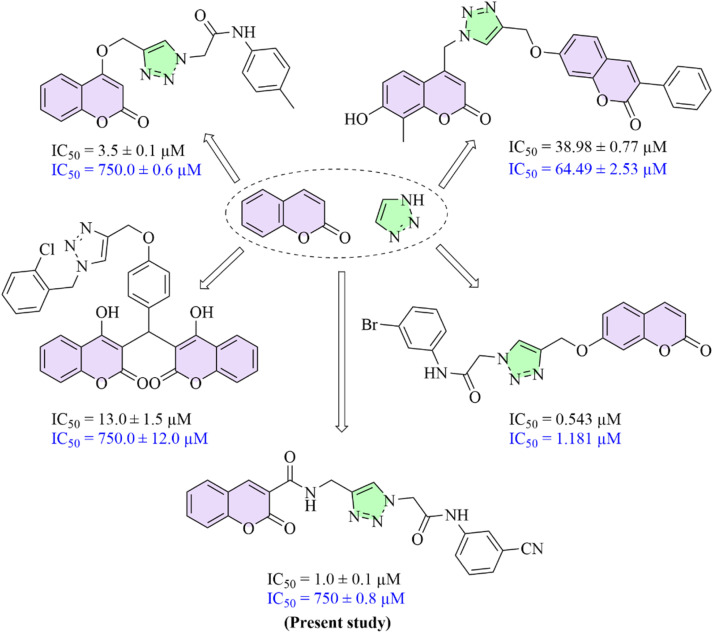
Compounds designed by hybridization of 1,2,3-triazole and coumarin rings as α-glucosidase inhibitors (The IC_50_ values are written in black for inhibitors and blue for acarbose).

1,2,3-Triazole ring has emerged as a valuable pharmacophore in chemical biology and pharmaceutical research. Their ability to form critical protein–ligand interactions, including hydrogen bonding and π-related interactions, along with their straightforward and high-yield synthesis *via* click chemistry, has established 1,2,3-triazole as a pivotal scaffold for hybridization with other backbones, leading to the development of further biologically active compounds with promising potential.^34^ The hybridization strategy is a key approach in modern drug discovery, involving the integration of multiple pharmacophores into a single scaffold to overcome the current limitations of individual structures and improve biological potency and selectivity.^35^ Considering both the advantages of molecular hybridization and the distinctive characteristics of the 1,2,3-triazole moiety, this scaffold has been extensively used in hybridization strategy to afford various biologically active compounds.^36,37^ In this context, the incorporation of substituted coumarin with 1,2,3-triazoles has led to the development of promising AGIs^38–41^ (Fig. 2).

Given our ongoing efforts toward design and synthesis of potential AGIs,^42–49^ an efficient synthetic route was applied to prepare a series of coumarin integrating 1,2,3-triazole and substituted *N*-arylacetamides 12. Although coumarin-triazole hybrids have been already investigated for diverse biological activities,^50^ our primary focus in this paper was evaluating their anti-diabetic properties. Therefore, comprehensive biological evaluations were conducted, including *in vitro* enzymatic inhibition assays and *in vivo* anti-diabetic studies in a mouse model. Additionally, spectroscopic analyses, such as circular dichroism and fluorescence quenching experiments, along with computational investigations, including homology modeling, molecular docking, and molecular dynamics simulations, were performed to validate the efficacy of the designed compounds.

## 2 Results and discussion

### 2.1 Chemistry

According to the literature, several classic routes have been reported for the preparation of the coumarin skeleton, including the Perkin reaction, Pechmann condensation, Knoevenagel condensation, and Claisen reaction.^51^ Depending on the starting materials, each of these approaches can lead to numerous substituted coumarin derivatives. In present study, an efficient synthetic route using salicylaldehyde 1 and diethylmalonate 2 was conducted to study to afford the desirable coumarin conjugated with 1,2,3-triazol and substituted *N*-arylacetamides 12.

**Scheme 1:**
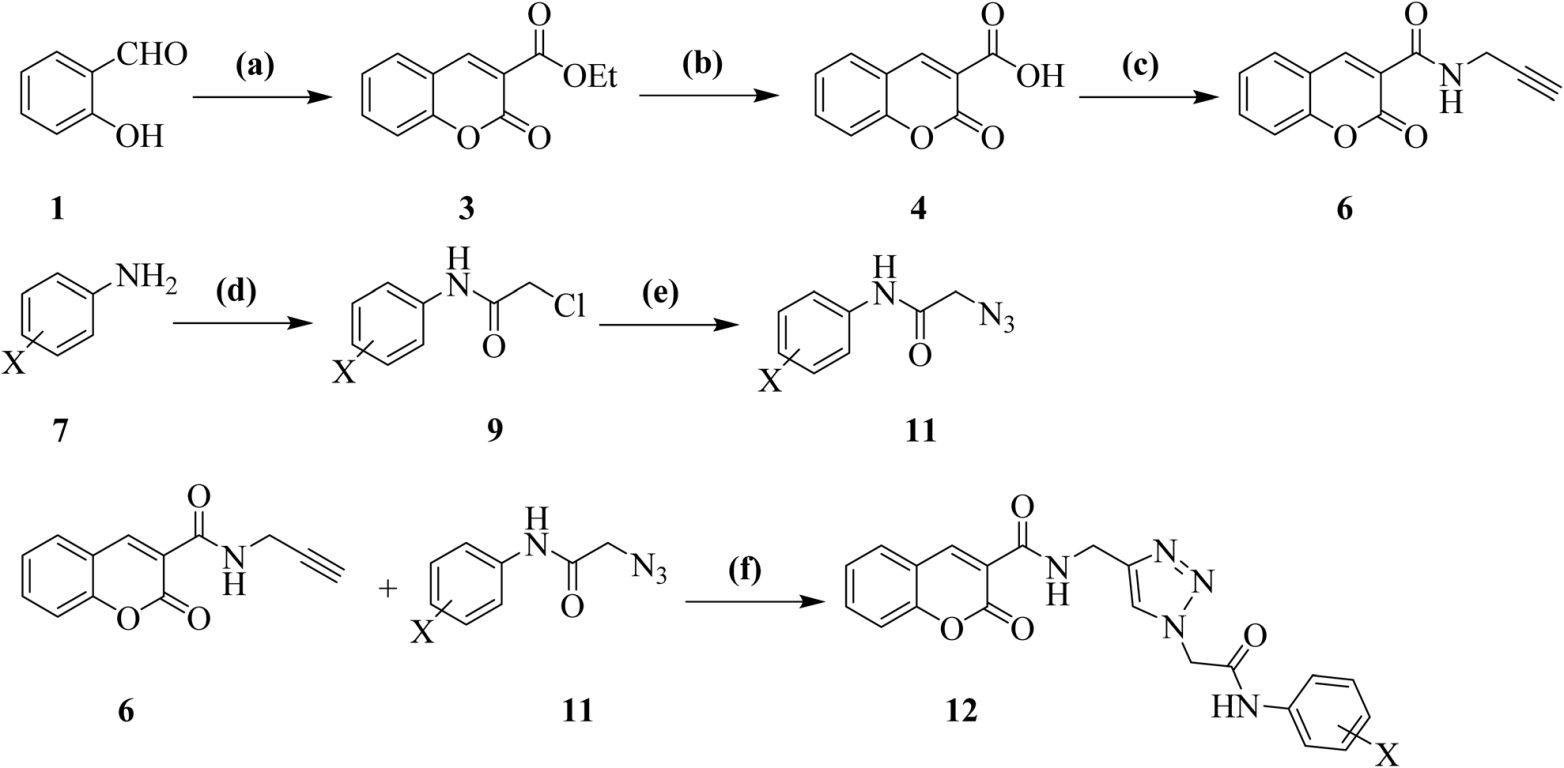
Reagents and conditions: (a) diethyl malonate 2, piperidine, in absolute EtOH, reflux, 4 h; (b) NaOH (10% aqueous solution), EtOH, reflux, 2 h; (c) propargylamine 2, TBTU, DIPEA, DMF, overnight; (d) chloroacetyl chloride 8, TEA, acetone, r.t., overnight; (e) sodium azide 10, DMF, r.t., overnight; (f) CuSO_4_·5H_2_O, sodium ascorbate, DMF, r.t., overnight.

As outlined in Scheme 1, these adducts were subjected to a Knoevenagel condensation, followed by an intramolecular cyclization, to obtain ethyl 2-oxo-2*H*-chromene-3-carboxylate 3. Subsequent alkaline hydrolysis of the ester functionality using aqueous NaOH solution yielded 2-oxo-2*H*-chromene-3-carboxylic acid 4. This adduct then went through an amidation reaction with propargylamine 5 to produce 2-oxo-*N*-(prop-2-yn-1-yl)-2*H*-chromene-3-carboxamide 6, having an acetylene moiety, which is an appropriate moiety for click reaction.

In a parallel synthetic pathway, various aniline derivatives 7 were reacted with 2-chloroacetyl chloride 8 to give corresponding substituted 2-chloro-*N*-arylacetamides 9. These intermediates subsequently underwent bimolecular nucleophilic substitution (S_N_2) with sodium azide (NaN_3_) 10 to afford substituted 2-azido-*N*-arylacetamides 11. The resulting azides were then subjected to Cu(i)-catalyzed azide–alkyne cycloaddition (CuAAC) with previously prepared compound 6 in the presence of CuSO_4_·5H_2_O and sodium ascorbate, synthesizing the substituted coumarins 12 in great to excellent yields.

**Fig. 3:**
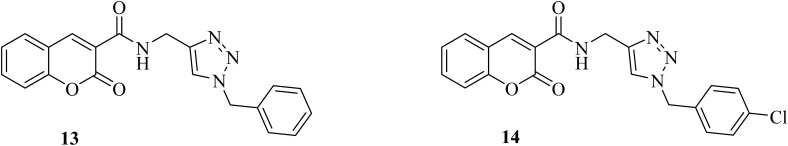
The structure of compounds 13 and 14.

For further evaluations to highlight the role of the amide functionality linked to the terminal phenyl ring on the α-glucosidase inhibitory activity, benzyl chloride and 4-chlorobenzyl chloride reacted with NaN_3_ to synthesize the corresponding benzyl azides, which were subsequently subjected to the same click reaction with compound 6, affording compounds 13 and 14, respectively (Fig. 3).

The structures of the isolated compounds 12, 13, and 14 were completely deduced based on the basis of their IR, ^1^H and ^13^C NMR spectroscopy, as well as high-resolution mass spectrometry and elemental analysis. Partial assignments of these resonances are given in the Experimental part.

### 2.2 α-Glucosidase inhibitory activity

A series of coumarin-1,2,3-triazole hybrids bearing *N*-arylacetamides (12a–12r) was designed and synthesized to evaluate for their *in vitro* inhibitory activity against α-glucosidase (from *Saccharomyces cerevisiae*) and to compare them with the standard drugs, acarbose and miglitol. In this study, α glucosidase inhibition was assessed using yeast α glucosidase, a widely used and accessible *in vitro* model for primary screening. Although the yeast enzyme originates from a different species, structural and mechanistic studies have revealed that yeast and mammalian α glucosidases share highly conserved catalytic (β/α)_8_ barrel domains, similar active site architectures, and identical catalytic residues. These enzymes also share comparable substrate sensitivities toward α-1,4- and α-1,6-linked glucosidic bonds. Therefore, yeast α-glucosidase is widely accepted as a reliable, cost effective, and well validated model for initial inhibitor screening. Nevertheless, the yeast enzyme does not fully replicate the physiological properties of mammalian intestinal α-glucosidase. Therefore, the inhibitory activity reported here must be interpreted as an initial *in vitro* evaluation of potential efficacy.

All tested compounds 12 demonstrated significantly superior potency (IC_50_ = 1.0 to 223 µM) to that of acarbose (IC_50_ = 750 µM), confirming the success of our molecular design strategy (Table 1). To investigate the structure–activity relationship (SAR) in this work, the terminal phenyl ring was functionalized with various substituents (X) possessing diverse electronic and steric properties. The structure and observed activity correlations (SAR analysis) were explained as follows:

**Table 1:** Substrate scope and *in vitro* α-glucosidase inhibitory activity of targeted compounds 12

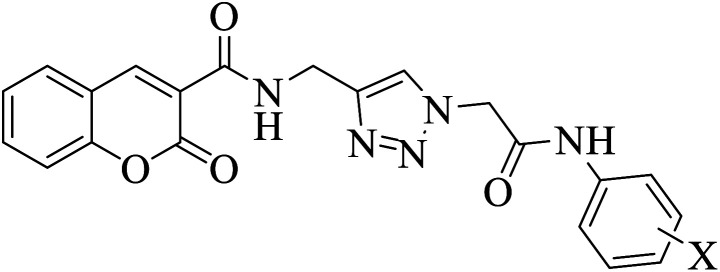					
Compound	X	IC_50_ (µM)^a^	Compound	X	IC_50_ (µM)^a^
12a	H	51.3 ± 1.2	12j	4-F	3.4 ± 0.1
12b	2-CH_3_	48.2 ± 1.0	12k	2-Cl	13.1 ± 1.0
12c	3-CH_3_	42.4 ± 0.7	12l	3-Cl	53.2 ± 1.4
12d	4-CH_3_	31.7 ± 1.3	12m	4-Cl	5.4 ± 0.1
12e	2-OCH_3_	28.8 ± 1.5	12n	4-Br	24.2 ± 1.5
12f	3-OCH_3_	40.8 ± 1.8	12o	4-CF_3_	30.2 ± 1.7
12g	4-OCH_3_	21.6 ± 0.6	12p	2-CN	83 ± 0.9
12h	3,4,5-OCH_3_	145.2 ± 1.6	12q	3-CN	1.0 ± 0.1
12i	2-F	6.9 ± 0.3	12r	4-CN	223 ± 1.6
**Acarbose**	—	750.0 ± 0.8	**Miglitol**	—	1200.0 ± 4.1

a Values are expressed as mean ± SD. All experiments were performed in triplicate as independent experiments.

The unsubstituted parent compound 12a (X = H, IC_50_ = 51.3 µM) exhibited moderate inhibitory activity. Introduction of electron-donating groups (EDGs) significantly affected this potency. The methoxy group at the *para* position (12g, 4-OCH_3_, IC_50_ = 21.6 µM) was more favorable than at the *ortho* (12e, 2-OCH_3_, IC_50_ = 28.8 µM) or *meta* (12f, 3-OCH_3_, IC_50_ = 40.8 µM) positions. The methyl group, a weak EDG, displayed a similar trend, with the *para*-substituted derivative 12d (4-CH_3_, IC_50_ = 31.7 µM) being the most active among its isomers. The significant negative impact of excessive steric bulk was obviously demonstrated by compound 12h (X = 3,4,5-OCH_3_, IC_50_ = 145.2 µM). Despite containing three methoxy groups, the noticeable steric hindrance due to the tri-substitution pattern severely reduced the binding affinity, leading to its lower activity than the parent compounds 12a, 12d, or 12f. These results revealed a sterically constrained binding pocket for the phenylacetamide moiety with the enzyme's active site.

To deepen the SAR analysis, further studies focused on the role of electron-withdrawing groups (EWGs) on α-glucosidase inhibitory potency. For instance, a comparative analysis of halogens revealed a subtle relationship between size and electronegativity, as compound 12j (X = 4-F, IC_50_ = 3.4 µM) was more potent than compounds 12m and 12n, bearing 4-Cl and 4-Br, respectively. The decreasing potency with increasing atomic radius suggested a limited size tolerance in the *para*-position of the terminal phenyl ring. It was further supported with the observed inhibitory activity of compound 12o (IC_50_ = 30.2 µM), bearing a bulkier trifluoromethyl group. Moreover, similar to the SAR trend in the previous category, the *para* position for these EWGs was more favorable than *meta* or *ortho* positions (as observed for compounds 12i, 12k, and 12l).

The most notable results were observed for the cyano (CN) group, which exhibited an exceptional position-dependent activity. Its presence at the *meta* position (compound 12q, 3-CN) resulted in the most potent inhibitor of the entire series (IC_50_ = 1.0 µM). The strong electron-withdrawing nature and linear geometry of this group in the *meta* position appeared to be optimal for interacting with a specific sub-pocket in the enzyme's active site. However, the presence of this group at the *para* position (compound 12r, 4-CN) led to the weakest inhibitory activity (IC_50_ = 223 µM). This dramatic decrease in potency due to the positional isomerism highlighted the substantial sensitivity of the enzyme's binding site to the three-dimensional orientation of functional groups. The *ortho*-CN analogue (compound 12p, 2-CN, IC_50_ = 83 µM) showed reduced activity as well, further emphasizing the suboptimal nature of *ortho* placement in this group.

Further SAR analysis focused on the critical role of amide functionality linked to the terminal phenyl ring. To this aim, compounds 13 and 14 were synthesized and assessed to compare their potencies with their corresponding derivatives from the first series (compounds 12a and 12m, respectively). The IC_50_ values were 62.5 ± 0.6 µM for compounds 13 and 34.2 ± 1.8 µM for compounds 14. As it will be discussed in the molecular docking study section, this moiety formed several substantial interactions, particularly H-bonds with Glu276 and Asp408, which makes it substantial for the α-glucosidase inhibitory effect.

Collectively, our detailed SAR analysis revealed that the presence of small, electronegative atoms at the *para* position of the terminal phenyl ring (such as compounds 12j and 12m) enhanced the α-glucosidase inhibitory potencies of our desired compounds. Moreover, the *meta*-CN moiety displayed exceptional potency (compounds 12p–12r), an effect that will be further clarified in the subsequent molecular docking studies.

### 2.3 *In silico* studies

#### 2.3.1 Homology modeling

The enzyme employed in the *in vitro* assays was α-glucosidase from *S. cerevisiae*. Since a high-resolution crystal structure for this specific enzyme isn't available, a homology model was constructed based on the structure of iso-maltase of *S. cerevisiae* for subsequent *in silico* investigations, including molecular docking and molecular dynamics simulations. To this aim, the amino acid sequence of *S. cerevisiae* α-glucosidase was retrieved, and several potential templates were evaluated based on sequence identity, crystallographic resolution, and the presence of co-crystallized ligands within the active site. Among the candidates, the iso-maltase structure of *S. cerevisiae* (PDB ID: 3 A4A) was identified as the most favorable template, exhibiting 72% sequence identity, high resolution, and a bound natural ligand in the catalytic pocket. Consequently, this structure was selected for homology modeling.

**Fig. 4:**
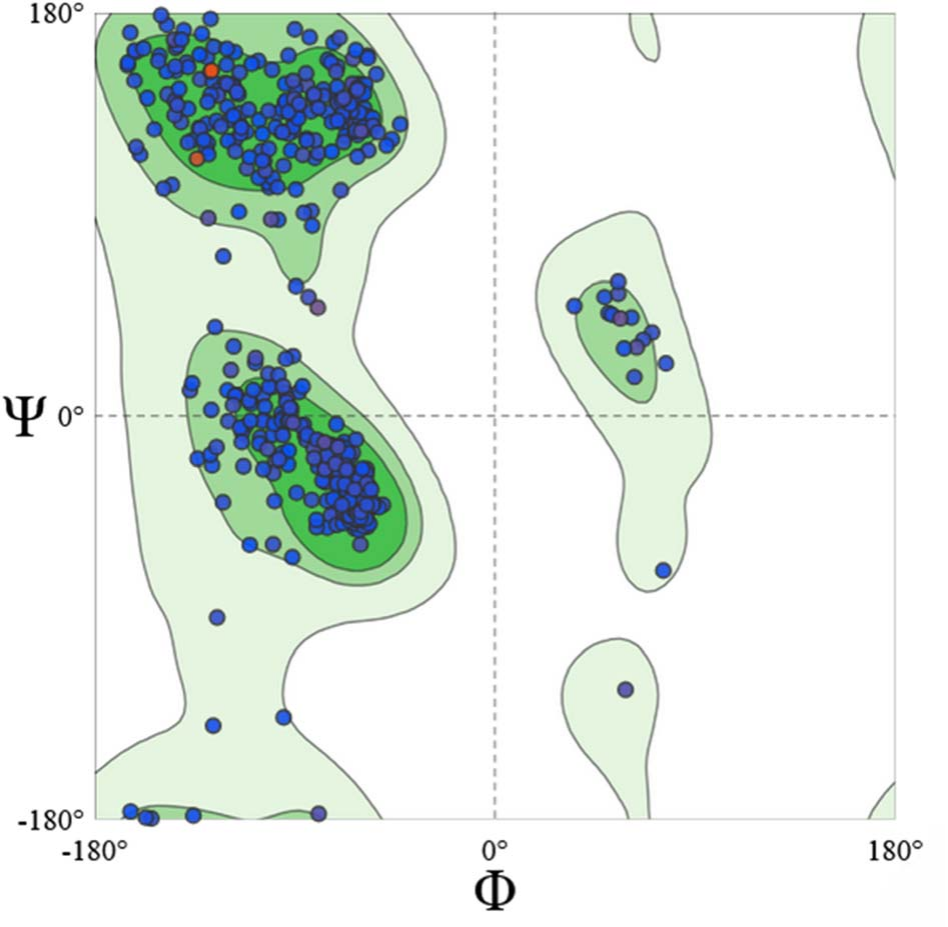
The Ramachandran plot of the homology model built *via* SWISS-MODEL.

The homology model was constructed using the SWISS-MODEL workspace (https://swissmodel.expasy.org/), and its quality was assessed using multiple validation tools. Ramachandran plot analysis performed with PROCHECK (Fig. 4) indicated that more than 96% of the residues were located in the most favored regions, with no residues in disallowed regions. The model exhibited a *z*-score of 0.007 ± 0.35 and a low atomic clash score of 2.14. Furthermore, visual inspection of the overall fold revealed no structural distortions, dissociations, or inconsistencies. Collectively, these results confirmed the structural integrity of the model and its suitability for subsequent computational studies.

To validate the docking protocol, the binding pocket was determined using the natural ligand present in the crystal structure of 3 A4A, following structural alignment between the homology model and the template. The coordinates of the co-crystallized ligand (maltose) from 3 A4A were transferred to the aligned homology model and redocked into the active site, yielding a root-mean-square deviation (RMSD) of 0.56 Å. This result confirmed the reliability of the selected grid box for *in silico* analyses. The final ligand position was used to define the geometric center of the initial search space for molecular docking, encompassing the key catalytic residue Asp214 and the essential active-site residues Glu276 and Asp349.^52,53^

#### 2.3.2 Molecular docking studies

Molecular docking simulations were performed using the constructed homology model. The calculated binding energies and key interacting residues within the active site are summarized in Table 2.

**Table 2:** The molecular docking results: Binding energies and the most important residues contributing to the interactions

Molecule	Binding energy (kcal mol^−1^)	Residues contributing to major interactions		Molecule	Binding energy (kcal mol^−1^)	Residues contributing to major interactions	
12a	−7.81	His279	H-bond	12m	−7.78	Asp408	H-bond
		**Glu276**	H-bond	12l	−8.15	Glu276	H-bond
12b	−7.7	Phe157	H-bond			Gln181	Halogen bond
		Arg 312	H-bond				
		His 279	H-bond	12m	−7.78	Asp408	H-bond
12c	−8.35	Asp408	H-bond				
		**Glu276**	H-bond	12n	−7.65	Phe157	H-bond
12d	−8.07	Arg312	H-bond	12o	−7.22	Arg439	H-bond
		Asp408	H-bond			Phe157	H-bond
		**Glu276**	π-anion			Asp349	Halogen bond
		Asp214	π-anion				
		Asp68	π-anion	12p	−8.2	Phe157	H-bond
12e	−7.88	Arg312	H-bond			Arg312	H-bond
12f	−7.97	**Glu276**	H-bond				
		Asp408	H-bond	12q	−8.09	Arg443	H-bond
		Arg439	π-anion			**Glu276**	H-bond
		Asp349	π-anion			Asn412	H-bond
12g	−8.02	Arg312	H-bond				
		Asp408	H-bond	12r	−8.81	His 111	H-bond
		**Glu276**	H-bond			Arg439	H-bond
		Arg439	π-cation			**Glu276**	H-bond
12h	−6.57	Arg312	H-bond			Arg312	H-bond
		His279	H-bond				
		Asp349	π-anion	13	−7.86	Asp408	H-bond
12i	−7.17	Phe157	H-bond			Phe157	H-bond
		His379	H-bond	14	−8.21	—	
12j	−7.84	His245	H-bond	**Acarbose**	−6.05	Phe157	H-bond
		**Glu276**	H-bond			Asp408	H-bond
		Asn412	H-bond			Arg439	H-bond
		Val277	Halogen bond			**Glu276**	H-bond
						Arg312	H-bond

Analysis of the molecular docking results indicated that all compounds 12a–12r, 13, and 14 were successfully docked into the catalytic pocket of the enzyme, exhibiting favorable predicted binding energies in the range of −6.57 to −8.81 kcal mol^−1^. Notably, the binding energies of the synthesized compounds were significantly lower than that of the reference inhibitor acarbose (−6.05 kcal mol^−1^), which is in great agreement with the experimental inhibitory data. Moreover, a clear correlation was observed between the docking-predicted binding modes and the experimentally observed SAR. Substituent effects on the terminal phenyl ring revealed a distinct trend, wherein *para*-substituted analogues bearing smaller functional groups generally displayed enhanced binding affinities. In contrast, compound 12h, having a bulky tri-methoxy substituent, showed the weakest binding affinity among the series. Conversely, *para*-substitution with small groups led to improved binding energies, lower than −7.5 kcal mol^−1^.

**Fig. 5:**
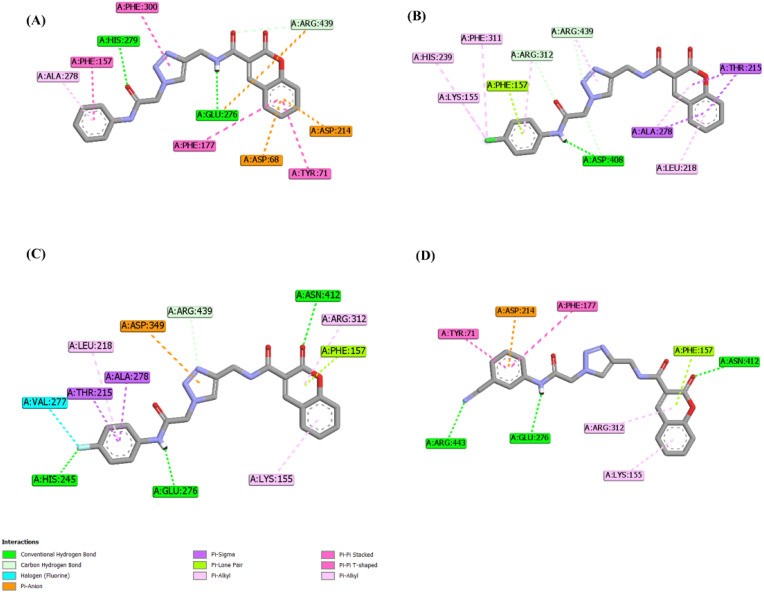
Two-dimensional interaction diagrams of (A) 12a; (B) 12m; (C) 12j; and (D) 12q.

For instance, the interaction diagrams of several of the most active compounds are depicted in Fig. 5. Among the key catalytic residues, Glu276 was found to participate in hydrogen-bond interactions with compounds 12a, 12j, and 12q. As shown in Fig. 5A, compound 12a (IC_50_ = 51.3 µM) was able to form multiple stabilizing interactions through its different sub-scaffolds, namely two hydrogen bonds *via* its amide moieties. These observations suggest that appropriate substitution on the terminal phenyl ring can enhance binding stability and intermolecular interactions. Accordingly, the introduction of a *para*-chloro substituent in compound 12m (IC_50_ = 5.4 µM) enhanced inhibitory activity by enabling several π–alkyl interactions mediated by the chlorine atom. Notably, substitution with fluorine was even more favorable, as observed for compound 12j (IC_50_ = 3.4 µM), due to fluorine's ability to act as a hydrogen-bond acceptor and to engage in halogen bonding. Furthermore, the binding orientation of 12j was altered such that the coumarin moiety formed an additional hydrogen bond with Asn412.

The nitrile group in compound 12q, the most potent inhibitor in the *in vitro* assessments, also functioned as a hydrogen-bond acceptor (Fig. 5D). This compound formed hydrogen-bond interactions with Glu276 through its terminal amide moiety, while preserving the interaction involving the coumarin ring. Overall, compound 12q established three hydrogen bonds along with a π–anion interaction with the catalytic residue Asp214, which rationalizes its superior inhibitory potency (IC_50_ = 1 µM). Furthermore, Tyr71 participated in π–π stacking interactions with the aromatic regions of both 12q and 12a, further stabilizing their binding pose.

**Fig. 6:**
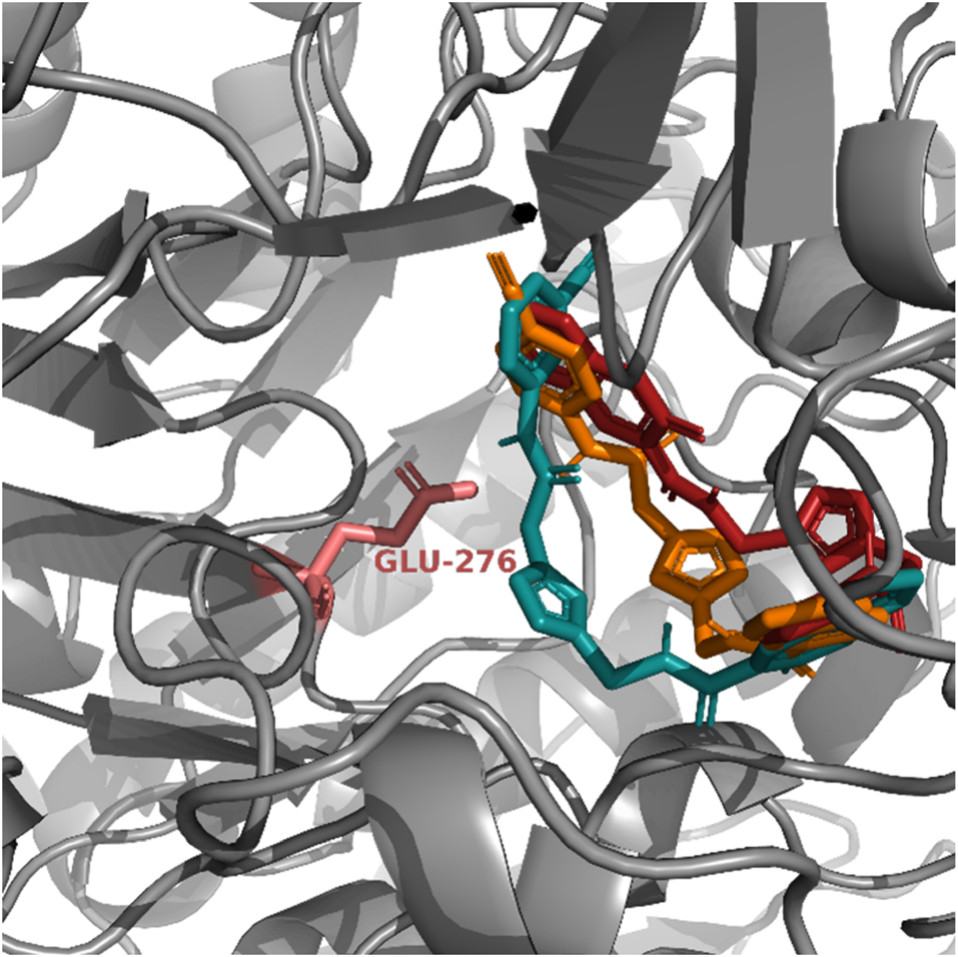
Three-dimensional visualization of the binding pose of 12p (red), 12q (green), 12r (orange) in the active site of the protein.

One plausible explanation for the superior activity of the *meta*-substituted nitrile derivative compared with its *ortho*- and *para*-substituted counterparts (compounds 12p, 12q, and 12r) might be associated with the distinct binding mode of compound 12q. As depicted in Fig. 6, shifting the nitrile group from the *meta* position to the *ortho* or *para* positions alters the ligand orientation within the active site, resulting in an increased distance between the terminal amide moiety and the critical residue Glu276. Therefore, this reduced proximity in the *ortho*- and *para*-substituted analogues likely weakened key stabilizing interactions, thereby losing the inhibitory activity.

**Fig. 7:**
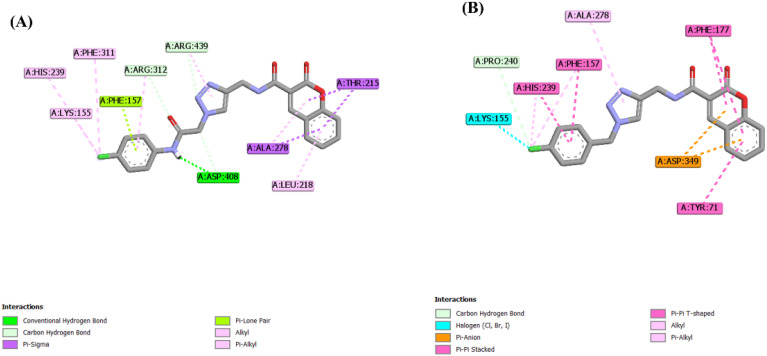
Two-dimensional interaction diagrams of (A) 12m; (B) 14.

As discussed above, the terminal amide moiety emerged as a critical structural feature, capable of forming stabilizing hydrogen bonds through its polar NH group. Removal of this functional group, as observed in compounds 13 and 14, caused remarkable decrease in the number of predicted hydrogen-bond interactions compared with their parent analogues 12a and 12m, respectively. This reduction in key intermolecular interactions was consistent with the experimental findings. For example, Fig. 7 presents a comparison of the predicted interactions of compound 12m (IC_50_ = 5.4 µM) and compound 14 (IC_50_ = 34.2 µM), illustrating that deletion of the terminal amide resulted in diminished *α*-glucosidase inhibitory activity.

#### 2.3.3 Molecular dynamics simulations

Molecular dynamics (MD) simulation was used in order to deepen our understanding of the inhibitory mechanism of 12q against *α*-glucosidase. MD can provide an atomistic view of enzyme–ligand interactions, which can help understand both conformational changes and interaction patterns over time. MD analysis can help reveal the most important binding conformations and the key amino acid residues responsible for ligand's affinity. Moreover, the stability of the enzyme–inhibitor complex can be examined using parameters such as root mean square deviation (RMSD), which offers important data on the complex stability and the evolution of binding poses during the trajectories.^54^

**Fig. 8:**
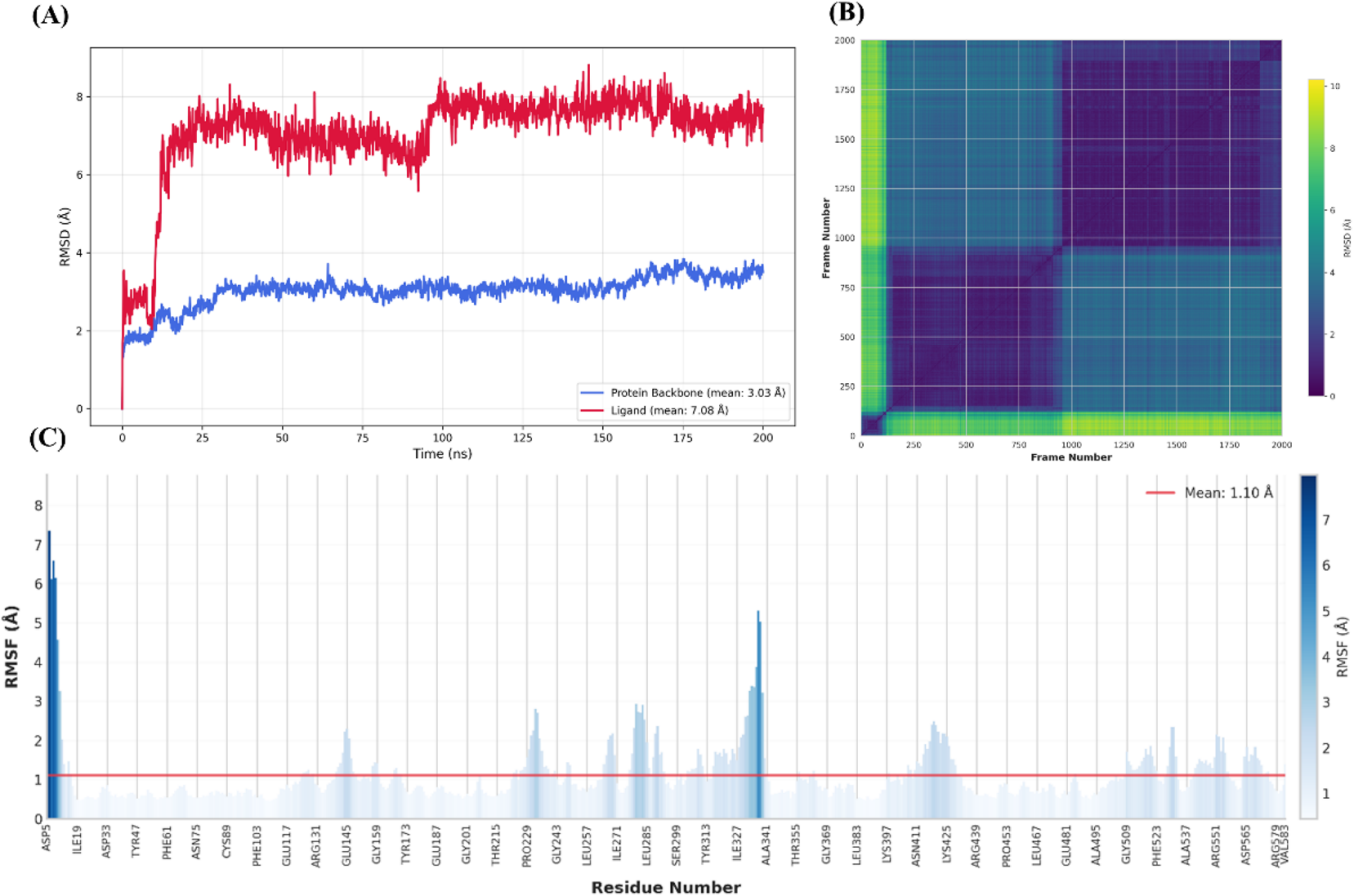
(A) RMSD plot of 12q (red) compared to protein (blue); (B) ligand's RMSD matrix (number of frames was reduced to enhance the visibility); (C) protein's RMSF plot (mean = 1.1 Å).

In this study, MD simulation was performed for the most potent compound 12q, using GROMACS 2025.4, and subsequent trajectory analyses were performed with the MDAnalysis Python package. The protein backbone remained stable throughout the simulation, exhibiting a mean RMSD of 3.03 Å with minimal fluctuations and supporting the overall structural integrity of the constructed homology model (Fig. 8a). Moreover, the Cα RMSF analysis showed a mean value of 1.10 Å, and importantly, none of the residues within the active site displayed elevated RMSF values (Fig. 8c).

In contrast, the ligand demonstrated higher RMSD values overall. A rapid increase in RMSD during the first 25 ns indicated a major adjustment in its binding orientation, likely attributable to the presence of multiple possible binding poses. The RMSD seems to stabilize for the rest of the trajectory. This pattern shows the flexibility of the ligand and its ability to adopt a variety of binding poses in the enzyme active site. RMSD matrix analysis revealed three distinct binding poses during the simulation, with the final pose occupying a larger spatial region, which may be attributed to stronger interactions, particularly with Glu276, as discussed below (Fig. 8b).

**Fig. 9:**
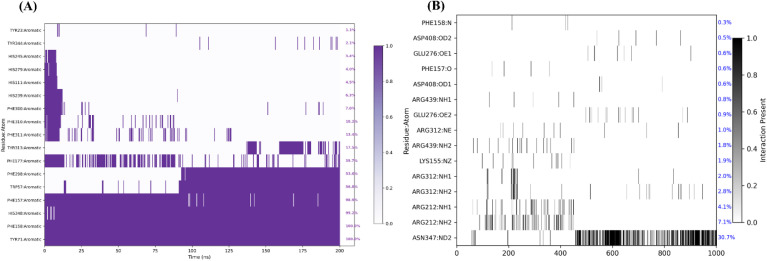
(A) π–π interactions in 200 ns simulation; (B) H-bond persistency diagram in different frames of the simulation (number of frames was reduced to enhance the visibility).

Analysis of the interaction diagrams confirmed, as noted in the docking studies, that aromatic stacking with Tyr71 is a key stabilizing factor. As seen in Fig. 9A, Tyr71, Phe158, His348, and Phe157 acted as persistent aromatic anchors for the ligand, maintaining interactions throughout most of the simulation time. Hydrogen-bond persistence analysis (Fig. 9B) showed a dynamic shift in interacting residues over time, consistent with the changes in ligand binding poses observed in the RMSD matrix. Towards the end of the simulation, Asn347, Arg312, and particularly Glu276, which was previously identified as a critical residue, emerged as the primary contributors to ligand stabilization. In contrast, during the initial phase, Arg212 and Arg349 played a more prominent role in maintaining hydrogen-bond interactions and stabilizing the ligand within the binding pocket.

To conclude, several key residues initially identified in the docking studies as potentially important were further validated by molecular dynamics simulations, demonstrating their ability to form persistent interactions. These residues included Tyr71, Glu276, Phe157, Arg312, Arg439, and Asp408. Interestingly, these were mostly the same residues contributing to anchor the reference drug acarbose in the molecular docking studies. Together, the molecular docking and dynamics data provided probable mechanism of binding for the ligand in the active site.

Given the noticeable α-glucosidase inhibitory potency of compound 12q, as thoroughly confirmed by *in vitro* assessments and *in silico* studies, it was selected as the lead candidate for further evaluations in this work.

### 2.4 α-Glucosidase kinetic studies

**Fig. 10:**
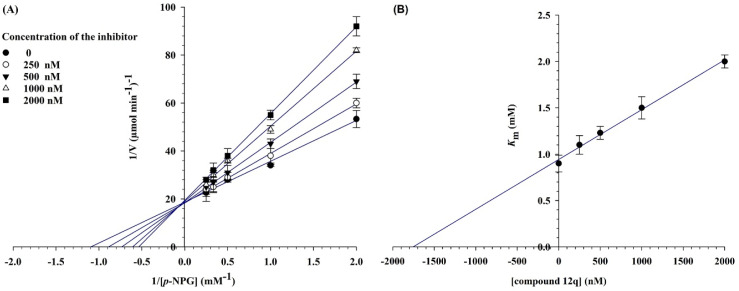
α-Glucosidase kinetic studies compound 12q: (A) the Lineweaver–Burk plot using various concentrations of the inhibitor; (B) the secondary plot between *K*_m_ and various concentrations of the inhibitor.

The kinetic studies of α-glucosidase inhibition were conducted on the most active compound 12q to gain further information about its mechanism. The inhibition assessments were performed in the presence of the standard substrate (*p*-nitrophenyl α-d-glucopyranoside) at different concentrations (ranging from 1 to 16 mM) and our selected inhibitor (compound 12q) at a variety of concentrations (including 0, 250, 500, 1000, and 2000 nM). The results were employed to outline the Lineweaver–Burk plot and determine the *V*_max_ and *K*_m_ values. As depicted in Fig. 10A, the *K*_m_ value increased with increasing concentrations of compound 12q, while *V*_max_ remained constant, revealing competitive inhibition. These results confirmed that our inhibitor competed with the substrate for binding to the active site of α-glucosidase. Further evaluation of the *K*_m_ plot, which was depicted using the *K*_m_ values against different concentrations of compound 12q (Fig. 10B), helped determine the binding constant (*K*_i_) and assess the inhibitor's binding affinity. *K*_i_ was determined to be 1000 nM, revealing the strong binding affinity of compound 12q with the α-glucosidase active site.

### 2.5 α-Amylase inhibitory activity

α-Amylase is an enzyme present in the saliva and pancreas, which manages the early steps of carbohydrate digestion and hydrolyzes complex carbohydrates into oligosaccharides.^55^ On the other hand, α-glucosidase, located at the intestinal brush border, catalyzes the final step of carbohydrate digestion and directly controls the rate of glucose absorption. α-Amylase inhibition can be associated with gastrointestinal adverse effects such as bloating, flatulence, and diarrhea that can be due to increased oligosaccharides in the colon and its fermentation by gut microbiota.^56,57^ α-Amylase inhibitory assessment was conducted for the compound 12q, showing no activity at concentrations up to 100 µM. This selectivity may reduce the possible risk of the gastrointestinal side effects of this α-glucosidase inhibitor for our further *in vivo* anti-diabetic investigations.

### 2.6 Circular dichroism (CD) spectroscopy assessment

Circular Dichroism (CD) spectroscopy is a powerful analytical technique used to investigate the secondary structure and conformational changes of chiral molecules, particularly enzymes. It is frequently employed to determine whether an inhibitor binding leads to structural rearrangements in the enzyme and its catalytic site. Among the various CD measurement types, scans recorded in the far-ultraviolet (far-UV) region (between 190 nm to 240 nm) are particularly informative, providing essential insights into the impact of inhibitor binding on the enzyme's secondary structure.^58^

The Far-UV CD spectroscopy results exhibited the variations in proportion of enzyme composition, including α-helix, β-sheet (or extended β-structure), β-turn, and random coil (which corresponds to unordered or disordered regions). As presented in Table 3, the binding of compound 12q induced only subtle conformational changes in α-glucosidase, with a slight increase in α-helical content (from 0% to 4.3%) and a small decrease in random coil structures (from 60% to 52.1%). The overall secondary structure profile remains largely intact. This observation in addition to the competitive inhibition mechanism confirmed that compound 12q bound directly to the enzyme's active site without inducing major global structural rearrangements. Moreover, the minor increase in structural order likely attributed to the local stabilization of flexible loops or regions adjacent to the binding pocket upon the inhibitor's presence.

**Table 3:** CD Results^a^

Entry	α-helix	β-sheet	β-turn	Random coils
1	0	32.4	7.6	60
2	4.3	33.0	10.6	52.1

a All numbers are expressed as percentages; condition 1 was recorded with native α-glucosidase, and condition 2 was measured using α-glucosidase in the presence of compound 12q.

### 2.7 Fluorescence spectroscopy measurements

Changes in the intrinsic fluorescence of α-glucosidase were observed upon addition of compound 12q, suggesting an interaction between the enzyme and the ligand. However, fluorescence measurements can be influenced by spectral artifacts such as the inner filter effect or ligand background emission. This compound displayed measurable native emission in the 300–450 nm region, overlapping with the enzyme signal; therefore, all spectra were blank corrected using the fluorescence of 12q recorded independently at the same concentrations.

To evaluate possible optical distortions, the absorbance of the α-glucosidase-12q mixtures was monitored at 280 nm (excitation) and around 340 nm (emission). The values were found to be lower than 0.1 under all tested conditions. According to established fluorescence spectroscopy guidelines, absorbance values < 0.1 correspond to negligible IFE, and consequently no further correction using was necessary. Therefore, the fluorescence changes observed after blank subtraction genuinely reflect enzyme–ligand binding interactions rather than optical artifacts associated with compound emission or IFE.

1 *F*_corr_ = *F*_obs_ × 10^[(*A*_ex_+*A*_em_)/2]^

Collectively, the inhibitor bound to α-glucosidase in a 1 : 1 stoichiometry with high affinity (*K*_d_ ≈ 0.5 µM). Thermodynamic analysis revealed a spontaneous, entropy-driven binding process, characteristic of hydrophobic interactions. These findings provided a quantitative and mechanistic basis for the inhibitory activity of compound 12q and supported further structure-based optimization of this compound.

**Fig. 11:**
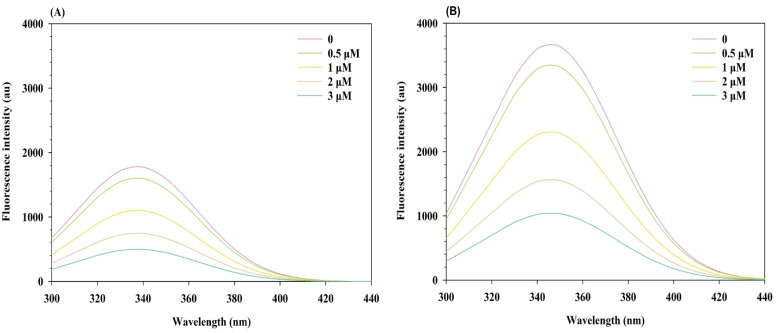
Fluorescence spectra of α-glucosidase in the presence of compound 12q (A) at 25 °C; and (B) at 35 °C.

The interaction between the inhibitor and α-glucosidase was quantitatively evaluated through fluorescence quenching analysis. A progressive decrease in the intrinsic fluorescence intensity of α-glucosidase (*λ*_ex_ = 280 nm, *λ*_em_ = 340 nm) was observed with increasing inhibitor concentration (0–3 µM), confirming effective complex formation (Fig. 11).

The number of binding sites (*n*) and the binding constant (*K*_a_) were determined using the double logarithmic form of the static quenching equation:

2 Log((*F*_0_ − *F*)/*F*) = Log *K*_a_ + *n* Log[*Q*]

A linear regression of Log((*F*_0_ − *F*)/*F*) *versus* Log[*Q*] yielded a slope corresponding to *n* and an intercept representing Log *K*_a_. The calculated value of *n* was approximately 1.0, indicating that a single molecule of inhibitor binds to one molecule of α-glucosidase under the experimental conditions.

The dissociation constant (*K*_d_) was also achieved using the binding isotherm equation:

3 (*F*_0_ − *F*)/(*F* − *F*_min_) = [*Q*]/*K*_d_

**Fig. 12:**
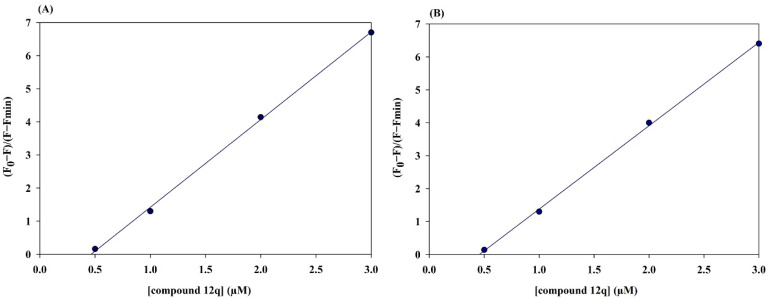
Linear fitting of (*F*_0_ − *F*)/(*F* − *F*_min_) *versus* concertation of compound 12q (A) at 25 °C; and (B) at 35 °C.

Linear fitting of (*F*_0_ − *F*)/(*F* − *F*_min_) *versus* [*Q*] gave *K*_d_ values of 0.50 µM at 25 °C and 0.51 µM at 35 °C, reflecting strong and consistent binding affinity across the studied temperature range (Fig. 12).

The corresponding association constants were *K*_a_ = 2.0 × 10^6^ M^−1^ at 25 °C and *K*_a_ = 1.95 10^6^ M^−1^ at 35 °C. These results confirm a 1 : 1 stoichiometry and high-affinity binding of the inhibitor to α-glucosidase, with *K*_d_ in the sub-micromolar range. The temperature stability of this parameter suggested a robust interaction, consistent with the thermodynamic analysis presented in the following section.

In the next step, thermodynamic parameters were obtained from the temperature dependence of *K*_a_ using the van't Hoff equation:

4 Ln *K*_a_ = −(Δ*H*°/*R*) × (1/*T*) + (Δ*S*°/*R*)

5 Δ*G*^∘^ = −RT Ln *K*_a_

The results were summarized in Table 4:

**Table 4:** Thermodynamic parameters of compound 12q

Temperature	Δ*H*°	Δ*S*°	Δ*G*°
25 °C	−1.93 kJ mol^−1^	+114.2 J mol^−1^ K^−1^	−35.97 kJ mol^−1^
35 °C			−37.08 kJ mol^−1^

The near-unity value of *n* and the consistency of *K*_d_ across temperatures indicated a single, high-affinity binding site. The negative Δ*G*° confirmed spontaneous binding, while the small negative Δ*H*° and large positive Δ*S*° suggested an entropy-driven interaction, likely dominated by hydrophobic effects and desolvation of the binding interface.

### 2.8 *In vivo* anti-diabetic studies of compound 12q

#### 2.8.1 Acute oral toxicity test

The acute oral toxicity of the compound 12q was evaluated using the OECD Guideline 423 (Acute Toxic Class Method). No mortality or signs of clinical toxicity (including toxic symptoms and adverse effects such as diarrhea, sedation, convulsions, lethargy, tremors, or excessive salivation) were observed at doses of 5, 50, and 300 mg kg^−1^ body weight (BW). However, administration of higher doses (500 and 2000 mg kg^−1^) resulted in mortality in all animals. Therefore, the No Observed Adverse Effect Level (NOAEL) for compound 12q was 300 mg kg^−1^ BW, and the Lowest Observed Adverse Effect Level (LOAEL) was 500 mg kg^−1^ BW.

#### 2.8.2 Fasting blood glucose levels

Mice (*n* = 8 per group, minimum) were randomly categorized into six groups: a normal control (group 1), an untreated diabetic control (group 2), three diabetic groups treated orally with compound 12q at doses of 15 (group 3), 10 (group 4), and 5 mg kg^−1^ BW (group 5), and a positive control group treated with acarbose at dose of 25 mg kg^−1^ BW (group 6). Subsequently, type 2 diabetes was successfully induced in adult male C57BL/6J mice using a combination of a high-fat diet for six weeks followed by multiple low-dose intraperitoneal injections of streptozotocin (STZ; 30 mg kg^−1^ for 5 consecutive days). Induction was confirmed by measuring fasting blood glucose (FBG) levels. Mice with FBG levels greater than 200 mg dL^−1^ were included in the diabetic groups. To evaluate the FBG levels, mice were fasted for 6 h.

**Fig. 13:**
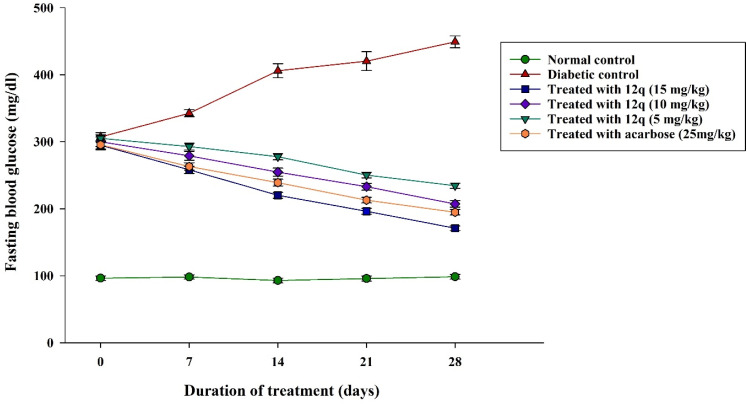
FBG levels (mg dL^−1^) in 28-days experiment. The data are expressed as the mean ± SEM (*n* = 8 per group).

As presented in Fig. 13, the normal control mice (group 1) exhibited the stable normoglycemic values throughout the study (approximately 93–99 mg dL^−1^). At day 0, diabetic mice (groups 2–6) exhibited remarkably high FBG levels (approximately 295–305 mg dL^−1^), with no remarkable differences among the diabetic groups, confirming a state of stable hyperglycemia and indicating successful and homogeneous induction of diabetes in this experimental model. The FBG levels in diabetic control mice (group 2) increased progressively during the study, rising from 307.25 ± 6.54 mg dL^−1^ at day 0 to 449.25 ± 8.84 mg dL^−1^ at day 28, which approved persistent and worsening hyperglycemia in the absence of treatment.

Oral administration of compound 12q resulted in a significant, dose-dependent reduction in FBG levels compared to the diabetic control group. At the highest dose (15 mg kg^−1^ BW, group 3), compound 12q led to a sustained decrease in FBG, reducing glucose levels from 294.88 ± 6.68 mg dL^−1^ at day 0 to 170.88 ± 3.51 mg dL^−1^ on day 28, corresponding to an approximate 42% reduction. This significant decrease was already evident by day 7 and continued progressively throughout the treatment period. Oral administration of compound 12q at 10 mg kg^−1^ BW (group 4) also significantly lowered FBG levels in diabetic mice, as FBG level decreased from 300.13 ± 7.07 mg dL^−1^ at day 0 to 207.13 ± 4.76 mg dL^−1^ at day 28, representing an approximate 31% reduction. Similarly, mice administered lowest dose of compound 12q (5 mg kg^−1^ BW, group 5) exhibited a moderate yet significant decrease in FBG, from 305.25 ± 5.32 mg dL^−1^ to 234.50 ± 3.86 mg dL^−1^ over the 28-days period (approximately 23% reduction). The reference drug acarbose (25 mg kg^−1^ BW, group 6) significantly reduced FBG levels from 295.63 ± 6.28 mg dL^−1^ at day 0 to 194.75 ± 4.04 mg dL^−1^ at day 28, corresponding to a 34% decrease. Notably, compound 12q at 15 mg kg^−1^ BW exhibited superior antihyperglycemic efficacy compared to acarbose, while the 10 mg kg^−1^ BW dose produced comparable effects.

Overall, these results revealed that compound 12q can act as a potent and dose-dependent antihyperglycemic agent in diabetic C57BL/6J mice, effectively decreasing fasting hyperglycemia over the 4-weeks treatment period.

#### 2.8.3 Oral sucrose tolerance test (OSTT)

**Fig. 14:**
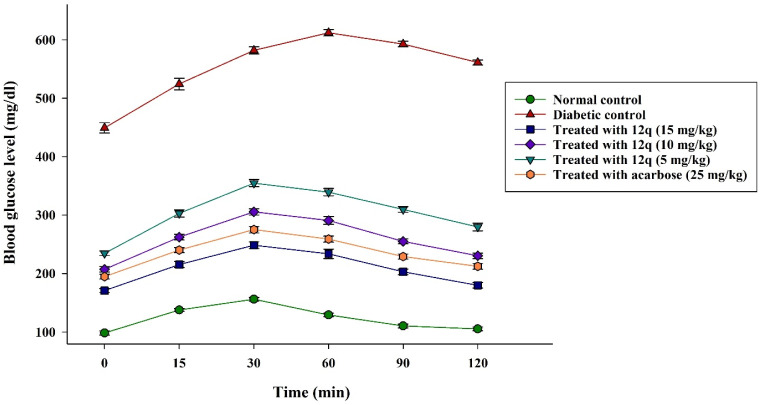
Blood glucose levels (mg dL^−1^) in OSTT assessment.

The glucose-lowering efficacy of compound 12q was investigated by the oral sucrose tolerance test (OSTT) performed on day 28. As presented in Fig. 14, glucose levels in healthy control mice increased transiently following oral glucose administration and rapidly returned toward baseline values within 120 min, reflecting normal glucose tolerance. This was further supported by a low AUC value (15 094.8 mg min dL^−1^). On the other hand, diabetic control mice exhibited severe impairment in glucose tolerance, characterized by remarkably high fasting glucose levels and a failure to return toward baseline during the observation period. Accordingly, the AUC in this group was dramatically increased (68 900.6 mg min dL^−1^), indicating persistent hyperglycemia and defective glucose clearance.

Treatment with compound 12q significantly improved glucose tolerance in a dose-dependent manner. Mice treated with the highest dose of this compound (group 3) displayed a rise in postprandial glucose levels, a reduced peak glucose concentration, and an accelerated decline toward baseline. This improvement was quantitatively confirmed by a substantial reduction in AUC (25 904.1 mg min dL^−1^), corresponding to an approximately 62% decrease compared with that of diabetic control mice. Groups 4 and 5 also showed reductions in AUC values (32 182.6 and 37 942.5 mg min dL^−1^, respectively), although these effects were less significant than those observed with the highest dose. Group 6 exhibited a significant improvement in glucose tolerance, as evidenced by reduced postprandial glucose levels and a lower AUC (29 076.7 mg min dL^−1^) compared with diabetic controls. Notably, the glucose-lowering effect of compound 12q at the highest dose (group 3) was comparable to, and slightly greater than, that of acarbose, indicating a potent inhibitory effect of this compound at this dose on postprandial glucose absorption. These results were consistent with the strong α-glucosidase inhibitory activity of compound 12q observed in*vitro* evaluations.

## 3 Conclusion

In this study, we successfully identified twenty derivatives of coumarin–triazole hybrids as promising anti-diabetic agents through α-glucosidase inhibition. Among them, the lead compound, 12q, demonstrated noticeable *in vitro* potency (IC_50_ = 1.0 µM) with a competitive inhibition mechanism. *K*_i_ was determined to be 1000 nM, revealing the strong binding affinity of compound 12q with the α-glucosidase active site. Detailed SAR analysis indicated that small electron-withdrawing substituents at the *para* or *meta* positions of the terminal phenyl ring enhanced enzyme inhibition. The superiority of the *meta*-nitril group was explained by its planar nature, which is distinguishable from the other derivatives. The importance of the terminal amide moiety was also investigated. The SAR trend was subsequently supported by molecular docking analyses. In molecular dynamics, stable and critical interactions with Glu276, Asp214, and Tyr71 were observed, providing a rationale for compound 12q's exceptional potency. Furthermore, 12q exhibited excellent selectivity over α-amylase and demonstrated significant *in vivo* antihyperglycemic effects, outperforming acarbose. These findings reveal the potential of this scaffold as a safe and effective starting point for further research on α-glucosidase inhibitor molecules. While the results are encouraging, further studies on its long-term pharmacokinetics and safety profile are needed. For example, comprehensive toxicological evaluation will be required before this compound can be considered a drug candidate. These studies will be planned as part of the next stage of development. Overall, compound 12q represents a valuable scaffold for the development of next-generation antidiabetic agents.

## 4 Experimental

### 4.1 Material and methods

All chemicals were purchased from Merck (Germany) and were used without further purification. The reaction progress and the purity of synthesized compounds were monitored by thin-layer chromatography (TLC) on silica gel 250-micron F254 plastic sheets; zones were detected visually under UV light (254 nm). Melting points were measured on an Electrothermal 9100 apparatus. IR spectra were recorded on a Shimadzu IR-460 spectrometer. ^1^H and ^13^C NMR spectra were measured (DMSO-*d*_6_ solution) with Bruker DRX-400 AVANCE (at 400.1 and 100.1 MHz) and Bruker DRX-500 AVANCE (at 500.1 and 125.8 MHz) instruments. Chemical shifts were reported in parts per million (ppm), downfield from tetramethylsilane (TMS). Proton coupling patterns were described as singlet (s), doublet (d), triplet (t), and multiplet (m). HRMS analysis was performed using a Waters Synapt G1 HDMS High Definition mass spectrometer equipped with an electrospray ionization (ESI) source. The samples were prepared by diluting the isolated compounds in methanol to a final concentration of 10 µg mL^−1^. The analysis was conducted in negative ion mode with a mass range of *m*/*z* 50–1000. Elemental analyses for C, H and N were performed using a Heraeus CHN-O-Rapid analyzer.

### 4.2 General synthetic procedures

#### 4.2.1 General procedure for the preparation of ethyl 2-oxo-2*H*-chromene-3-carboxylate 3

A mixture of salicylaldehyde 1 (1.0 equiv.), diethyl malonate 2 (1.2 equiv.), and piperidine (0.5 equiv.) in absolute ethanol was heated under reflux conditions for 4 h. After completion of the reaction as confirmed by TLC analysis, the mixture was allowed to cool to room temperature and then quenched by the addition of ice–water. The resulting precipitate was filtered and washed thoroughly with water to afford the desired adduct as a pale-yellow solid in the yield of 80%.

#### 4.2.2 General procedure for the preparation of 2-oxo-2*H*-chromene-3-carboxylic acid 4

Compound 3 (1.0 equiv.) was dissolved in a 1 : 1 (v/v) mixture of ethanol and 10% aqueous sodium hydroxide solution (4.0 equiv.) and heated under reflux conditions for 2 h. Upon completion of the reaction, the mixture was cooled in ice bath and acidified dropwise with 10% hydrochloric acid until precipitation occurred. The resulting white solid was collected by filtration and washed thoroughly with water to afford 2-oxo-2*H*-chromene-3-carboxylic acid 4 adduct as a white solid in the yield of 75%.

#### 4.2.3 General procedure for the preparation of 2-oxo-*N*-(prop-2-yn-1-yl)-2*H*-chromene-3-carboxamide 6

A mixture of acidic adduct 4 (1.0 equiv.), 2-(1*H*-benzotriazol-1-yl)-1,1,3,3-tetramethylaminium tetrafluoroborate (TBTU, 1.5 equiv.), and diisopropylethylamine (DIPEA, 1.0 equiv.) in dimethylformamide (DMF) was stirred at room temperature for 2 h (flask A). In parallel, propargylamine 5 (0.7 equiv.) and DIPEA (1.0 equiv.) were dissolved in DMF and stirred at room temperature for 2 h (flask B). The contents of flask B were added dropwise to flask A, and the resulting reaction mixture was stirred at room temperature overnight. After completion of the reaction, the mixture was quenched with ice–water, and the resulting precipitate was collected by filtration and washed thoroughly with water. The residue was recrystallized with EtOH to afford compound 6 as a pure, light-yellow solid in the yield of 58%.

#### 4.2.4 General procedure for the preparation of 2-choloro-*N*-arylacetamides 9

To a mixture of different aniline derivatives 7 (1.0 equiv.) and triethylamine (1.2 equiv.) in acetone, chloroacetyl chloride 8 (1.2 equiv.) was added dropwise at 0 °C within 10 min. The reaction mixtures were then stirred overnight at room temperature. After completion of the reaction, the mixture was poured on ice-water and stirred to precipitate completely. The resulting solid was filtered and washed with sufficient amount of water to give pure desired compounds 9.

#### 4.2.5 General procedure for the preparation of 2-azido-*N*-phenylacetamides 11

A mixture of previously prepared compounds 9 (1.0 equiv.) and sodium azide 10 (1.5 equiv.) were dissolved in DMF and stirred at room temperature overnight. Upon completion of the reactions, the reaction mixtures were poured into water, and the resulting precipitates were collected by filtration and washed thoroughly with water, affording pure desired compounds 11.

#### 4.2.6 General procedure for the preparation of the targeted compounds 12, 13, and 14

The corresponding benzyl chloride derivative was stirred with sodium azide in DMF at room temperature for 1 h to produce the corresponding benzyl azide *in situ*. The resulting mixture was used directly in the next step without any workup or isolation.

A mixture of benzyl azide or 2-azido-*N*-phenylacetamides 11 (1.0 equiv.), along with compound 6 (1.2 equiv.), CuSO_4_·5H_2_O (0.3 equiv.), and sodium ascorbate (0.3 equiv.) in DMF was stirred magnetically at room temperature for an overnight. After complete consumption of the starting materials as confirmed by TLC analysis, water was added to the reaction mixture, and stirring was continued until full precipitation formed. The resulting solid was collected by filtration, washed thoroughly with water, and finally washed with ethyl acetate to yield the target compounds 12, 13, or 14 as milky or yellow powders.

##### 4.2.6.1 2-Oxo-*N*-((1-(2-oxo-2-(phenylamino)ethyl)-1*H*-1,2,3-triazol-4-yl)methyl)-2*H*-chromene-3-carboxamide (12a)

Milky solid, yield 78%, m.p.: 199–202 °C. FT/IR (*ν*_max_/cm^−1^): 3314 and 3056 (2NH), 1735 (C=O, coumarin), 1666 and 1647 (C=O, amide), 1610, 1601, 1569, 1545, 1499, 1455, 1446, 1369, 1332, 1311, 1298, 1267, 1153, 1145, 1137, 1078, 1052, 1032, 967, 799, 760, 749, 690. ^1^H NMR (500.1 MHz, DMSO-*d*_6_): *δ* 10.45 (s, 1H, NH-amide), 9.14 (br. s, 1H, CH_2_NH), 8.89 (s, 1H, CH), 8.18–6.89 (m, 10H, 10CH), 5.31 (s, 2H, CH_2_), 4.62 (br. s, 2H, CH_2_NH). ^13^C NMR (125.8 MHz, DMSO-*d*_6_): *δ* 164.16, 161.08, 160.32, 153.89, 147.66, 143.96, 138.37, 134.13, 130.26, 129.80, 128.85, 125.11, 123.73, 119.20, 118.72, 118.41, 116.11, 52.19, 34.88. HRMS (ESI) *m*/*z* for C_21_H_18_N_5_O_4_^+^ [M + H]^+^, calculated: 404.1353, found: 404.1356. Anal. Calcd. for C_21_H_17_N_5_O_4_ : C, 62.53; H, 4.25; N, 17.36; found: C, 62.60; H, 4.37; N, 17.35%.

##### 4.2.6.2 2-Oxo-*N*-((1-(2-oxo-2-(*o*-tolylamino)ethyl)-1*H*-1,2,3-triazol-4-yl)methyl)-2*H*-chromene-3-carboxamide (12b)

Milky solid, yield 70%, m.p.: 166–169 °C. ^1^H NMR (400.1 MHz, DMSO-*d*_6_): *δ* 9.79 (s, 1H, NH-amide), 9.17 (br. s, 1H, CH_2_N*H*), 8.92 (s, 1H, CH), 8.08 (s, 1H, CH), 8.01 (d, *J* = 7.4 Hz, 1H, CH), 7.76 (t, *J* = 7.6 Hz, 1H, CH), 7.52 (d, *J* = 8.2 Hz, 1H, CH), 7.46 (t, *J* = 7.4 Hz, 1H, CH), 7.44 (t, *J* = 7.6 Hz, 1H, CH), 7.23 (d, *J* = 7.1 Hz, 1H, CH), 7.17 (t, *J* = 7.3 Hz, 1H, CH), 7.10 (t, *J* = 7.1 Hz, 1H, CH), 5.38 (s, 2H, CH_2_), 4.63 (br. s, 2H, CH_2_NH), 2.23 (s, 3H, CH_3_). ^13^C NMR (100.1 MHz, DMSO-*d*_6_): *δ* 164.87, 161.57, 160.83, 154.37, 148.19, 141.77, 135.95, 134.66, 132.00, 130.89, 130.79, 129.22, 126.52, 126.00, 125.63, 125.16, 119.20, 118.91, 116.63, 52.38, 35.37, 18.28. HRMS (ESI) *m*/*z* for C_22_H_20_N_5_O_4_^+^ [M + H]^+^, calculated: 418.1510, found: 418.1512. Anal. Calcd. for C_22_H_19_N_5_O_4_ : C, 63.30; H, 4.59; N, 16.78; found: C, 63.38; H, 4.76; N, 16.94%.

##### 4.2.6.3 2-Oxo-*N*-((1-(2-oxo-2-(*m*-tolylamino)ethyl)-1*H*-1,2,3-triazol-4-yl)methyl)-2*H*-chromene-3-carboxamide (12c)

Milky solid, yield 82%, m.p.: 152–154 °C. FT/IR (*ν*_max_/cm^−1^): 3318 and 3146 (2NH), 1704 (C=O, coumarin), 1669 and 1654 (C=O, amide), 1567, 1520, 1490, 1454, 1364, 1295, 1211, 1120, 1051, 962, 819, 796, 759, 690. ^1^H NMR (400.1 MHz, DMSO-*d*_6_): *δ* 10.40 (s, 1H, NH-amide), 9.18 (br. s, 1H, CH_2_NH), 8.92 (s, 1H, CH), 8.09 (s, 1H, CH), 8.01 (d, *J* = 7.4 Hz, 1H, CH), 7.76 (t, *J* = 7.6 Hz, 1H, CH), 7.52 (d, *J* = 8.2 Hz, 1H, CH), 7.45 (t, *J* = 7.4 Hz, 1H, CH), 7.41 (s, 1H, CH), 7.36 (d, *J* = 7.8 Hz, 1H, CH), 7.20 (t, *J* = 7.7 Hz, 1H, CH), 6.90 (d, *J* = 7.3 Hz, 1H, CH), 5.32 (s, 2H, CH_2_), 4.64 (br. s, 2H, CH_2_NH), 2.27 (s, 3H, CH_3_). ^13^C NMR (100.1 MHz, DMSO-*d*_6_): *δ* 164.57, 161.58, 160.83, 154.38, 148.21, 141.65, 138.79, 138.58, 134.65, 130.79, 129.21, 128.37, 125.63, 124.92, 120.17, 119.19, 118.91, 116.82, 116.62, 52.71, 35.37, 21.63. HRMS (ESI) *m*/*z* for C_22_H_20_N_5_O_4_^+^ [M + H]^+^, calculated: 418.1510, found: 418.1510. Anal. Calcd. for C_22_H_19_N_5_O_4_ : C, 63.30; H, 4.59; N, 16.78; found: C, 63.42; H, 4.67; N, 16.90%.

##### 4.2.6.4 2-Oxo-*N*-((1-(2-oxo-2-(*p*-tolylamino)ethyl)-1*H*-1,2,3-triazol-4-yl)methyl)-2*H*-chromene-3-carboxamide (12d)

Pale yellow solid, yield 89%, m.p. 181–183 °C. ^1^H NMR (500.1 MHz, DMSO-*d*_6_): *δ* 10.31 (s, 1H, NH-amide), 9.14 (br. s, 1H, CH_2_NH), 8.89 (s, 1H, CH), 8.10 (s, 1H, CH), 7.99 (d, *J* = 6.4 Hz, 1H, CH), 7.76 (t, *J* = 6.0 Hz, 1H, CH), 7.54–7.40 (m, 4H, 4CH), 7.13 (d, *J* = 6.9 Hz, 2H, 2CH), 5.31 (s, 2H, CH_2_), 4.64 (br. s, 2H, CH_2_NH), 2.25 (s, 3H, CH_3_). ^13^C NMR (125.8 MHz, DMSO-*d*_6_): *δ* 164.25, 161.68, 160.76, 154.37, 148.21, 141.82, 136.33, 134.66, 133.21, 130.78, 129.73, 129.21, 125.61, 119.66, 119.15, 118.88, 116.61, 52.83, 35.31, 20.90. HRMS (ESI) *m*/*z* for C_22_H_20_N_5_O_4_^+^ [M + H]^+^, calculated: 418.1510, found: 418.1516. Anal. Calcd. for C_22_H_19_N_5_O_4_ : C, 63.30; H, 4.59; N, 16.78; found: C, 63.38; H, 4.71; N, 16.87%.

##### 4.2.6.5 *N*-((1-(2-((2-methoxyphenyl)amino)-2-oxoethyl)-1*H*-1,2,3-triazol-4-yl)methyl)-2-oxo-2*H*-chromene-3-carboxamide (12e)

Pale yellow solid, yield 65%, m.p. 161–163 °C. ^1^H NMR (400.1 MHz, DMSO-*d*_6_): *δ* 9.74 (s, 1H, NH-amide), 9.19 (br. s, 1H, CH_2_N*H*), 8.92 (s, 1H, CH), 8.12 (s, 1H, CH), 8.00 (d, *J* = 7.2 Hz, 1H, CH), 7.92 (d, *J* = 7.7 Hz, 1H, CH), 7.76 (t, *J* = 7.4 Hz, 1H, CH), 7.52 (d, *J* = 8.1 Hz, 1H, CH), 7.45 (t, *J* = 7.2 Hz, 1H, CH), 7.10 (t, *J* = 8.1 Hz, 1H, CH), 7.08 (d, *J* = 7.8 Hz, 1H, CH), 6.90 (t, *J* = 6.9 Hz, 1H, CH), 5.43 (s, 2H, CH_2_), 4.63 (br. s, 2H, C*H*_2_NH), 3.86 (s, 3H, OCH_3_). ^13^C NMR (100.1 MHz, DMSO-*d*_6_): *δ* 164.90, 161.58, 160.83, 154.37, 149.98, 148.19, 141.75, 134.65, 130.78, 129.26, 126.99, 125.62, 125.33, 122.12, 120.74, 119.19, 118.91, 116.62, 111.71, 56.16, 52.73, 35.45. HRMS (ESI) *m*/*z* for C_22_H_20_N_5_O_5_^+^ [M + H]^+^, calculated: 434.1459, found: 434.1466. Anal. Calcd. for C_22_H_19_N_5_O_5_ : C, 60.97; H, 4.42; N, 16.16; found: C, 60.89; H, 4.41; N, 16.27%.

##### 4.2.6.6 *N*-((1-(2-((3-methoxyphenyl)amino)-2-oxoethyl)-1*H*-1,2,3-triazol-4-yl)methyl)-2-oxo-2*H*-chromene-3-carboxamide (12f)

Pale yellow solid, yield 82%, m.p. 173–175 °C. ^1^H NMR (500.1 MHz, DMSO-*d*_6_): *δ* 10.43 (s, 1H, NH-amide), 9.15 (br. s, 1H, CH_2_N*H*), 8.89 (s, 1H, CH), 8.09 (s, 1H, CH), 7.98 (d, *J* = 7.1 Hz, 1H, CH), 7.74 (t, *J* = 7.4 Hz, 1H, CH), 7.49 (d, *J* = 8.1 Hz, 1H, CH), 7.43 (t, *J* = 7.0 Hz, 1H, CH), 7.27 (s, 1H, CH), 7.21 (t, *J* = 8.0 Hz, 1H, CH), 7.08 (d, *J* = 7.5 Hz, 1H, CH), 6.65 (d, *J* = 7.2 Hz, 1H, CH), 5.31 (s, 2H, CH_2_), 4.63 (br. s, 2H, CH_2_NH), 3.70 (s, 3H, OCH_3_). ^13^C NMR (125.8 MHz, DMSO-*d*_6_): *δ* 164.18, 161.11, 160.31, 159.54, 153.89, 147.68, 141.67, 139.53, 134.14, 130.27, 129.67, 129.22, 125.12, 118.72, 118.41, 116.11, 111.44, 109.26, 104.98, 54.95, 52.28, 34.89. HRMS (ESI) *m*/*z* for C_22_H_20_N_5_O_5_^+^ [M + H]^+^, calculated: 434.1459, found: 434.1460. Anal. Calcd. for C_22_H_19_N_5_O_5_ : C, 60.97; H, 4.42; N, 16.16; found: C, 61.12; H, 4.54; N, 16.31%.

##### 4.2.6.7 *N*-((1-(2-((4-methoxyphenyl)amino)-2-oxoethyl)-1*H*-1,2,3-triazol-4-yl)methyl)-2-oxo-2*H*-chromene-3-carboxamide (12g)

Milky solid, yield: 87%, m.p. 201–204 °C. ^1^H NMR (500.1 MHz, DMSO-*d*_6_): *δ* 10.28 (s, 1H, NH-amide), 9.14 (br. s, 1H, CH_2_NH), 8.90 (s, 1H, CH), 8.07 (s, 1H, CH), 7.99 (d, *J* = 6.9 Hz, 1H, CH), 7.76 (t, *J* = 6.7 Hz, 1H, CH), 7.54–7.40 (m, 4H, 4CH), 6.90 (d, *J* = 7.9 Hz, 2H, 2CH), 5.28 (s, 2H, CH_2_), 4.64 (br. s, 2H, CH_2_NH), 3.72 (s, 3H, OCH_3_). ^13^C NMR (125.8 MHz, DMSO-*d*_6_): *δ* 164.10, 161.58, 160.82, 155.97, 154.37, 148.18, 141.81, 134.64, 131.96, 130.78, 129.23, 125.62, 121.20, 119.21, 118.91, 116.62, 114.46, 55.61, 52.62, 35.37. HRMS (ESI) *m*/*z* for C_22_H_20_N_5_O_5_^+^ [M + H]^+^, calculated: 434.1459, found: 434.1464. Anal. Calcd. for C_22_H_19_N_5_O_5_ : C, 60.97; H, 4.42; N, 16.16; found: C, 61.08; H, 4.61; N, 16.27%.

##### 4.2.6.8 2-Oxo-*N*-((1-(2-oxo-2-((3,4,5-trimethoxyphenyl)amino)ethyl)-1*H*-1,2,3-triazol-4-yl)methyl)-2*H*-chromene-3-carboxamide (12h)

Pale yellow solid, yield 80%, m.p. 221–224 °C. FT/IR (*ν*_max_/cm^−1^): 3350 and 3259 (2NH), 1703 (C=O, coumarin), 1656 and 1650 (C=O, amide), 1567, 1507, 1449, 1330, 1301, 1232, 1183, 1125, 1051, 998, 969, 829, 796, 710, 656. ^1^H NMR (400.1 MHz, DMSO-*d*_6_): *δ* 10.45 (s, 1H, NH-amide), 9.19 (br. s, 1H, CH_2_NH), 8.92 (s, 1H, CH), 8.10 (s, 1H, CH), 8.01 (d, *J* = 7.3 Hz, 1H, CH), 7.76 (d, *J* = 7.4 Hz, 1H, CH), 7.52 (d, *J* = 8.1 Hz, 1H, CH), 7.45 (t, *J* = 7.2 Hz, 1H, CH), 6.95 (s, 2H, 2CH), 5.30 (s, 2H, CH_2_), 4.63 (br. s, 2H, CH_2_NH), 3.72 (s, 6H, 2OCH_3_), 3.61 (s, 3H, OCH_3_). ^13^C NMR (100.1 MHz, DMSO-*d*_6_): *δ* 164.52, 161.57, 160.84, 154.37, 153.23, 148.21, 141.25, 134.98, 134.66, 134.04, 130.79, 129.23, 125.63, 119.18, 118.91, 116.63, 97.24, 60.55, 56.10, 52.68, 35.38. HRMS (ESI) *m*/*z* for C_24_H_24_N_5_O_7_^+^ [M + H]^+^, calculated: 494.1670, found: 494.1673. Anal. Calcd. for C_24_H_23_N_5_O_7_ : C, 58.41; H, 4.70; N, 14.19; found: C, 58.49; H, 4.81; N, 14.37%.

##### 4.2.6.9 *N*-((1-(2-((2-fluorophenyl)amino)-2-oxoethyl)-1*H*-1,2,3-triazol-4-yl)methyl)-2-oxo-2*H*-chromene-3-carboxamide (12i)

Milky solid, yield 75%, m.p. 173–176 °C. ^1^H NMR (400.1 MHz, DMSO-*d*_6_): *δ* 10.32 (s, 1H, NH-amide), 9.18 (br. s, 1H, CH_2_NH), 8.92 (s, 1H, CH), 8.08 (s, 1H, CH), 8.01 (d, *J* = 6.5 Hz, 1H, CH), 7.97–7.85 (m, 1H, CH), 7.76 (t, *J* = 5.7 Hz, 1H, CH), 7.52 (d, *J* = 7.6 Hz, 1H, CH), 7.45 (t, *J* = 7.2 Hz, 1H, 1CH), 7.30 (t, *J* = 6.6 Hz, 1H, 1CH), 7.23–7.11 (m, 2H, 2CH), 5.42 (s, 2H, CH_2_), 4.63 (br. s, 2H, CH_2_NH). ^13^C NMR (100.1 MHz, DMSO-*d*_6_): *δ* 165.32, 161.57, 160.82, 154.37, 153.87 (d, ^1^*J*_C–F_ = 244.7 Hz), 148.19, 141.74, 134.65, 130.79, 129.83, 129.28, 128.74, 126.07 (d, ^2^*J*_C–F_ = 18.9 Hz), 125.62, 124.98 (d, ^3^*J*_C–F_ = 3.1 Hz), 124.17, 119.20, 118.91, 116.63, 116.36 (d, ^2^*J*_C–F_ = 19.3 Hz), 52.42, 35.36. HRMS (ESI) *m*/*z* for C_21_H_17_FN_5_O_4_^+^ [M + H]^+^, calculated: 422.1259, found: 422.1263. Anal. Calcd. for C_21_H_16_FN_5_O_4_ : C, 59.86; H, 3.83; N, 16.62; found: C, 59.97; H, 3.91; N, 16.69%.

##### 4.2.6.10 *N*-((1-(2-((4-fluorophenyl)amino)-2-oxoethyl)-1*H*-1,2,3-triazol-4-yl)methyl)-2-oxo-2*H*-chromene-3-carboxamide (12j)

Pale yellow solid, yield 68%, m.p. 224–227 °C. ^1^H NMR (400.1 MHz, DMSO-*d*_6_): *δ* 10.53 (s, 1H, NH-amide), 9.16 (br. s, 1H, CH_2_N*H*), 8.88 (s, 1H, CH), 8.11 (s, 1H, CH), 7.98–6.91 (m, 7H, 7CH), 5.34 (s, 2H, CH_2_), 4.63 (br. s, 2H, CH_2_NH). ^13^C NMR (100.1 MHz, DMSO-*d*_6_): *δ* 164.04, 161.14, 160.29, 158.21 (d, ^1^*J*_C_–_F_ = 240.5 Hz), 153.88, 147.70, 141.90, 134.74, 134.14, 130.27, 129.32, 125.11, 121.02 (d, ^3^*J*_C_–_F_ = 7.5 Hz), 118.67, 118.38, 116.10, 115.45 (d, ^2^*J*_C_–_F_ = 22.4 Hz), 52.24, 34.87. HRMS (ESI) *m*/*z* for C_21_H_17_FN_5_O_4_^+^ [M + H]^+^, calculated: 422.1259, found: 422.1262. Anal. Calcd. for C_21_H_16_FN_5_O_4_ : C, 59.86; H, 3.83; N, 16.62; found: C, 59.94; H, 3.99; N, 16.75%.

##### 4.2.6.11 *N*-((1-(2-((2-chlorophenyl)amino)-2-oxoethyl)-1*H*-1,2,3-triazol-4-yl)methyl)-2-oxo-2*H*-chromene-3-carboxamide (12k)

Milky solid, yield 70%, m.p. 179–181 °C. ^1^H NMR (400.1 MHz, DMSO-*d*_6_): *δ* 10.09 (s, 1H, NH-amide), 9.17 (t, *J* = 5.4 Hz, 1H, CH_2_NH), 8.92 (s, 1H, CH), 8.08 (s, 1H, CH), 8.00 (d, *J* = 7.6 Hz, 1H, CH), 7.80–7.70 (m, 2H, 2CH), 7.57–7.49 (m, 2H, 2CH), 7.45 (t, *J* = 7.5 Hz, 1H, CH), 7.34 (t, *J* = 7.7 Hz, 1H, CH), 7.22 (t, *J* = 7.4 Hz, 1H, CH), 5.44 (s, 2H, CH_2_), 4.63 (d, *J* = 5.4 Hz, 2H, CH_2_NH). ^13^C NMR (100.1 MHz, DMSO-*d*_6_): *δ* 165.39, 161.57, 160.82, 154.37, 148.19, 141.71, 134.65, 134.60, 130.78, 130.08, 129.27, 128.02, 127.18, 126.72, 126.33, 125.63, 119.19, 118.90, 116.62, 52.36, 35.34. HRMS (ESI) *m*/*z* for C_21_H_17_ClN_5_O_4_ [M + H]^+^, calculated: 438.0964, found: 438.0964. Anal. Calcd. for C_21_H_16_ClN_5_O_4_ : C, 57.61; H, 3.68; N, 16.00; found: C, 57.66; H, 3.74; N, 16.09%.

##### 4.2.6.12 *N*-((1-(2-((3-chlorophenyl)amino)-2-oxoethyl)-1*H*-1,2,3-triazol-4-yl)methyl)-2-oxo-2*H*-chromene-3-carboxamide (12l)

Pale yellow solid, yield 82%, m.p. 190–193 °C. ^1^H NMR (500.1 MHz, DMSO-*d*_6_): *δ* 10.47 (s, 1H, NH-amide), 9.15 (br. s, 1H, CH_2_N*H*), 8.89 (s, 1H, CH), 8.10 (s, 1H, CH), 7.98 (d, *J* = 6.5 Hz, 1H, CH), 7.80–7.68 (m, 2H, 2CH), 7.49 (d, *J* = 7.9 Hz, 1H, CH), 7.46–7.38 (m, 2H, 2CH), 7.34 (t, *J* = 6.5 Hz, 1H, CH), 7.12 (d, *J* = 7.0 Hz, 1H, CH), 5.34 (s, 2H, CH_2_), 4.63 (br. s, 2H, CH_2_NH). ^13^C NMR (125.8 MHz, DMSO-*d*_6_): *δ* 165.03, 161.55, 160.74, 154.32, 148.13, 141.89, 140.21, 134.57, 133.58, 131.01, 130.70, 129.24, 125.54, 123.91, 119.16, 119.13, 118.83, 118.06, 116.54, 52.69, 35.33. HRMS (ESI) *m*/*z* for C_21_H_17_ClN_5_O_4_ [M + H]^+^, calculated: 438.0964, found: 438.0967. Anal. Calcd. for C_21_H_16_ClN_5_O_4_ : C, 57.61; H, 3.68; N, 16.00; found: C, 57.75; H, 3.81; N, 16.12%.

##### 4.2.6.13 *N*-((1-(2-((4-chlorophenyl)amino)-2-oxoethyl)-1*H*-1,2,3-triazol-4-yl)methyl)-2-oxo-2*H*-chromene-3-carboxamide (12m)

Milky solid, yield 73%, m.p. 221–223 °C. FT/IR (*ν*_max_/cm^−1^): 3307 and 3228 (2NH), 1702 (C=O, coumarin), 1652 (C=O, amide), 1608, 1567, 1533, 1460, 1455, 1402, 1304, 1245, 1141, 1120, 1092, 1012, 959, 923, 829, 796, 757. ^1^H NMR (500.1 MHz, DMSO-*d*_6_): *δ* 10.62 (s, 1H, NH-amide), 9.18 (br. s, 1H, CH_2_N*H*), 8.91 (s, 1H, CH), 8.11 (s, 1H, CH), 8.00 (d, *J* = 7.1 Hz, 1H, CH), 7.76 (t, *J* = 7.5 Hz, 1H, CH), 7.60 (d, *J* = 8.4 Hz, 2H, 2CH), 7.52 (d, *J* = 8.2 Hz, 1H, CH), 7.45 (t, *J* = 7.3 Hz, 1H, CH), 7.39 (d, *J* = 8.4 Hz, 2H, 2CH), 5.34 (s, 2H, CH_2_), 4.64 (br. s, 2H, C*H*_2_NH). ^13^C NMR (125.8 MHz, DMSO-*d*_6_): *δ* 164.85, 161.60, 160.80, 154.37, 148.19, 141.73, 137.82, 134.65, 130.78, 129.29, 129.23, 127.80, 125.62, 121.23, 119.20, 118.90, 116.62, 52.70, 35.37. HRMS (ESI) *m*/*z* for C_21_H_17_ClN_5_O_4_ [M + H]^+^, calculated: 438.0964, found: 438.0970. Anal. Calcd. for C_21_H_16_ClN_5_O_4_ : C, 57.61; H, 3.68; N, 16.00; found: C, 57.69; H, 3.79; N, 16.09%.

##### 4.2.6.14 *N*-((1-(2-((4-bromophenyl)amino)-2-oxoethyl)-1*H*-1,2,3-triazol-4-yl)methyl)-2-oxo-2*H*-chromene-3-carboxamide (12n)

Yellow-cream solid, yield 71%, m.p.: 230–233 °C. ^1^H NMR (500.1 MHz, DMSO-*d*_6_): *δ* 10.55 (s, 1H, NH-amide), 9.13 (br. s, 1H, CH_2_N*H*), 8.88 (s, 1H, CH), 8.08 (s, 1H, CH), 8.02–7.90 (m, 1H, 1CH), 7.81–7.35 (m, 7H, 7CH), 5.31 (s, 2H, CH_2_), 4.62 (br. s, 2H, C*H*_2_NH). ^13^C NMR (125.8 MHz, DMSO-*d*_6_): *δ* 164.30, 161.07, 160.26, 153.85, 147.64, 141.86, 137.69, 134.10, 131.65, 130.23, 129.30, 125.08, 121.13, 118.69, 118.36, 116.08, 115.35, 52.23, 34.84. HRMS (ESI) *m*/*z* for C_21_H_17_BrN_5_O_4_^+^ [M + H]^+^, calculated: 482.0458, found: 482.0461. Anal. Calcd. for C_21_H_16_BrN_5_O_4_ : C, 52.30; H, 3.34; N, 14.52; found: C, 52.38; H, 3.45; N, 14.61%.

##### 4.2.6.15 2-Oxo-*N*-((1-(2-oxo-2-((4-(trifluoromethyl)phenyl)amino)ethyl)-1*H*-1,2,3-triazol-4-yl)methyl)-2*H*-chromene-3-carboxamide (12o)

Pale yellow solid, yield: 69%, m.p. 221–223 °C. ^1^H NMR (500.1 MHz, DMSO-*d*_6_): *δ* 10.13 (s, 1H, NH-amide), 9.16 (br. s, 1H, CH_2_N*H*), 8.91 (s, 1H, CH), 8.07 (s, 1H, CH), 8.00 (d, *J* = 6.8 Hz, 1H, CH), 7.77 (d, *J* = 5.5 Hz, 2H, 2CH), 7.69 (t, *J* = 6.4 Hz, 1H, CH), 7.57–7.40 (m, 4H, 4CH), 5.39 (s, 2H, CH_2_), 4.63 (d, *J* = 4.7 Hz, 2H, CH_2_NH). ^13^C NMR (125.8 MHz, DMSO-*d*_6_): *δ* 166.01, 161.57, 160.81, 154.38, 148.19, 141.58, 134.87, 134.65, 133.63, 130.78, 129.25, 126.89, 125.62, 124.12, 122.84, 119.20, 118.90, 116.62, 52.16, 35.36. HRMS (ESI) *m*/*z* for C_22_H_17_F_3_N_5_O_4_^+^ [M + H]^+^, calculated: 472.1227, found: 472.1235. Anal. Calcd. for C_22_H_16_F_3_N_5_O_4_ : C, 56.05; H, 3.42; N, 14.86; found: C, 56.14; H, 3.56; N, 14.93%.

##### 4.2.6.16 *N*-((1-(2-((2-cyanophenyl)amino)-2-oxoethyl)-1*H*-1,2,3-triazol-4-yl)methyl)-2-oxo-2*H*-chromene-3-carboxamide (12p)

Pale yellow solid, yield 69%, m.p. 156–158 °C. ^1^H NMR (400.1 MHz, DMSO-*d*_6_): *δ* 10.72 (s, 1H, NH-amide), 9.18 (t, *J* = 4.7 Hz, 1H, CH_2_NH), 8.92 (s, 1H, CH), 8.08 (s, 1H, CH), 8.01 (d, *J* = 7.6 Hz, 1H, CH), 7.85 (d, *J* = 7.6 Hz, 1H, CH), 7.76 (t, *J* = 7.8 Hz, 1H, CH), 7.72–7.66 (m, 2H, 2CH), 7.52 (d, *J* = 8.3 Hz, 1H, CH), 7.45 (t, *J* = 7.4 Hz, 1H, 1CH), 7.38 (t, *J* = 7.2 Hz, 1H, 1CH), 5.45 (s, 2H, CH_2_), 4.64 (d, *J* = 4.7 Hz, 2H, C*H*_2_NH). ^13^C NMR (100.1 MHz, DMSO-*d*_6_): *δ* 165.68, 161.57, 160.82, 154.38, 148.20, 141.80, 139.80, 134.65, 134.46, 133.94, 130.79, 129.26, 126.55, 125.66, 125.62, 119.19, 118.91, 117.09, 116.63, 107.10, 52.27, 35.34. HRMS (ESI) *m*/*z* for C_22_H_17_N_6_O_4_^+^ [M + H]^+^, calculated: 429.1306, found: 429.1309. Anal. Calcd. for C_22_H_16_N_6_O_4_ : C, 61.68; H, 3.76; N, 19.62; found: C, 61.81; H, 3.87; N, 19.71%.

##### 4.2.6.17 *N*-((1-(2-((3-cyanophenyl)amino)-2-oxoethyl)-1*H*-1,2,3-triazol-4-yl)methyl)-2-oxo-2*H*-chromene-3-carboxamide (12q)

Milky solid, yield 77%, m.p. 185–188 °C. FT/IR (*ν*_max_/cm^−1^): 3324 and 3074 (2NH), 2229 (CN), 1702 (C=O, coumarin), 1677 and 1653 (C=O, amide), 1653, 1567, 1526, 1492, 1449, 1365, 1295, 1244, 1162, 1122, 1049, 960, 866, 831, 797, 760, 713. ^1^H NMR (400.1 MHz, DMSO-*d*_6_): *δ* 10.84 (s, 1H, NH-amide), 9.18 (t, *J* = 5.1 Hz, 1H, CH_2_N*H*), 8.93 (s, 1H, CH), 8.08 (s, 1H, CH), 8.05 (s, 1H, CH), 8.02 (d, *J* = 7.7 Hz, 1H, CH), 7.83–7.72 (m, 2H, 2CH), 7.60–7.55 (m, 2H, 2CH), 7.54 (d, *J* = 8.4 Hz, 1H, CH), 7.46 (t, *J* = 7.5 Hz, 1H, 1CH), 5.37 (s, 2H, CH_2_), 4.63 (d, *J* = 5.1 Hz, 2H, CH_2_NH). ^13^C NMR (100.1 MHz, DMSO-*d*_6_): *δ* 165.49, 161.58, 160.84, 154.39, 148.22, 141.77, 139.64, 134.67, 130.95, 130.80, 129.24, 127.85, 125.64, 124.28, 122.34, 119.07, 118.92, 116.64, 112.19, 106.03, 52.85, 35.35. HRMS (ESI) *m*/*z* for C_22_H_17_N_6_O_4_^+^ [M + H]^+^, calculated: 429.1306, found: 429.1312. Anal. Calcd. for C_22_H_16_N_6_O_4_ : C, 61.68; H, 3.76; N, 19.62; found: C, 61.80; H, 3.88; N, 19.70%.

##### 4.2.6.18 *N*-((1-(2-((4-cyanophenyl)amino)-2-oxoethyl)-1*H*-1,2,3-triazol-4-yl)methyl)-2-oxo-2*H*-chromene-3-carboxamide (12r)

Pale yellow solid, yield 85%, m.p. 208–211 °C. FT/IR (*ν*_max_/cm^−1^): 3310 and 3299 (2NH), 2228 (CN), 1693 (C=O, coumarin), 1670 and 1645 (C=O, amide), 1598, 1569, 1534, 1448, 1407, 1308, 1257, 1241, 1230, 1169, 1123, 1092, 1068, 959, 923, 810, 795, 767, 749, 669. ^1^H NMR (400.1 MHz, DMSO-*d*_6_): *δ* 10.92 (s, 1H, NH-amide), 9.18 (br. s, 1H, CH_2_N*H*), 8.92 (s, 1H, CH), 8.09 (s, 1H, CH), 8.01 (d, *J* = 7.5 Hz, 1H, CH), 7.85–7.70 (m, 5H, 5CH), 7.52 (d, *J* = 8.3 Hz, 1H, CH), 7.45 (t, *J* = 7.4 Hz, 1H, 1CH), 5.39 (s, 2H, CH_2_), 4.64 (br. s, 2H, CH_2_NH). ^13^C NMR (100.1 MHz, DMSO-*d*_6_): *δ* 165.66, 161.57, 160.83, 154.37, 148.21, 143.05, 141.85, 134.66, 133.93, 130.79, 129.23, 125.63, 119.73, 119.37, 119.18, 118.90, 116.63, 106.01, 52.73, 35.37. HRMS (ESI) *m*/*z* for C_22_H_17_N_6_O_4_^+^ [M + H]^+^, calculated: 429.1306, found: 429.1306. Anal. Calcd. for C_22_H_16_N_6_O_4_ : C, 61.68; H, 3.76; N, 19.62; found: C, 61.78; H, 3.84; N, 19.77%.

##### 4.2.6.19 *N*-((1-benzyl-1*H*-1,2,3-triazol-4-yl)methyl)-2-oxo-2*H*-chromene-3-carboxamide (13)

Milky solid, yield 78%, m.p. 152–155 °C. ^1^H NMR (400.1 MHz, DMSO-*d*_6_): *δ* 9.17 (br. s, 1H, CH_2_NH), 8.89 (s, 1H, CH), 8.23 (br. s, 1H, CH), 7.99 (d, *J* = 5.0 Hz, 1H, CH), 7.75 (t, *J* = 7.2 Hz, 1H, CH), 7.59–7.20 (m, 7H, 7CH), 5.59 (s, 2H, CH_2_), 4.57 (br. s, 2H, CH_2_NH). ^13^C NMR (100.1 MHz, DMSO-*d*_6_): *δ* 161.59, 160.77, 154.35, 148.14, 143.97, 136.38, 134.64, 130.76, 129.21, 128.62, 128.48, 125.61, 123.44, 119.21, 118.88, 116.61, 53.43, 34.15. HRMS (ESI) *m*/*z* for C_20_H_17_N_4_O_3_^+^ [M + H]^+^, calculated: 361.1295, found: 361.1299. Anal. Calcd. for C_20_H_16_N_4_O_3_ : C, 66.66; H, 4.48; N, 15.55; found: C, 66.78; H, 4.56; N, 15.66%.

##### 4.2.6.20 *N*-((1-(4-chlorobenzyl)-1*H*-1,2,3-triazol-4-yl)methyl)-2-oxo-2*H*-chromene-3-carboxamide (14)

Milky solid, yield 92%, m.p. 179–181 °C. ^1^H NMR (400.1 MHz, DMSO-*d*_6_): *δ* 9.15 (br. s, 1H, CH_2_N*H*), 8.89 (s, 1H, CH), 8.14 (s, 1H, CH), 7.99 (d, *J* = 7.3 Hz, 1H, CH), 7.76 (t, *J* = 7.4 Hz, 1H, CH), 7.51 (d, *J* = 8.1 Hz, 1H, 1CH), 7.49–7.38 (m, 3H, 3CH), 7.34 (d, *J* = 7.8 Hz, 2H, 2CH), 5.60 (s, 2H, CH_2_), 4.59 (br. s, 2H, CH_2_NH). ^13^C NMR (100.1 MHz, DMSO-*d*_6_): *δ* 161.59, 160.77, 154.35, 148.15, 135.46, 134.65, 133.32, 130.77, 130.42, 129.21, 129.16, 125.62, 121.58, 119.19, 118.88, 116.61, 52.50, 35.46. HRMS (ESI) *m*/*z* for C_20_H_16_ClN_4_O_3_^+^ [M + H]^+^, calculated: 395.0905, found: 395.0912. Anal. Calcd. for C_20_H_15_ClN_4_O_3_ : C, 60.84; H, 3.83; N, 14.19; found: C, 60.96; H, 3.99; N, 14.23%.

### 4.3 α-Glucosidase inhibition assay

α-Glucosidase enzyme ((EC3.2.1.20, *Saccharomyces cerevisiae*, 20 U mg^−1^) and substrate (*p*-nitrophenyl glucopyranoside) were purchased from Sigma-Aldrich. α-Glucosidase (20 U mg^−1^) was dissolved in potassium phosphate buffer (50 mM, pH 6.8) to prepare the enzyme stock solution. A working enzyme solution of 1 U mL^−1^ was then prepared from this stock. For the assay, 20 µL of the enzyme working solution was added to a total reaction volume of 200 µL, resulting in a final enzyme activity of 0.02 U per well (equivalent to 0.1 U mL^−1^ in the reaction mixture). Coumarin-1,2,3-triazole hybrids 12a–12r, 13, and 14 were dissolved in DMSO. (A final DMSO concentration of 10% was selected based on preliminary tolerance studies showing no effect on α-glucosidase activity (Fig. S1)). The various concentrations of these compounds (20 mL), enzyme solution (20 mL), and potassium phosphate buffer (135 mL) were added to the 96-well plate and incubated at 37 °C for 10 min. Afterwards, the substrate (25 mL, 4 mM) was added to the mentioned mixture and allowed to incubate at 37 °C for 20 min. Finally, the change in absorbance was measured at 405 nm by spectrophotometer (Gen5, Power wave xs2, BioTek, America). The percentage of enzyme inhibition was calculated using eqn (1) and IC_50_ values were obtained from non-linear regression curve using the Logit method.^59^

6 % Inhibition = [(Abs_control_ − Abs_sample_)/Abs_control_] × 100

### 4.4 Computational studies

#### 4.4.1 Homology modeling

The amino acid sequence of *Saccharomyces cerevisiae α*-glucosidase (UniProt ID: P53341) was extracted from UNIPROT database (https://www.uniprot.org). Subsequently, a comprehensive sequence similarity search for this sequence in the Protein Data Bank (PDB) was performed using PSI-BLAST to identify suitable structural templates. Candidate templates were evaluated based on multiple criteria, including sequence identity, resolution, and the presence of the co-crystallized ligands within the binding site. Among these, the structure with PDB ID 3 A4A emerged as the most favorable template due to its high sequence identity (72%), excellent resolution, and the presence of a natural ligand in its active site. Therefore, this structure was selected as the template. Finally, the homology model was constructed using the SWISS-MODEL workspace (https://swissmodel.expasy.org/).

#### 4.4.2 Molecular docking study

Molecular docking studies were performed for all synthesized compounds 12a–12r, 13, and 14 using AutoDock4. The homology-modeled protein was prepared as the receptor by assigning Kollman charges and removing non-polar hydrogen atoms using AutoDockTools. The ligand structures were energy-minimized in Chem3D 16.0 and subsequently prepared with AutoDockTools by assigning Gasteiger charges and AutoDock4 atom types (AD4 types), along with the removal of nonpolar hydrogen atoms.

For each ligand, 100 independent genetic algorithm runs with default settings (population size = 150, maximum number of generations = 27 000, rate of crossover = 0.8, mutation rate of 0.02) were carried out within the predefined binding site of the protein, determined during homology model validation (grid box dimensions = 50 × 50 × 50 Å, center coordinates: *x* = 21.544, *y* = −7.476, *z* = 24.158, spacing = 0.375 Å). Docking poses were clustered based on RMSD values (Maximum RMS tolerance for conformational cluster analysis = 2.00 Å) and protein–ligand interactions were visualized and analyzed using Discovery Studio Visualizer v21.1.0.20298 and Open-source PyMOL (The PyMOL Molecular Graphics System, Version 3.2.0; Schrödinger, LLC).

#### 4.4.3 Molecular dynamics study

The molecular dynamics (MD) study of α-glucosidase in complex with the most potent compound 12q was investigated using the GROMACS 2025.4 on an Ubuntu 2024 system, employing the AMBER99SB force field. Trajectory processing was carried out to correct the periodicity and align frames to the protein backbone prior to analysis. RMSD and RMSF were computed on the backbone and Cα atoms. Hydrogen bonds and π–π interactions were distinguished using standard distance/angle cutoffs.

Following the molecular docking studies, ligand was prepared using the ACPYPE package. The protein–ligand complex was then processed using the Gromacs preprocessor, followed by manual indexing. The complex was centered in a dodecahedron box, 17 Na^+^ ions were added to the system to make the overall charge of the system neutral. Afterwards, the system was solvated by adding explicit TIP3P water molecules to the box. Energy minimization was performed with a maximum number of 50 000 cycles using steepest descent algorithm to fix probable clashes. System equilibration was carried out in two stages:

(1) NVT Ensemble: A 200 ps simulation at 300 K using the velocity rescale thermostat.

(2) NPT Ensemble: A 400 ps simulation using the Parrinello-Rahman barostat and the leap-frog integrator.

After reaching stabilization, MD simulation was conducted once with a production time of 200 ns on an RTX 4090 GPU, and the resulting trajectories were analyzed using the MDAnalysis Python package.^60,61^

### 4.5 α-Glucosidase kinetic studies

The kinetic analysis was performed for the most potent substituted coumarin 12q to reveal the inhibition mode against α-glucosidase. The 20 mL of enzyme solution (1U mL^−1^) was incubated with different concentrations (0, 250, 500, 1000, and 2000 nM) of this compound for 15 min at 30 °C. Afterwards, various concentrations of substrate (*p*-nitrophenyl glucopyranoside, 1 to 16 mM) were added to measure the change of absorbance for 20 min at 405 nm by spectrophotometer (Gen5, Power wave xs2, BioTek, America).

In the presence of a competitive inhibitor, *K*_m_ increases while *V*_max_ does not change. Michaelis–Menten saturation curve for an enzyme reaction shows the relation between the substrate concentration and reaction rate as bellow:7
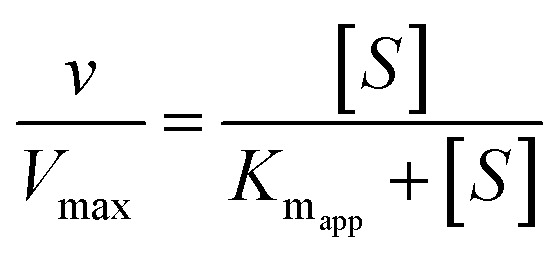


According to Michaelis–Menten graph, *K*_m_app__ is also defined as:8
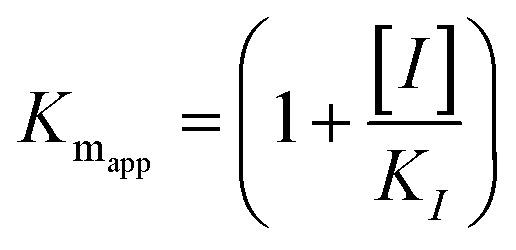


[*I*] is the concentration of inhibitor.

Lineweaver Burk plot that provides a useful graphical method for analysis of the Michaelis–Menten is represented as:9
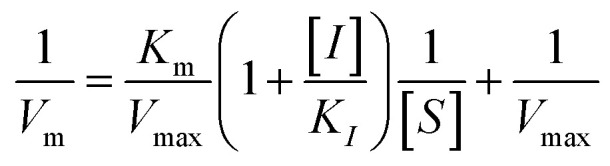


Therefore, the slope of Lineweaver Burk plot is equal to:10
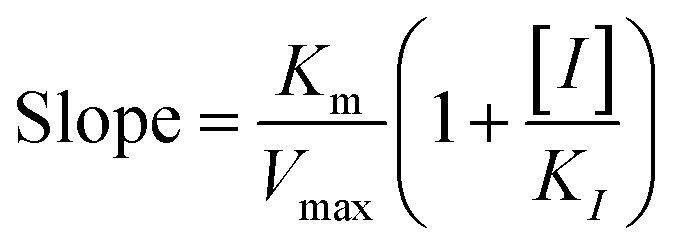


The *K*_m_app__ value is calculated by eqn (10):11
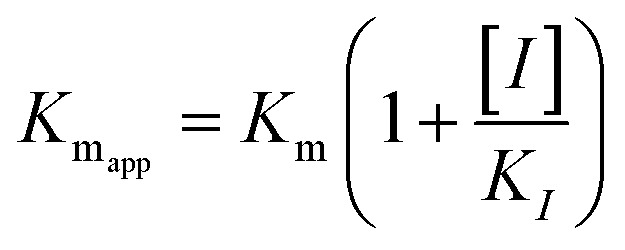


Therefore, from replot of *K*_m_app__*vs.* [*I*], eqn (11) can be used for the calculation of KI:^62,63^12
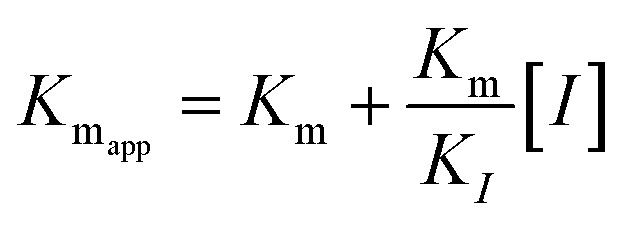


### 4.6 α-Amylase inhibition assay

To investigate the α-amylase inhibitory potency, a bacterial α-amylase was obtained from Merck (Art. 1329, 130 U mg^−1^). 40 µl of the compound 12q (dissolved in DMSO, with a final concentration of 100 µM) and 40 µl of α-amylase solution (0.5 mg mL^−1^ in sodium phosphate buffer, 0.006 M, pH 6.9, containing 0.02 M sodium chloride) were added to appropriate tubes and incubated at 25 °C for 10 minutes. Subsequently, 40 µl of a starch solution (1% in 0.02 M sodium phosphate buffer) was added to each tube at 5-seconds intervals, followed by an additional 10-minutes incubation at 25 °C. The reactions were then terminated by adding 100 µl of dinitrosalicylic acid (DNS) reagent, and the tubes were placed in a boiling water bath for 5 minutes before cooling to room temperature. Acarbose was tested using the same procedure. Afterward, the reaction mixtures were diluted with 900 µl of distilled water, and absorbance was recorded at 540 nm (46) A dose–response experiment for α-amylase inhibition was performed over a concentration range of 0–800 µM (Fig. S2). The final enzyme concentration in the reaction mixture was 2.5 U mL^−1^. At inhibitor concentrations above 300 µM, precipitation occurred in the reaction; therefore, samples were centrifuged prior to absorbance measurement. In this assay, acarbose served as the positive control and showed an IC50 of approximately 100 µM under our experimental conditions. Accordingly, 100 µM was selected as the reference concentration and reported in the manuscript for comparison.

### 4.7 Fluorescence spectroscopy measurements and evaluation of inner-filter effect

Intrinsic fluorescence spectra of α-glucosidase were recorded in the absence and presence of increasing concentrations of compound 12q at 25 °C. The enzyme samples were excited at 280 nm, and emission spectra were collected between 300 and 600 nm.

Because compound 12q itself exhibits intrinsic fluorescence within the 300–450 nm range, which partially overlaps with the enzyme's emission region, spectra of the compound alone were recorded under identical experimental conditions and subtracted as blanks to isolate the enzyme's true fluorescence signal.

Because fluorescence measurements may be affected by the inner-filter effect (IFE) caused by absorption of excitation or emission light by the ligand, the absorbance of each assay mixture was carefully evaluated at the excitation wavelength (280 nm) and at the emission maximum (∼340 nm). In general, fluorescence intensities can be corrected using the eqn (1).

Where *F*_corr_ and *F*_obs_ represent the corrected and observed fluorescence intensities, respectively, and Aex and Aem correspond to the absorbance values at the excitation and emission wavelengths. In the present study, the absorbance values at both wavelengths were consistently below 0.1 for all mixtures. Since absorbance values in this range produce negligible IFE (<5% error), no numerical correction using the above equation was required. Consequently, the observed, blank-subtracted fluorescence intensities were used directly for subsequent analysis.

This assay was carried out for the most potent derivative 12q to measure the fluorescence intensity. To this aim, different solutions containing different concentrations (0 to 1.0 µM) of the inhibitor and α-glucosidase (3 mL, 0.1 U mL^−1^) were held for 10 min to equilibrate before measurements. Moreover, the fluorescence of the buffer containing compound 12q in the absence of the enzyme was subtracted as the background fluorescence. Afterwards, at the excitation wavelength of 280 nm, the fluorescence emission spectra were measured from 300 to 450 nm using a Synergy HTX multi-mode reader (Biotek Instruments, Winooski, VT, USA) equipped with a 1.0 cm quartz cell holder.^64^

### 4.8 CD analysis

The CD spectra were collected using a Jasco J-810 spectropolarimeter. Measurements were performed over the wavelength range of 260–190 nm with a 0.5 nm data pitch, 1 nm bandwidth, 4-seconds response time, and a scan speed of 100 nm min^−1^. A single accumulation was recorded. All spectra were obtained at room temperature using a 0.1-cm pathlength quartz cuvette.

The enzyme sample was analyzed at a concentration of 0.05 mg mL^−1^. To reduce high-wavelength noise and minimize solvent background, the sample was prepared in water without buffer, containing only a minimal amount of DMSO, as in the reference sample Yang.jwr. The baseline (reference) spectrum was collected under the same solvent conditions. Secondary-structure estimation was performed using the CDNN software package, which applies CONTIN-based deconvolution algorithms.

### 4.9 *In vivo* antidiabetic study

Male specific pathogen-free (SPF) grade C57BL/6J mice (6–8 weeks old with initial body weight (BW) 20–25 g; obtained from Production and Research Complex Pasteur Institute of Iran) were used in present study. They were housed in Experimental Animal Laboratory, School of Pharmacy, Tehran University of Medical Sciences, Tehran, Iran in ventilated cages under controlled environmental conditions (12 h light/dark cycle, 22 ± 2 °C, and 50 ± 10% relative humidity) and were allowed free access to food and water throughout the experiment. On the final day of the study, the mice were anesthetized *via* intraperitoneal injection of ketamine/xylazine (50 mg kg^−1^ and 5 mg kg^−1^, respectively) and subsequently euthanized. All experimental procedures adhered to ethical guidelines approved by the institutional ethics committee (Approval No.: IR.TUMS.BLC.1404.194).

### 4.10 Acute oral toxicity study

The acute oral toxicity of the compound was determined using the oral ATC method as described in OECD guideline for testing of chemicals.^65^ To this aim, C57BL/6J mice were classified into five groups of three, administrating by our compound in doses of 5, 50, 300, 500, and 2000 mg kg^−1^ BW. Subsequently, animals were carefully monitored over a period of two weeks for any signs of toxicity and mortality. No mortality or signs of clinical toxicity were observed at doses of 5, 50, and 300 mg kg^−1^ BW, while all animals expired at higher doses within 12 h. Therefore, the No Observed Adverse Effect Level (NOAEL) was 300 mg kg^−1^ BW, and the Lowest Observed Adverse Effect Level (LOAEL) was determined to be 500 mg kg^−1^ BW.

### 4.11 Induction of diabetes

Adult male C57BL/6J mice were randomly divided into six groups; the first group was normal control, and the other groups were followed by diabetes induction using a high-fat diet (HFD) and streptozotocin (STZ). To this aim, animals in groups 2 to 6 received an HFD for six weeks and received intraperitoneal injections of STZ (30 mg kg^−1^) dissolved in freshly-prepared cold citrate buffer (0.1 M, pH 4.5) for five consecutive days. Subsequently, their fasting blood glucose levels were evaluated using a glucometer (Accu-Chek, Roche). Animals with glucose levels below 200 mg dL^−1^ were excluded, and the rest were considered diabetic and selected for further experimentation. Because the HFD + STZ model does not produce a type 2 diabetes phenotype in female C57BL/6J mice,^66^ our evaluation of compound 12q was restricted to male animals– a limitation we acknowledge here and that is to be addressed in future studies involving this scaffold.

Mice were maintained on HFD and had free access to water throughout the treatment period, and were fasted before measuring the blood glucose for 6 h. Data were analyzed using Sigma Plot. No formal randomization method was used; the cages were assigned to groups by cage order. The individual performing blood collection was blinded to group allocation.

### 4.12 Experimental design

Animals were randomly divided into six groups, each consisting of eight animals (*n* = 8):

• Group 1: normal healthy mice (non-diabetic control)

• Group 2: diabetic mice without treatment (diabetic control)

• Group 3: diabetic mice treated orally with compound 12q at 15 mg kg per day.

• Group 4: diabetic mice treated orally with compound 12q at 10 mg kg per day.

• Group 5: diabetic mice treated orally with compound 12q at 5 mg kg per day.

• Group 6: diabetic mice treated orally with acarbose at 25 mg kg per day (positive control)

All treatments were administered orally *via* gavage daily. Fasting blood glucose levels were measured weekly by collecting fresh blood from the tail veins of the mice to evaluate the hyperglycemic effect of the compound 12q in comparison with acarbose.

### 4.13 Oral sucrose tolerance test (OSTT)

To assess the effect of compound 12q on postprandial glucose levels, an oral sucrose tolerance test (OSTT) using previously published methods with minor alterations was performed on day 28 of the study. After an overnight fast, FBG levels were measured, and mice from each group received their respective treatments orally *via* gavage. Following 10 minutes, a sucrose solution (2 g kg^−1^ BW) was administered orally. Blood samples were collected from the tail vein at 0, 15, 30, 60, 90, and 120 minutes following sucrose administration. The blood glucose levels were measured using a glucometer to assess glucose tolerance and to compare the anti-hyperglycemic potential of compound 12q with that of the standard drug (acarbose).^67^

## Ethical statement

All animal procedures were performed in accordance with the Guidelines for Care and Use of Laboratory Animals of Tehran University of Medical Sciences and approved by the institutional ethics committee of TUMS (Approval No.: IR.TUMS.BLC.1404.194).

## Author contributions

Loghman Firoozpour and Alireza Foroumadi: conceptualization, project administration, resources, and supervision; Mahdis Sadeghi Moghadam, Bahareh Bayati, and Fariba Peytam: data curation, formal analysis, investigation, and writing – original draft; Maryam Norouzbahari, Hayrettin Ozan Gulcan, Somayeh Mojtabavi, and Vahid Sheibani: data curation and formal analysis; Mahdis Sadeghi Moghadam, Bahareh Bayati, and Fahimeh Ghasemi: software and visualization; Seyed Esmaeil Sadat-Ebrahimi and Maliheh Barazandeh Tehrani: validation and writing – review & editing.

## Conflicts of interest

The authors declare that they have no conflicts of interest.

## Supplementary Material

RA-016-D6RA01115B-s001

## Data Availability

The authors confirm that the data supporting the finding of this study are available within the manuscript and supplementary information (SI). Supplementary information is available. See DOI: https://doi.org/10.1039/d6ra01115b.
