# Supplementary material for: Potent and selective α-glucosidase inhibition by coumarin–triazole conjugates: design, in vivo evaluation, and computational insights
Source: RSC Adv. 2026 Jun 1;16(32):29844–66. doi: 10.1039/d6ra01115b (PMC13227517; doi:10.1039/d6ra01115b)
Supplement: RA-016-D6RA01115B-s001 [file RA-016-D6RA01115B-s001.pdf]

Supplementary Information (SI) for RSC Advances.
This journal is © The Royal Society of Chemistry 2026

Potent and Selective *α*-Glucosidase Inhibition by Coumarin–Triazole Conjugates: Design, *In Vivo* Evaluation, and Computational Insights

Mahdis Sadeghi Moghadam [1], Bahareh Bayati [2], Fariba Peytam [1,2], Maryam Norouzbahari [3], Hayrettin Ozan Gülcan [4], Somayeh Mojtabavi [5], Fahimeh Ghasemi [6], Seyed Esmaeil Sadat-Ebrahimi [1], Maliheh Barazandeh Tehrani [1], Vahid Sheibani [7], Loghman Firoozpour [1,2,*], Alireza Foroumadi [1,2,*]

[1] Department of Medicinal Chemistry, Faculty of Pharmacy, Tehran University of Medical Sciences, Tehran, Iran

[2] Drug Design and Development Research Center, The Institute of Pharmaceutical Sciences (TIPS), Tehran University of Medical Sciences, Tehran, Iran

[3] Faculty of Pharmacy, Final International University, Catalkoy, Kyrenia via Mersin 10 Turkey

[4] Faculty of Pharmacy, Eastern Mediterranean University, Famagusta, TRNC, via Mersin 10 Turkey

[5] Department of Pharmaceutical Biotechnology, Faculty of Pharmacy, Tehran University of Medical Sciences, Tehran, Iran

[6] Department of Medical Biotechnology, School of Advanced Technologies in Medicine, Tehran University of Medical Sciences, Tehran, Iran

[7] Neuroscience Research Center, Institute of Neuropharmacology, Kerman University of Medical Sciences, Kerman, Iran

*** Corresponding authors:** Dr. Loghman Firoozpour (firoozpour@gmail.com), Dr. Alireza Foroumadi (aforoumadi@yahoo.com)

^{1}H NMR spectrum of 2-oxo-N-((1-(2-oxo-2-(phenylamino)ethyl)-1H-1,2,3-triazol-4-yl)methyl)-2H-chromene-3-carboxamide (**12a**)

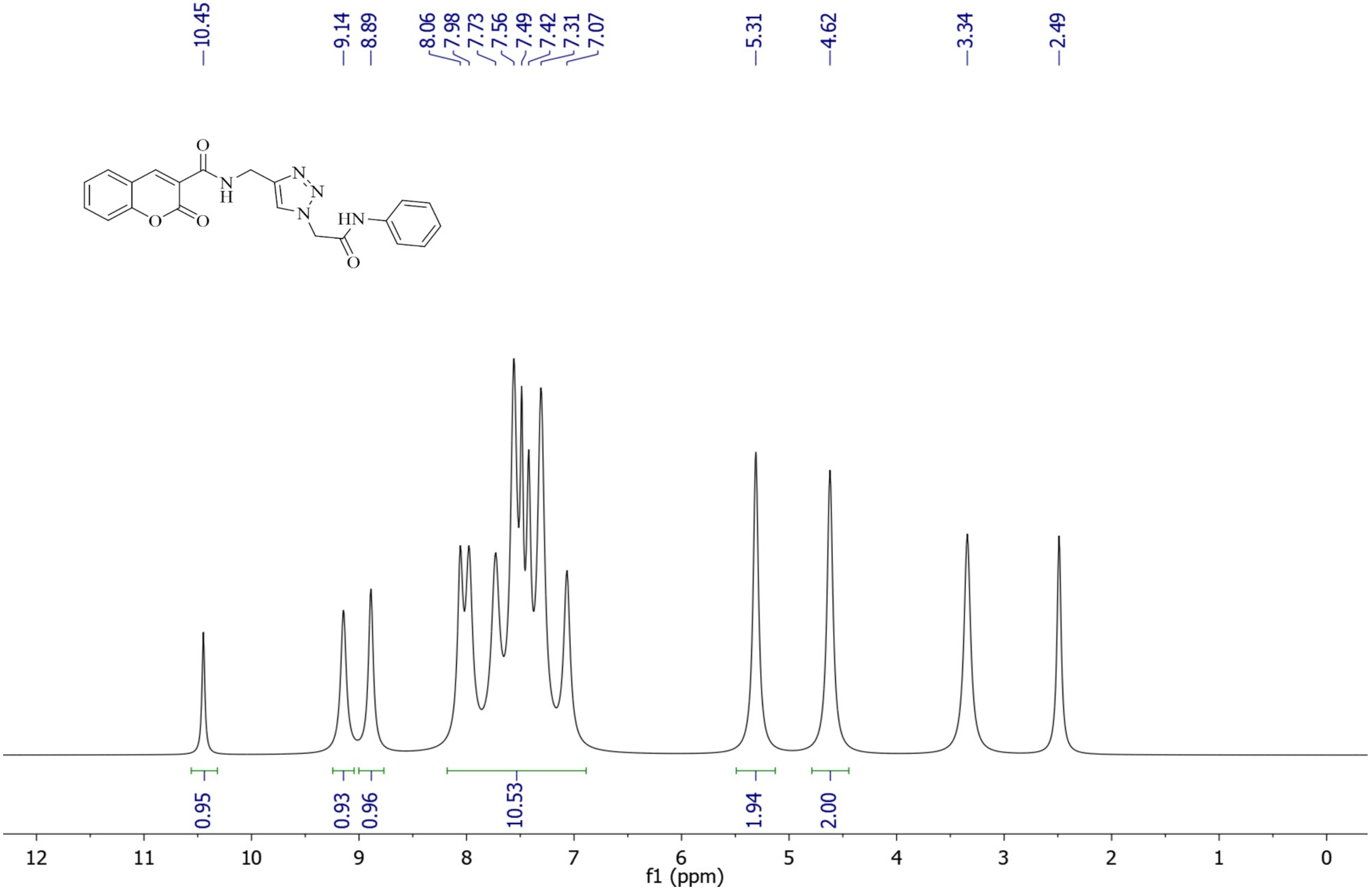

^{13}C NMR spectrum of 2-oxo-N-((1-(2-oxo-2-(phenylamino)ethyl)-1H-1,2,3-triazol-4-yl)methyl)-2H-chromene-3-carboxamide (**12a**)

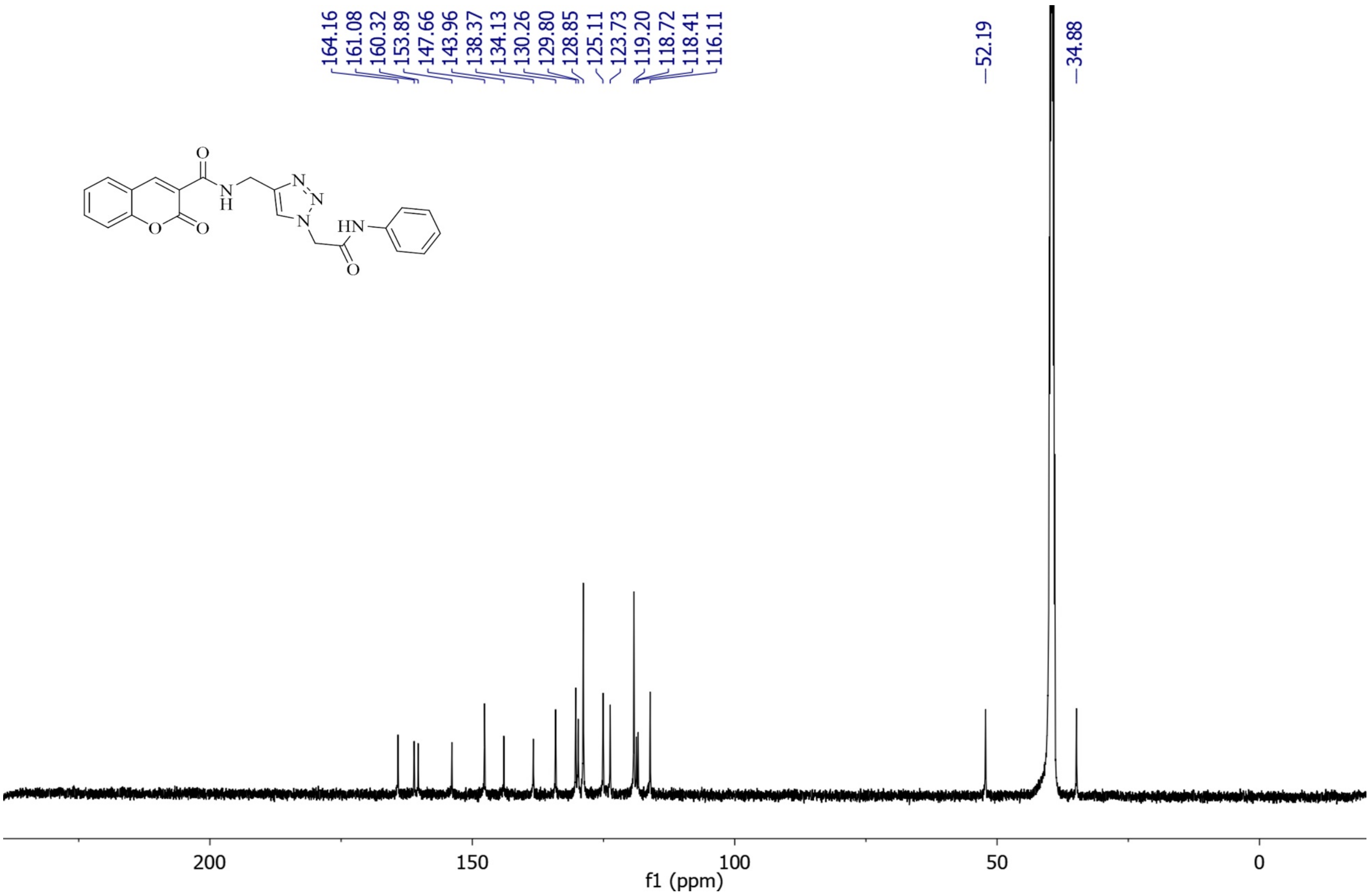

^{1}H NMR spectrum of 2-oxo-N-((1-(2-oxo-2-(o-tolylamino)ethyl)-1H-1,2,3-triazol-4-yl)methyl)-2H-chromene-3-carboxamide (**12b**)

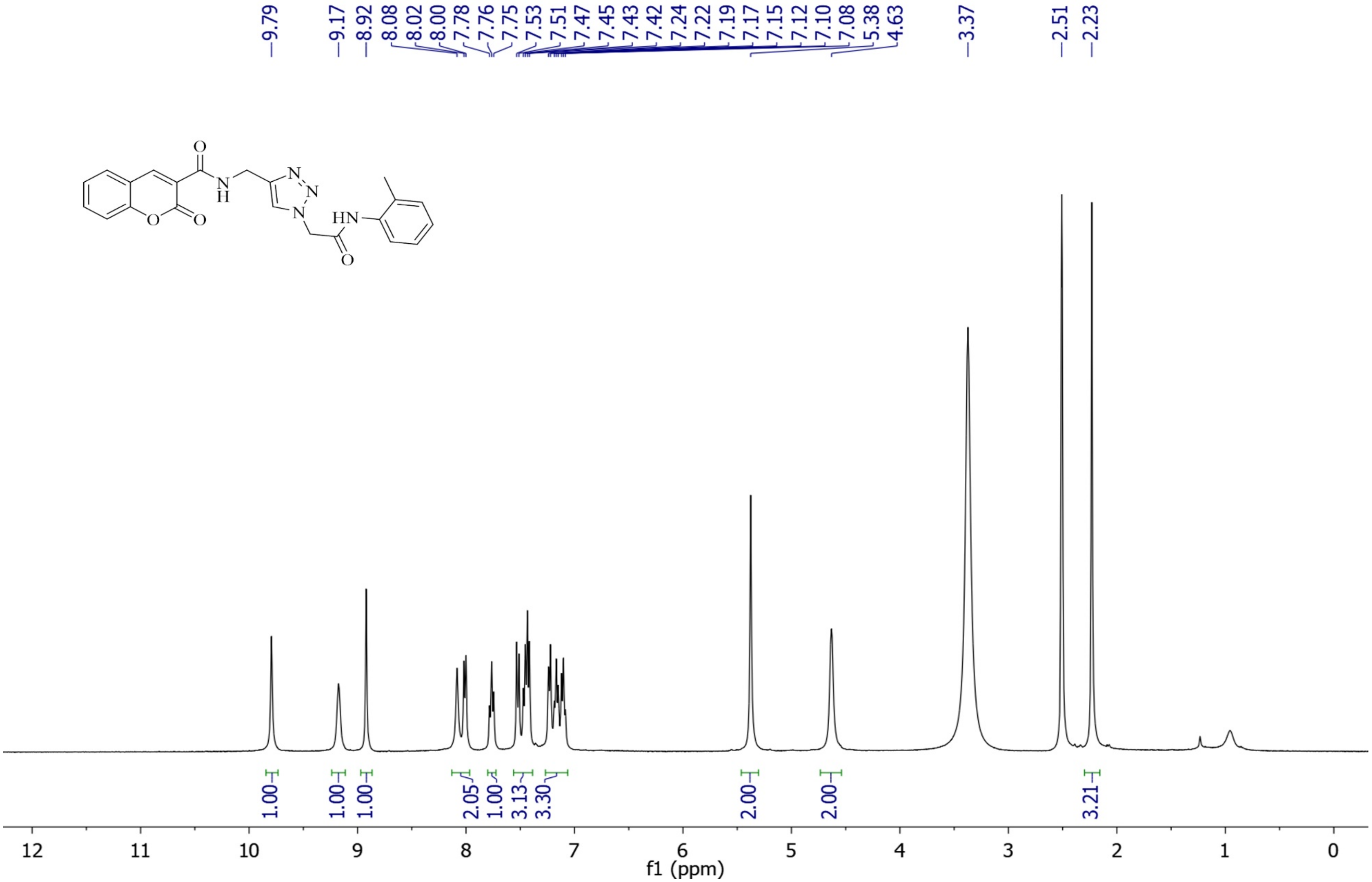

^{13}C NMR spectrum of 2-oxo-N-((1-(2-oxo-2-(o-tolylamino)ethyl)-1H-1,2,3-triazol-4-yl)methyl)-2H-chromene-3-carboxamide (**12b**)

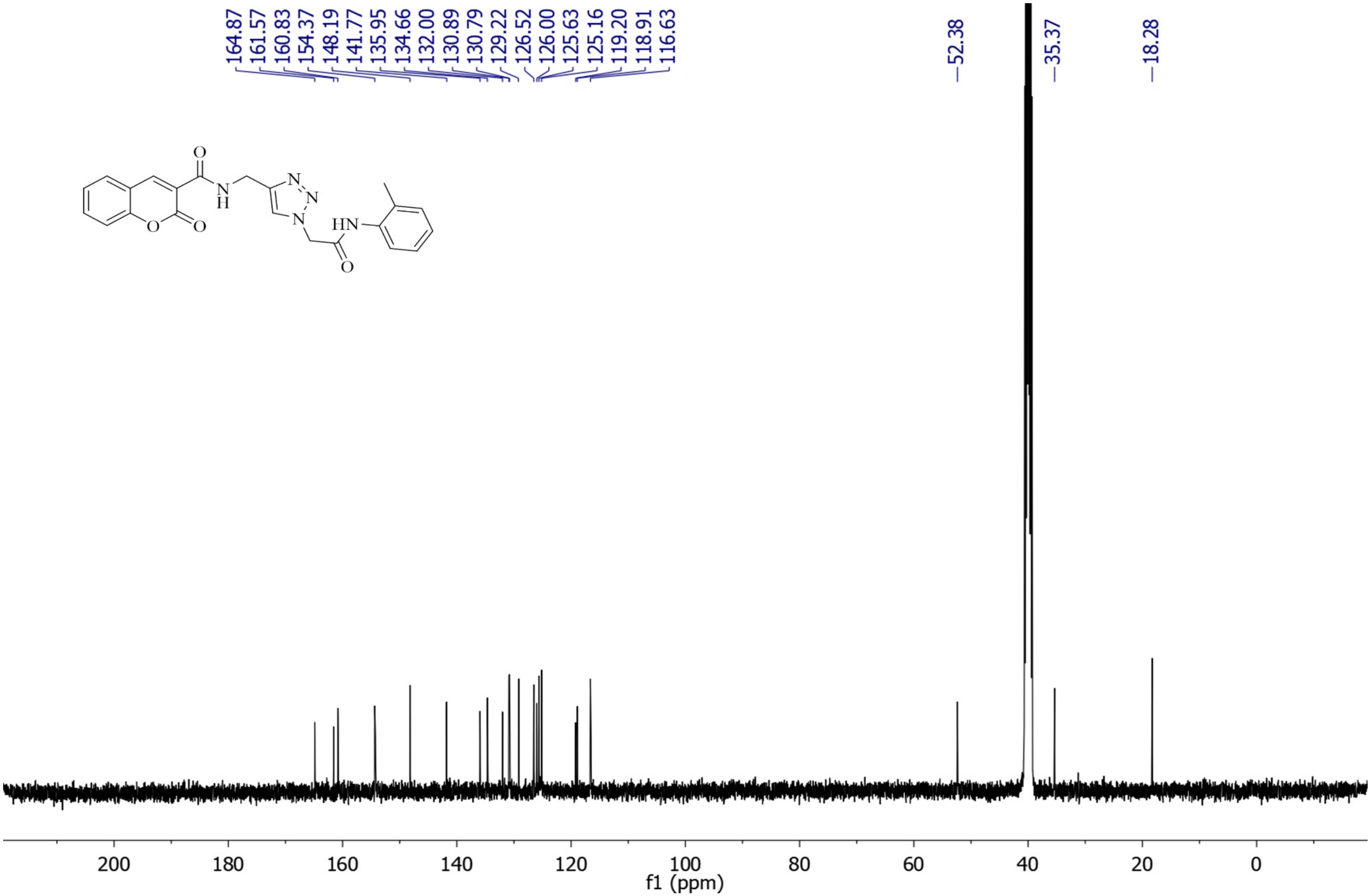

^{1}H NMR spectrum of 2-oxo-N-((1-(2-oxo-2-(m-tolylamino)ethyl)-1H-1,2,3-triazol-4-yl)methyl)-2H-chromene-3-carboxamide (**12c**)

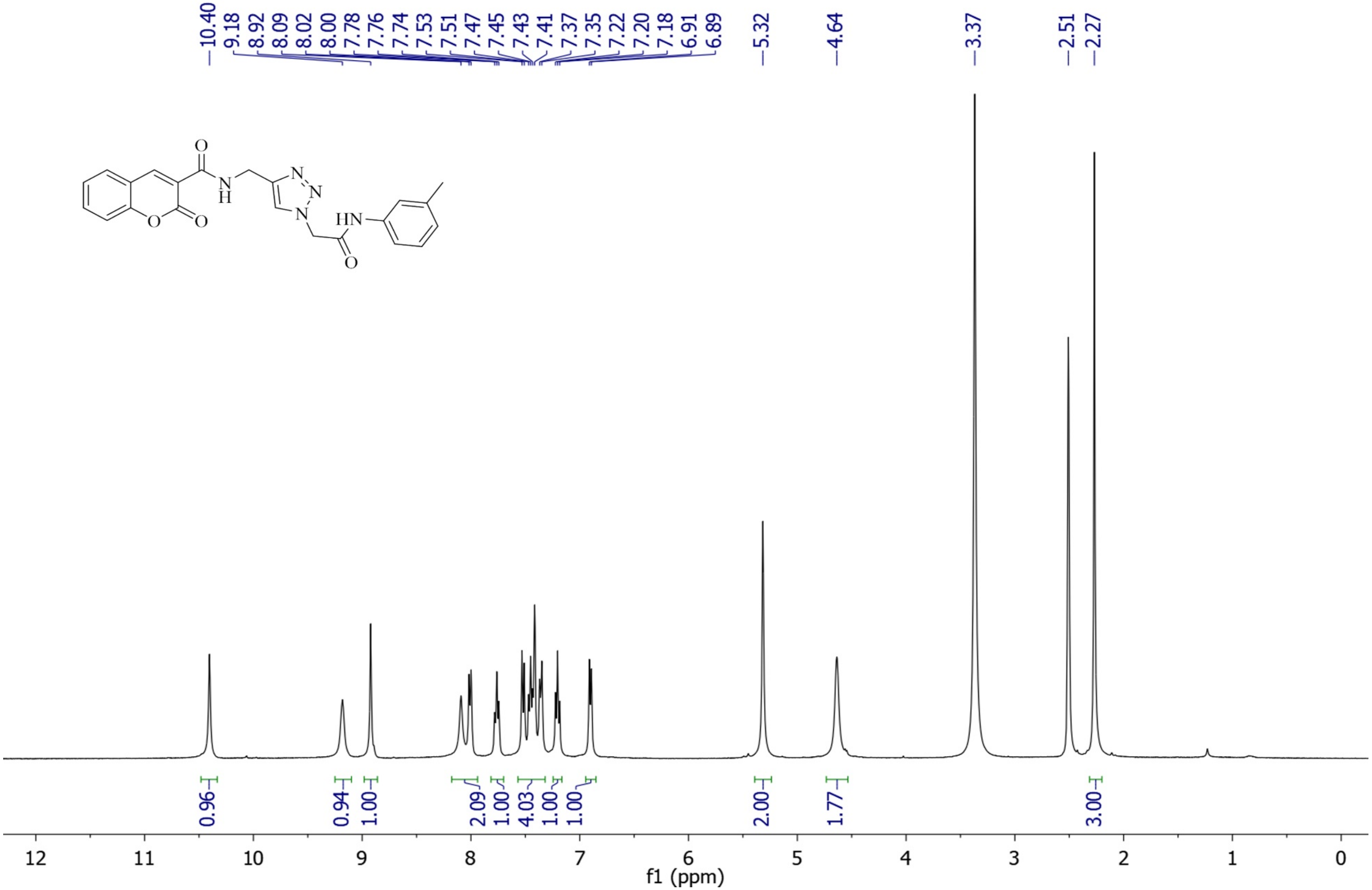

^{13}C NMR spectrum of 2-oxo-N-((1-(2-oxo-2-(m-tolylamino)ethyl)-1H-1,2,3-triazol-4-yl)methyl)-2H-chromene-3-carboxamide (**12c**)

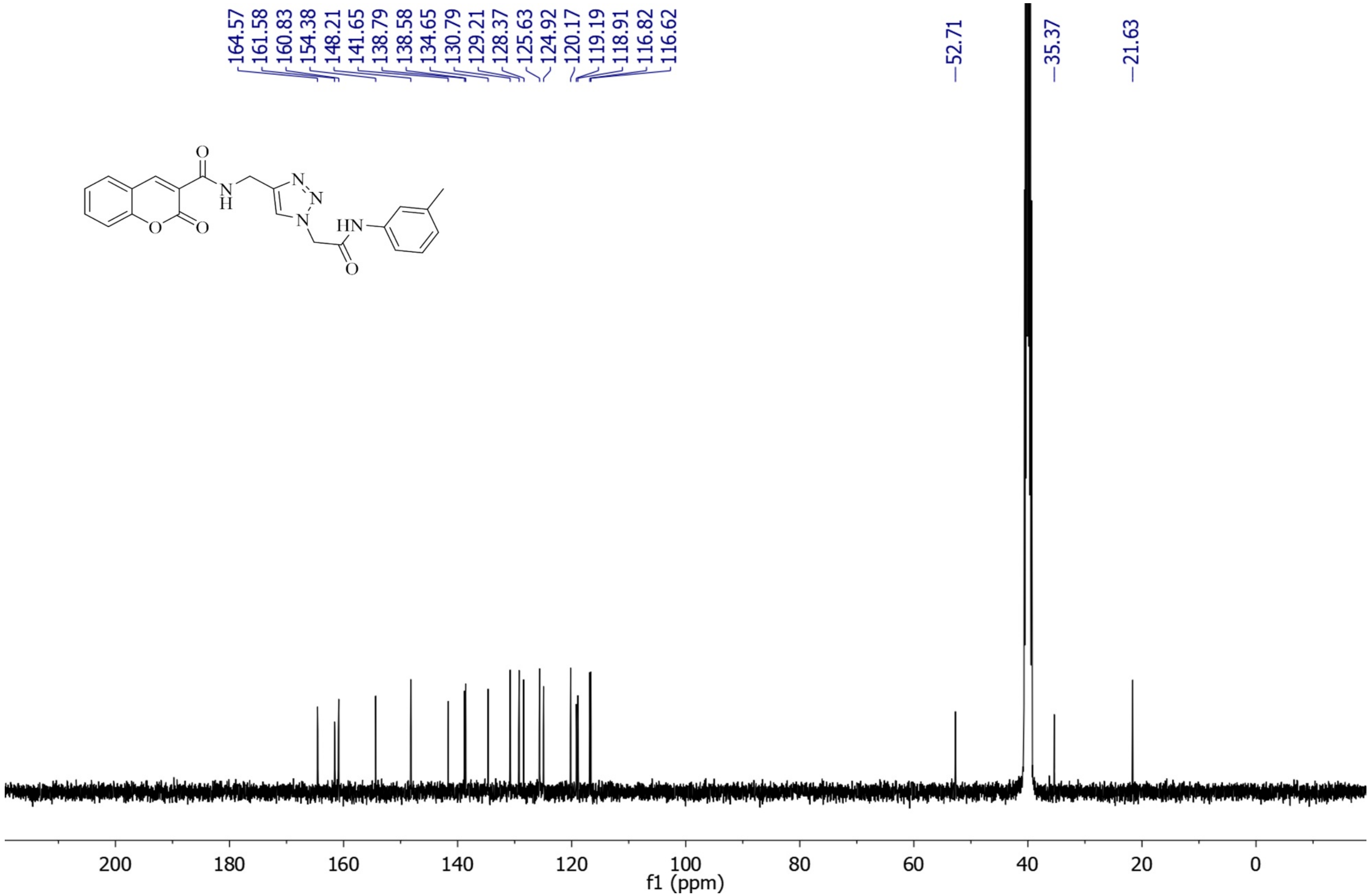

^{1}H NMR spectrum of 2-oxo-N-((1-(2-oxo-2-(p-tolylamino)ethyl)-1H-1,2,3-triazol-4-yl)methyl)-2H-chromene-3-carboxamide (**12d**)

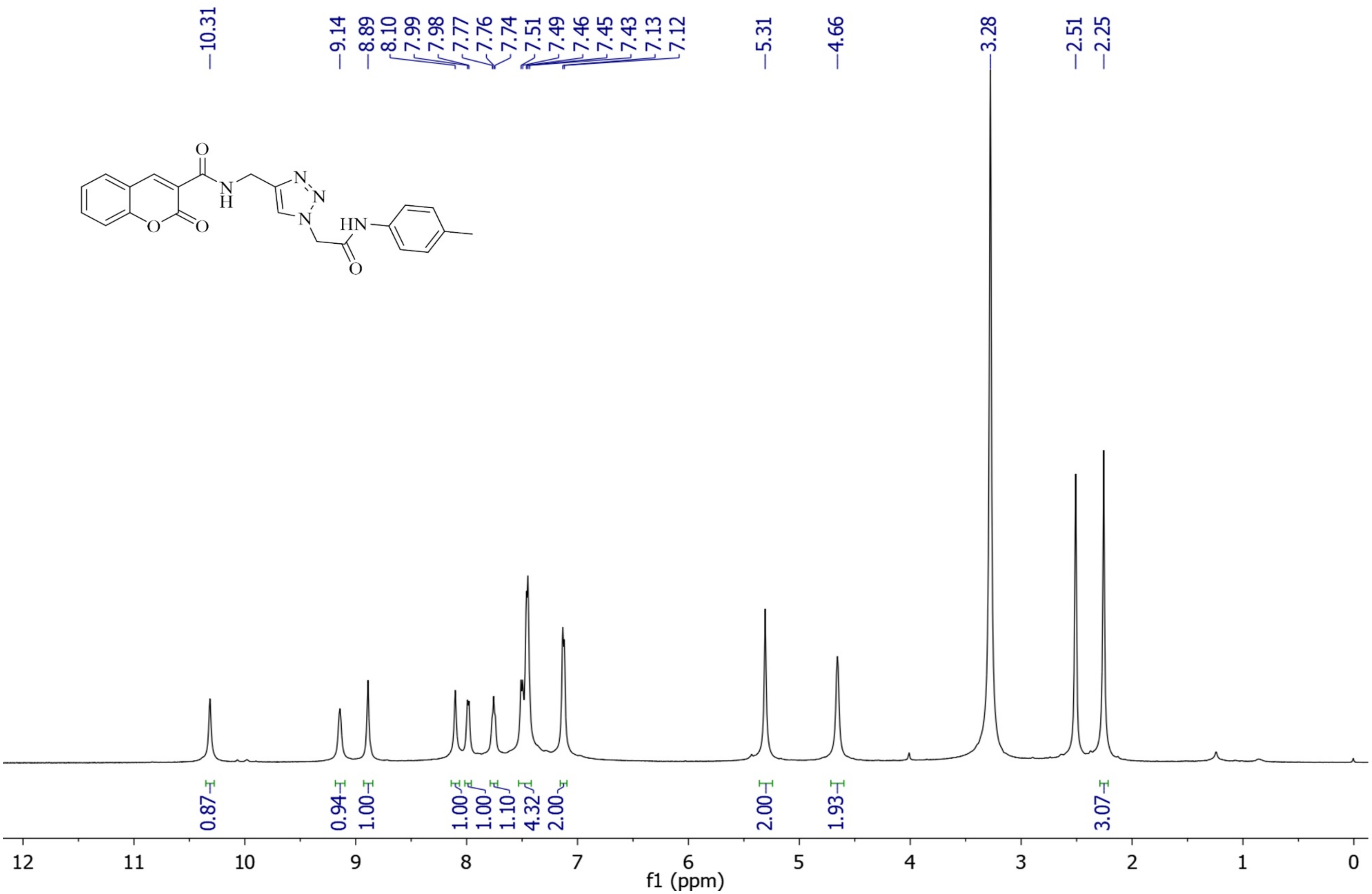

^{13}C NMR spectrum of 2-oxo-N-((1-(2-oxo-2-(p-tolylamino)ethyl)-1H-1,2,3-triazol-4-yl)methyl)-2H-chromene-3-carboxamide (**12d**)

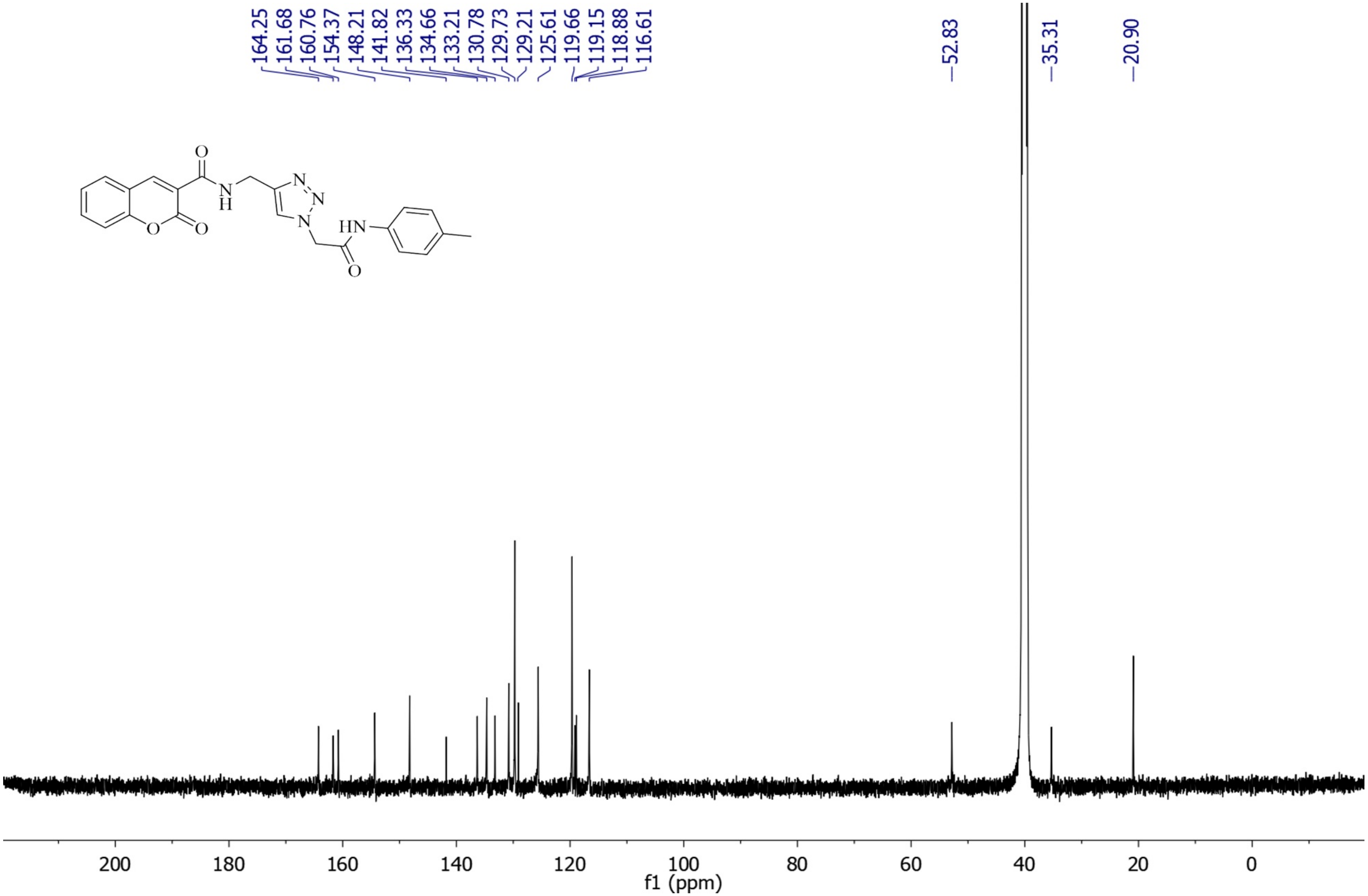

^{1}H NMR spectrum of N-((1-(2-((2-methoxyphenyl)amino)-2-oxoethyl)-1H-1,2,3-triazol-4-yl)methyl)-2-oxo-2H-chromene-3-carboxamide (**12e**)

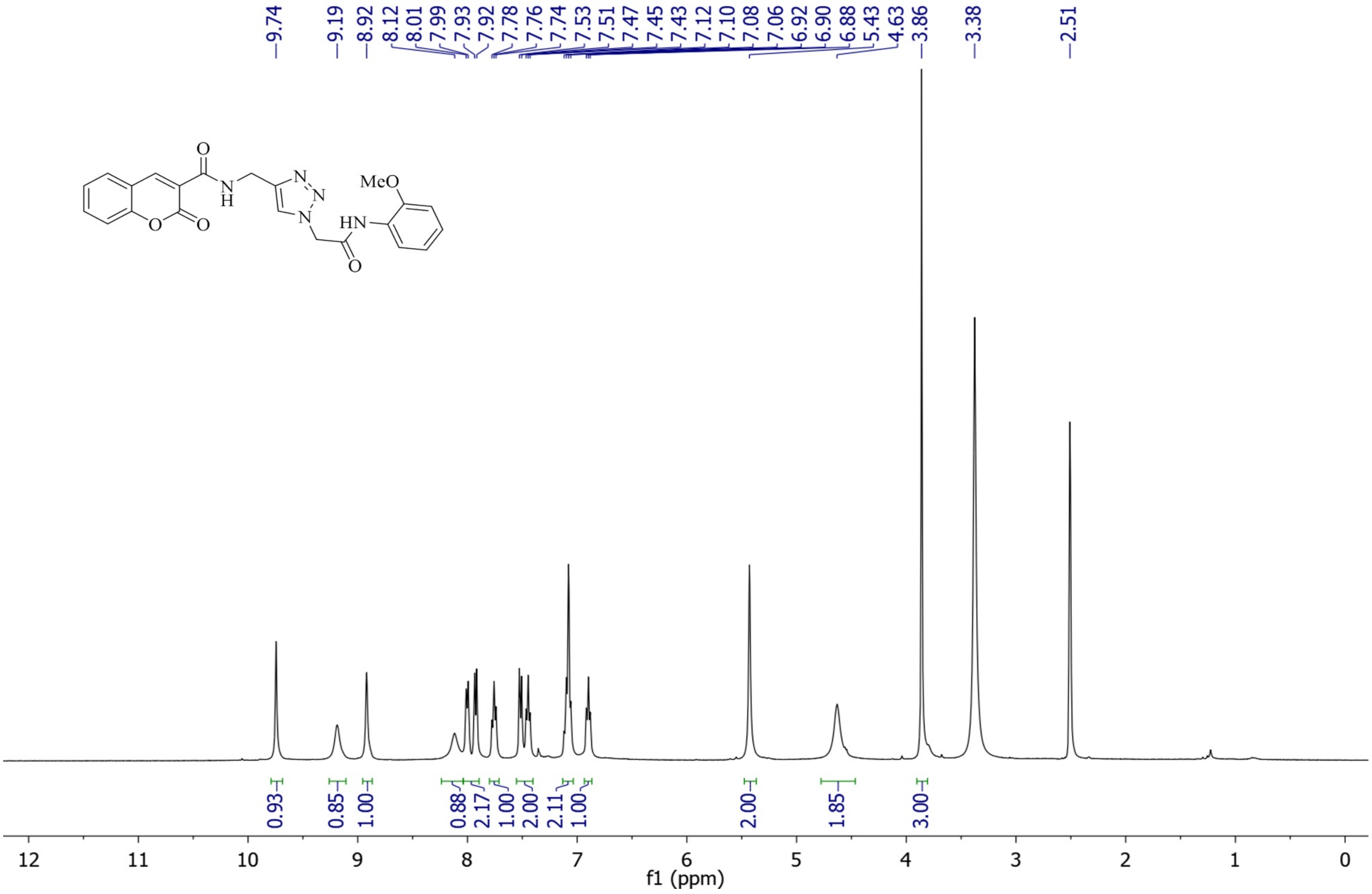

^{13}C NMR spectrum of N-((1-(2-((2-methoxyphenyl)amino)-2-oxoethyl)-1H-1,2,3-triazol-4-yl)methyl)-2-oxo-2H-chromene-3-carboxamide (**12e**)

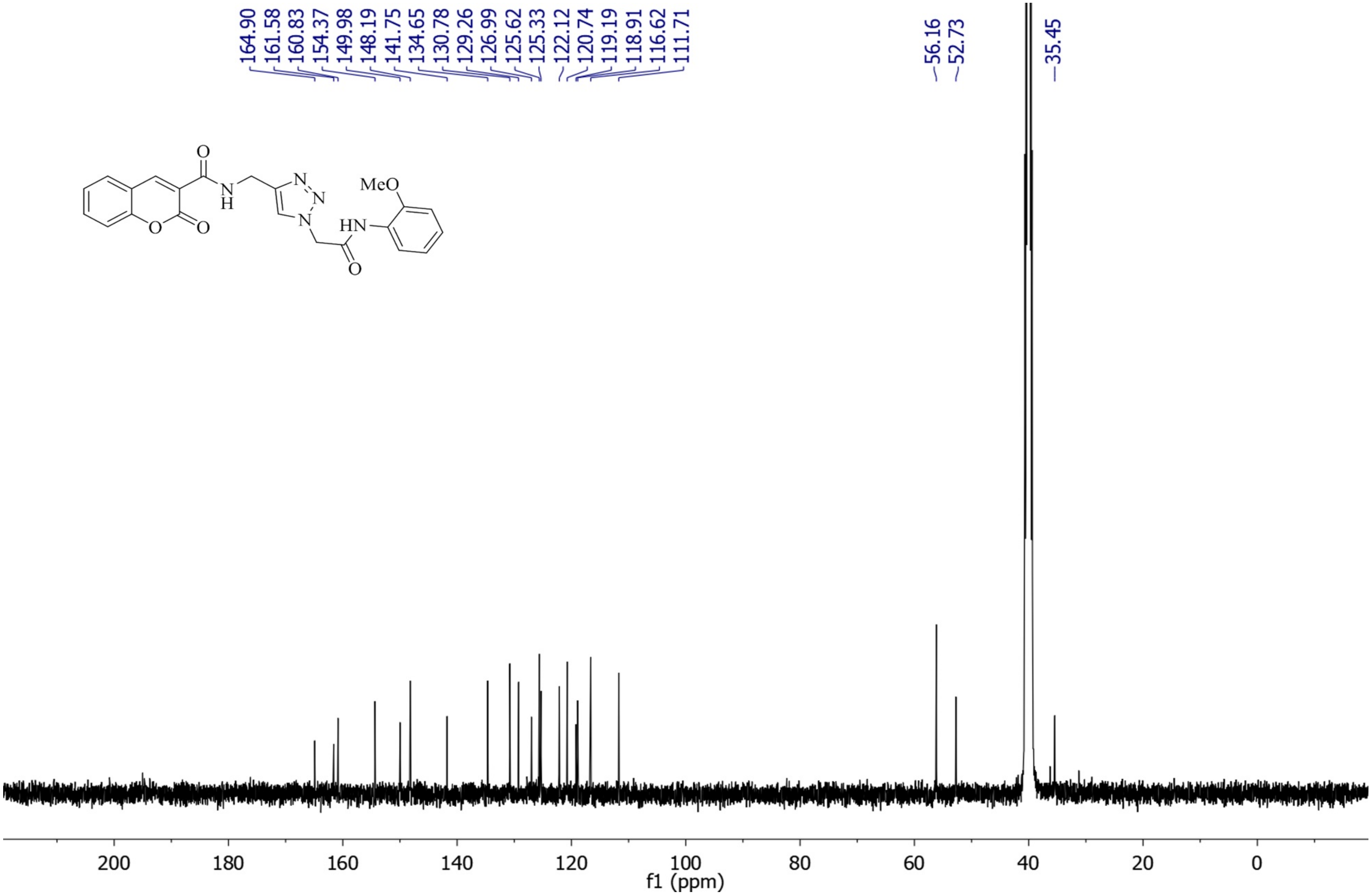

^{1}H NMR spectrum of N-((1-(2-((3-methoxyphenyl)amino)-2-oxoethyl)-1H-1,2,3-triazol-4-yl)methyl)-2-oxo-2H-chromene-3-carboxamide (**12f**)

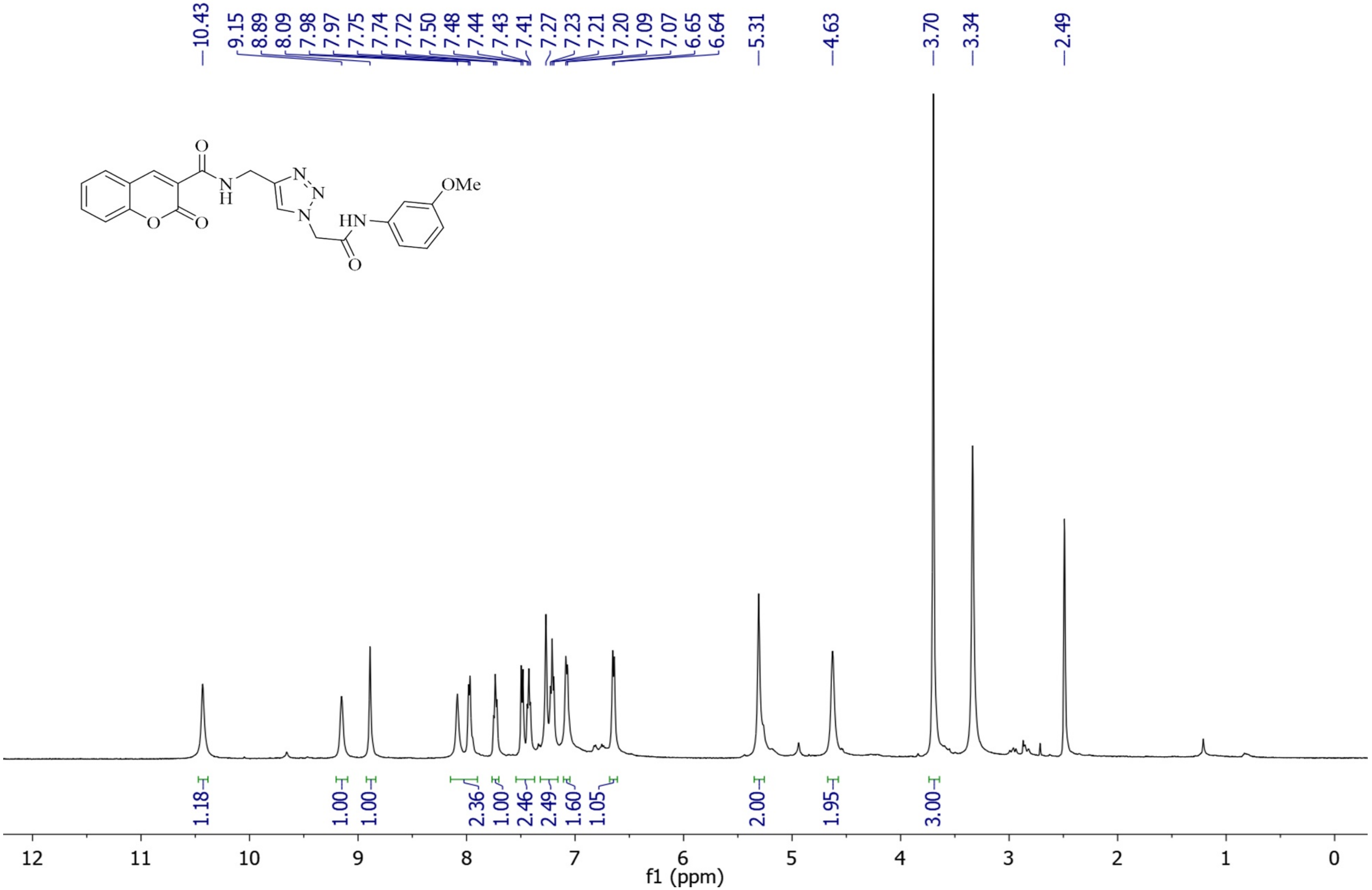

^{13}C NMR spectrum of N-((1-(2-((3-methoxyphenyl)amino)-2-oxoethyl)-1H-1,2,3-triazol-4-yl)methyl)-2-oxo-2H-chromene-3-carboxamide (**12f**)

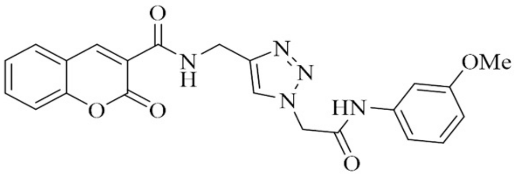

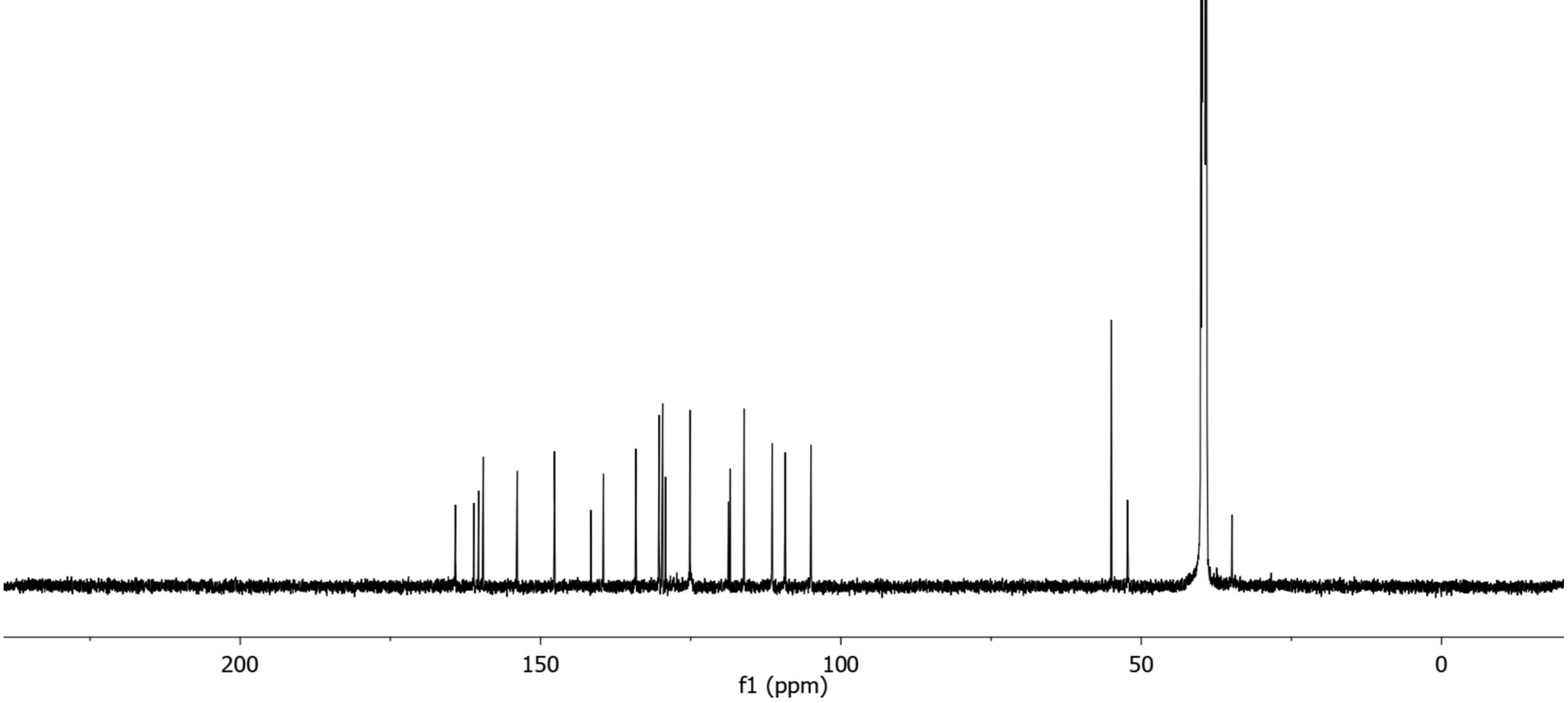

^{1}H NMR spectrum of N-((1-(2-((4-methoxyphenyl)amino)-2-oxoethyl)-1H-1,2,3-triazol-4-yl)methyl)-2-oxo-2H-chromene-3-carboxamide (**12g**)

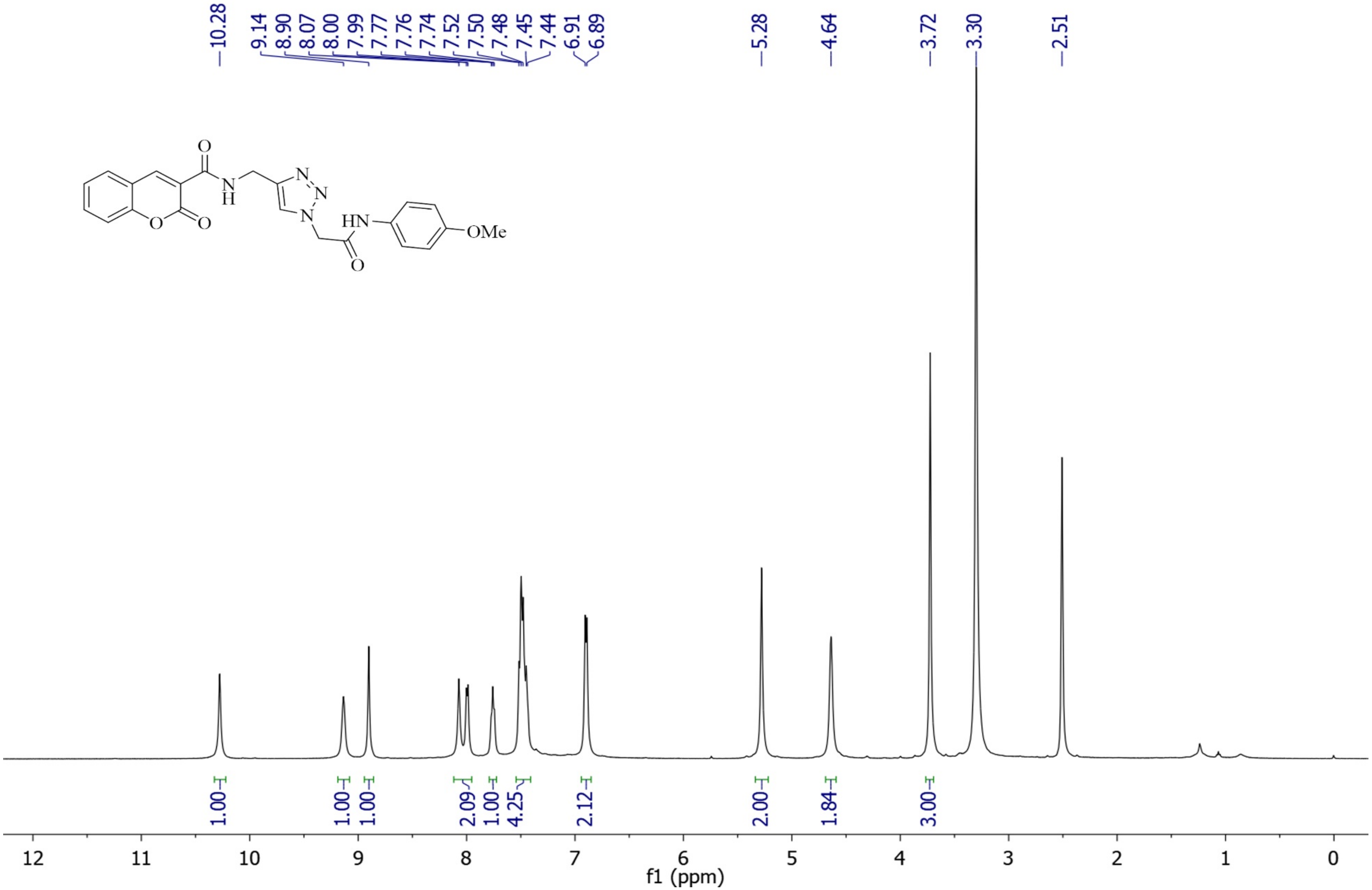

^{13}C NMR spectrum of N-((1-(2-((4-methoxyphenyl)amino)-2-oxoethyl)-1H-1,2,3-triazol-4-yl)methyl)-2-oxo-2H-chromene-3-

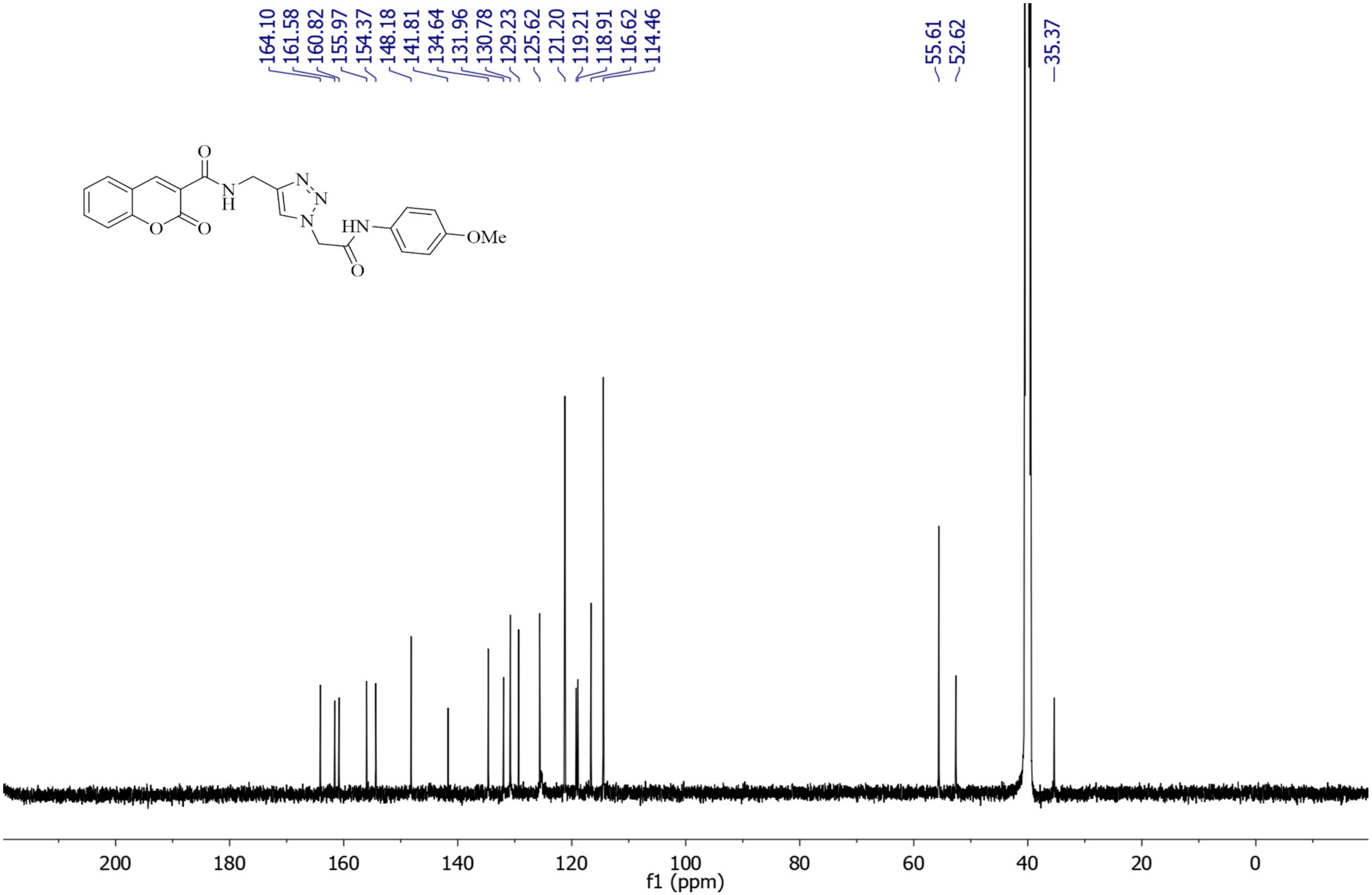

carboxamide (**12g**)

^{1}H NMR spectrum of 2-oxo-N-((1-(2-oxo-2-((3,4,5-trimethoxyphenyl)amino)ethyl)-1H-1,2,3-triazol-4-yl)methyl)-2H-chromene-3-

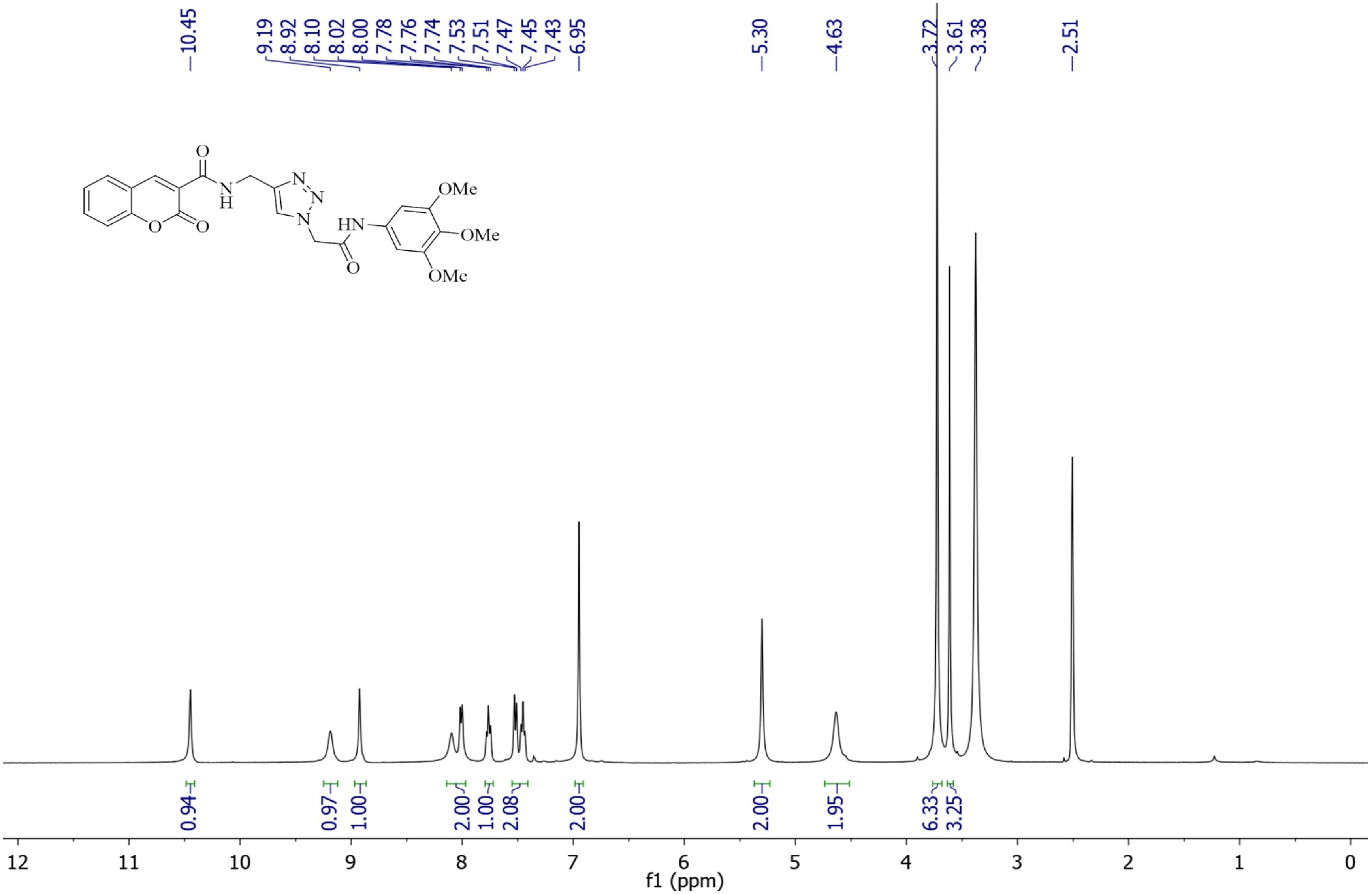

carboxamide (**12h**)

^{13}C NMR spectrum of 2-oxo-N-((1-(2-oxo-2-((3,4,5-trimethoxyphenyl)amino)ethyl)-1H-1,2,3-triazol-4-yl)methyl)-2H-chromene-3-carboxamide (**12h**)

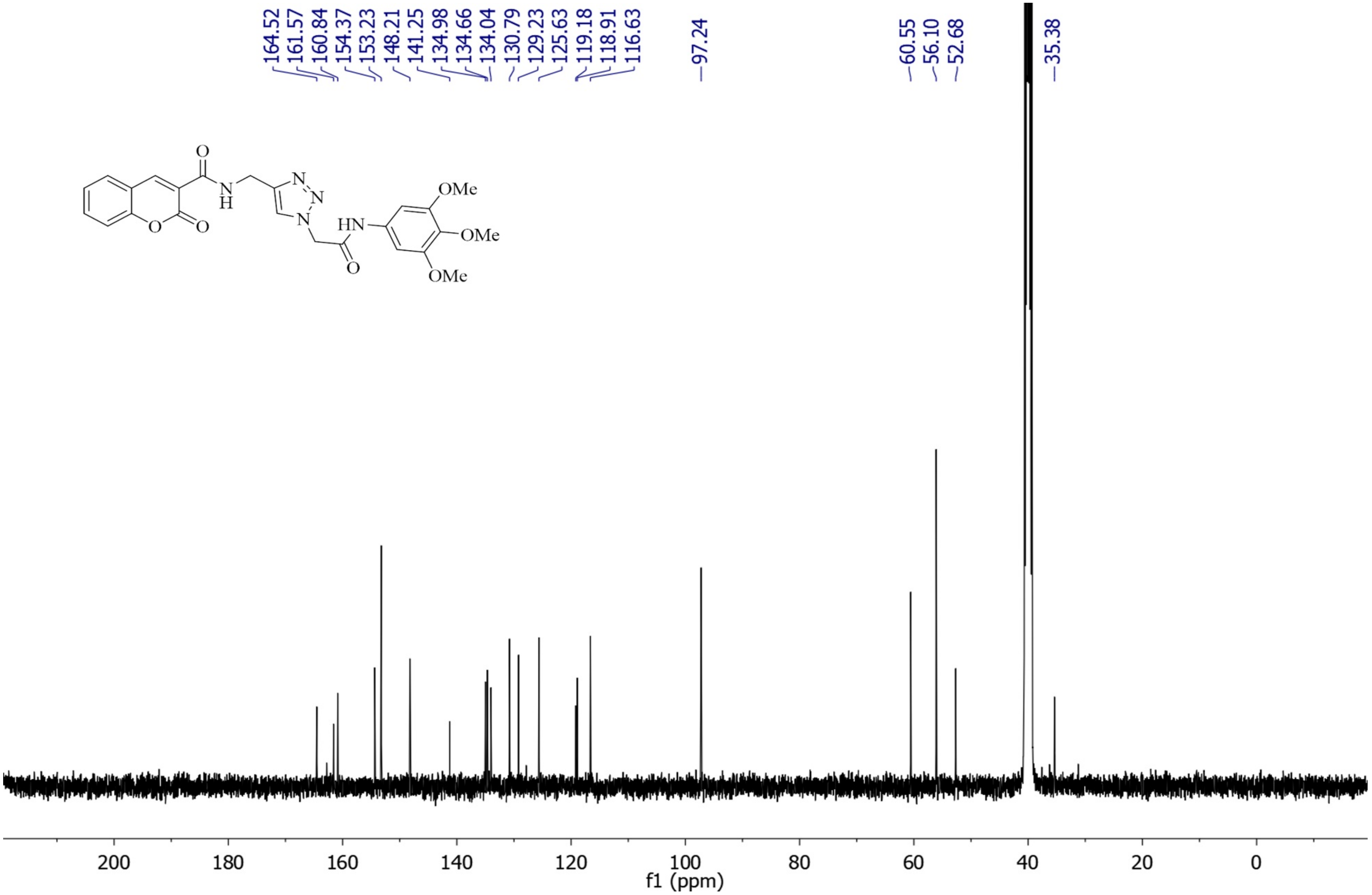

^{1}H NMR spectrum of N-((1-(2-((2-fluorophenyl)amino)-2-oxoethyl)-1H-1,2,3-triazol-4-yl)methyl)-2-oxo-2H-chromene-3-carboxamide (**12i**)

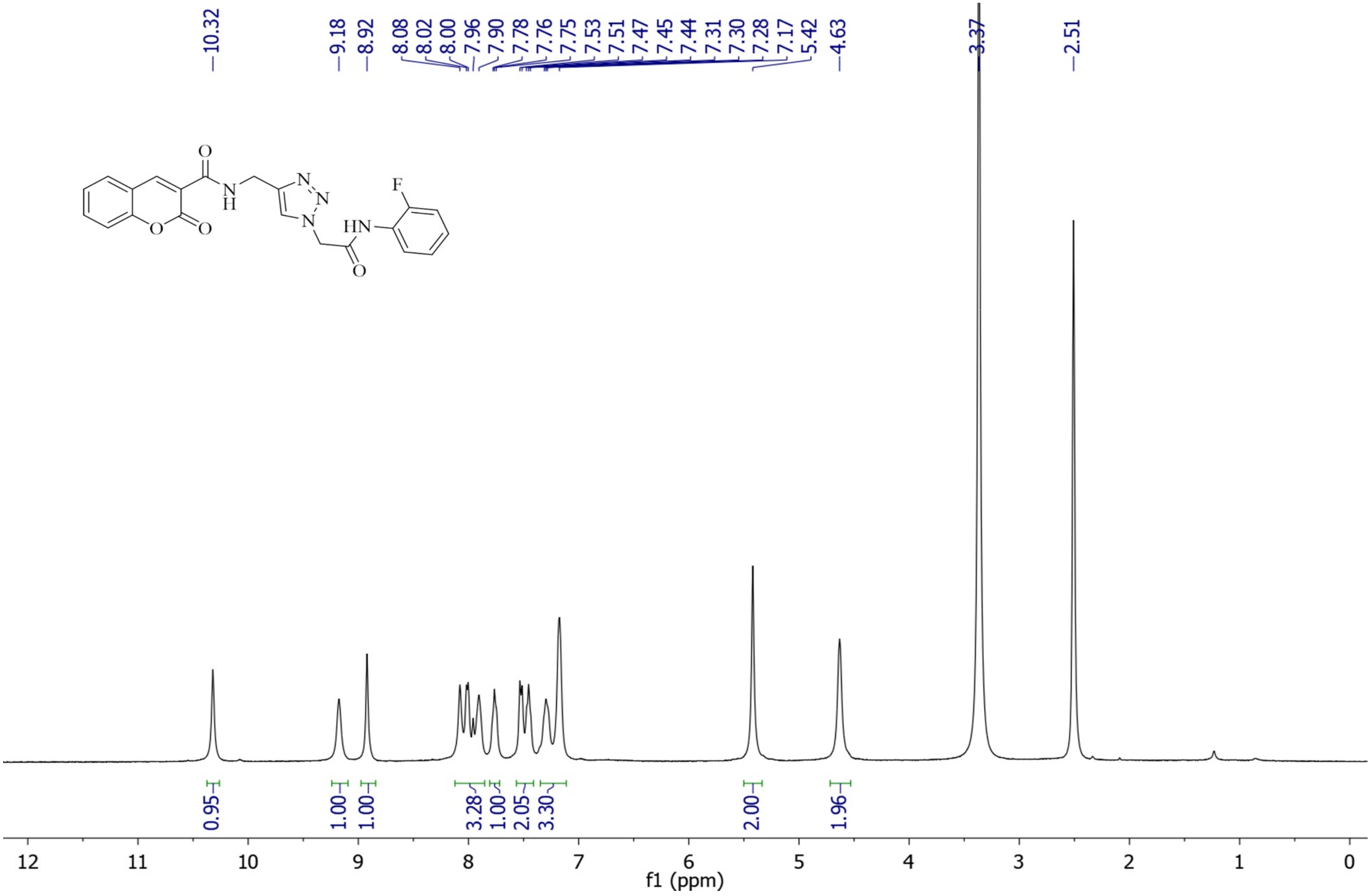

^{13}C NMR spectrum of N-((1-(2-((2-fluorophenyl)amino)-2-oxoethyl)-1H-1,2,3-triazol-4-yl)methyl)-2-oxo-2H-chromene-3-carboxamide (**12i**)

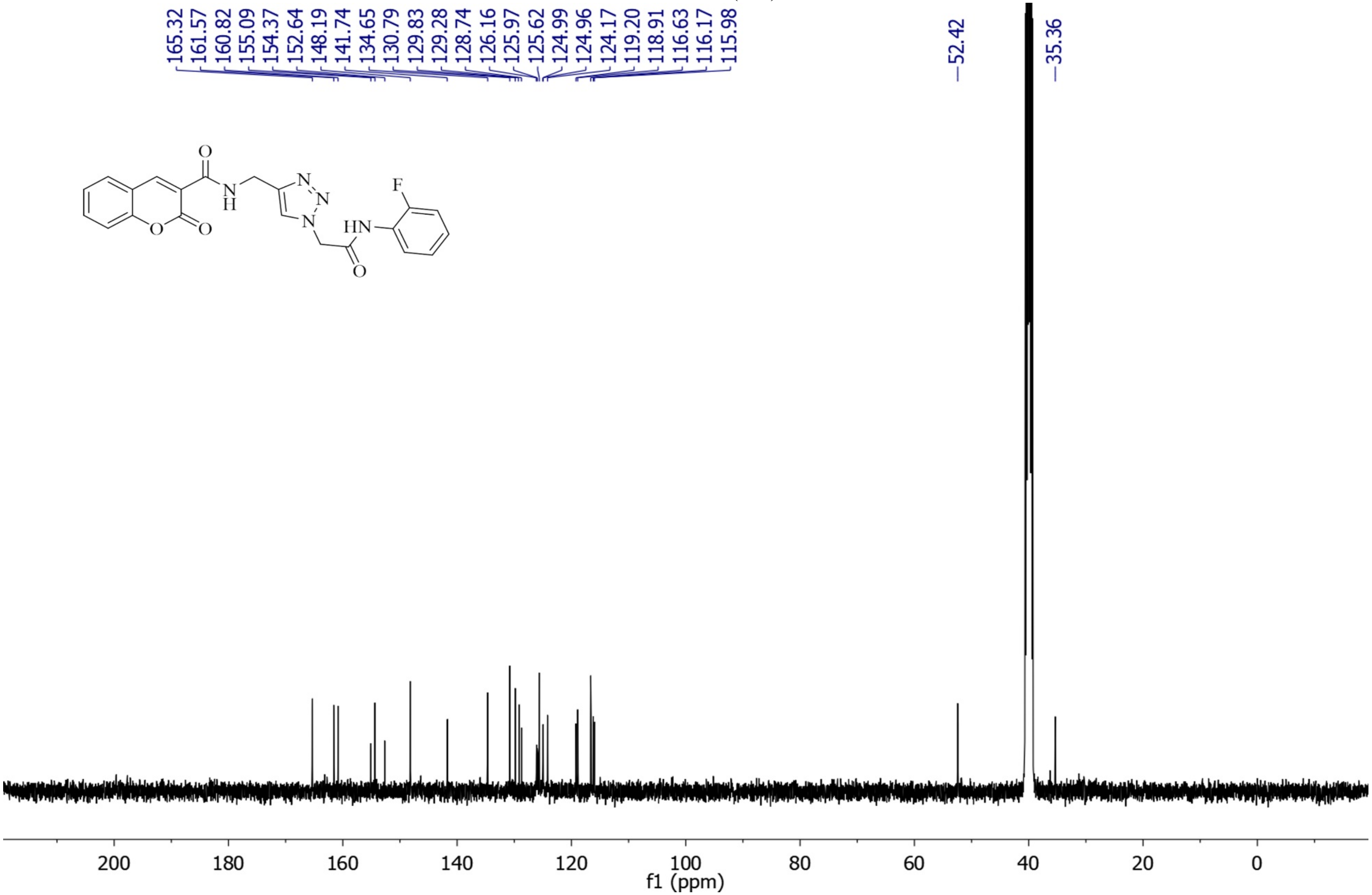

^{1}H NMR spectrum of N-((1-(2-((4-fluorophenyl)amino)-2-oxoethyl)-1H-1,2,3-triazol-4-yl)methyl)-2-oxo-2H-chromene-3-

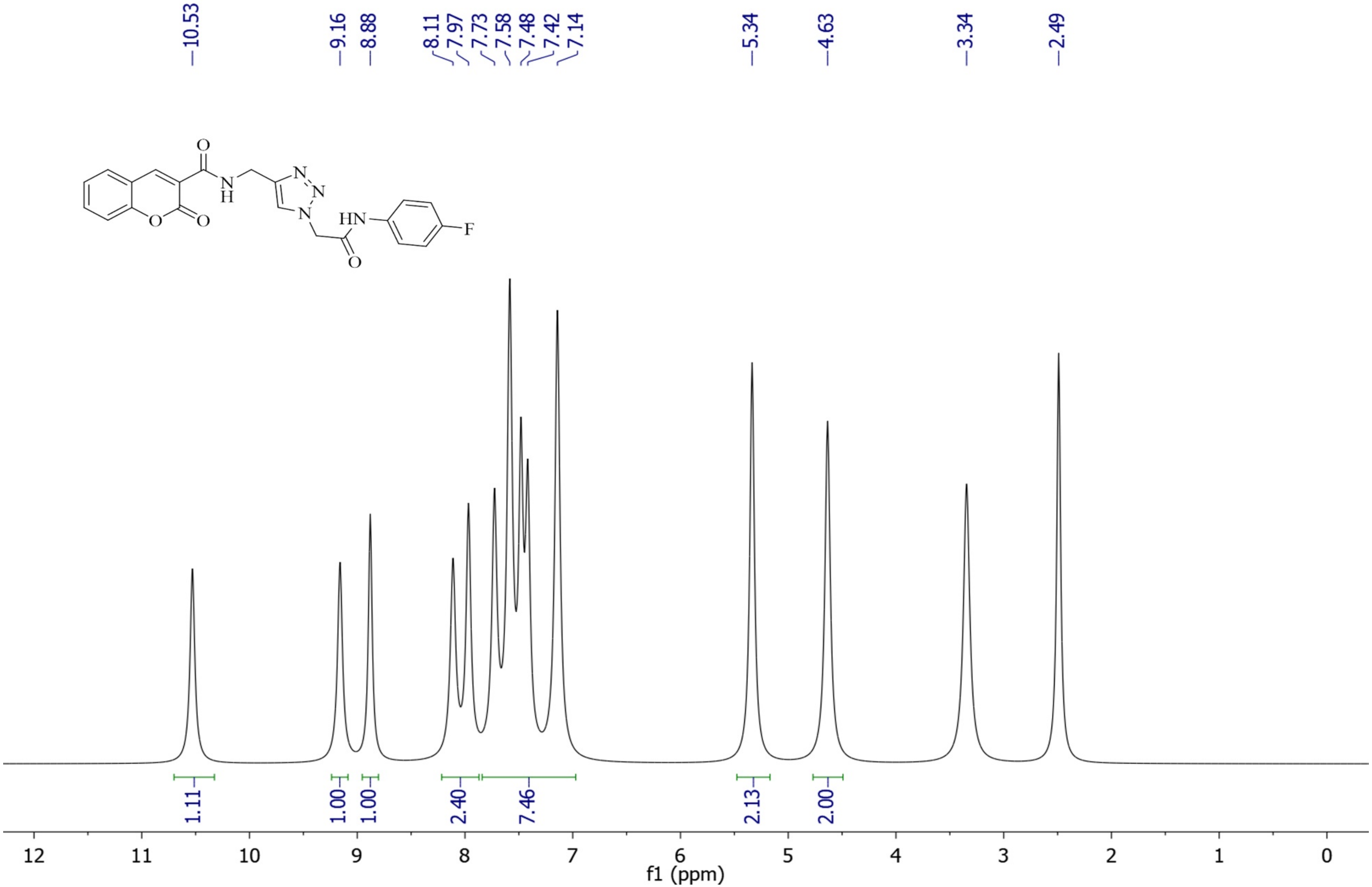

carboxamide (**12j**)

^{13}C NMR spectrum of N-((1-(2-((4-fluorophenyl)amino)-2-oxoethyl)-1H-1,2,3-triazol-4-yl)methyl)-2-oxo-2H-chromene-3-

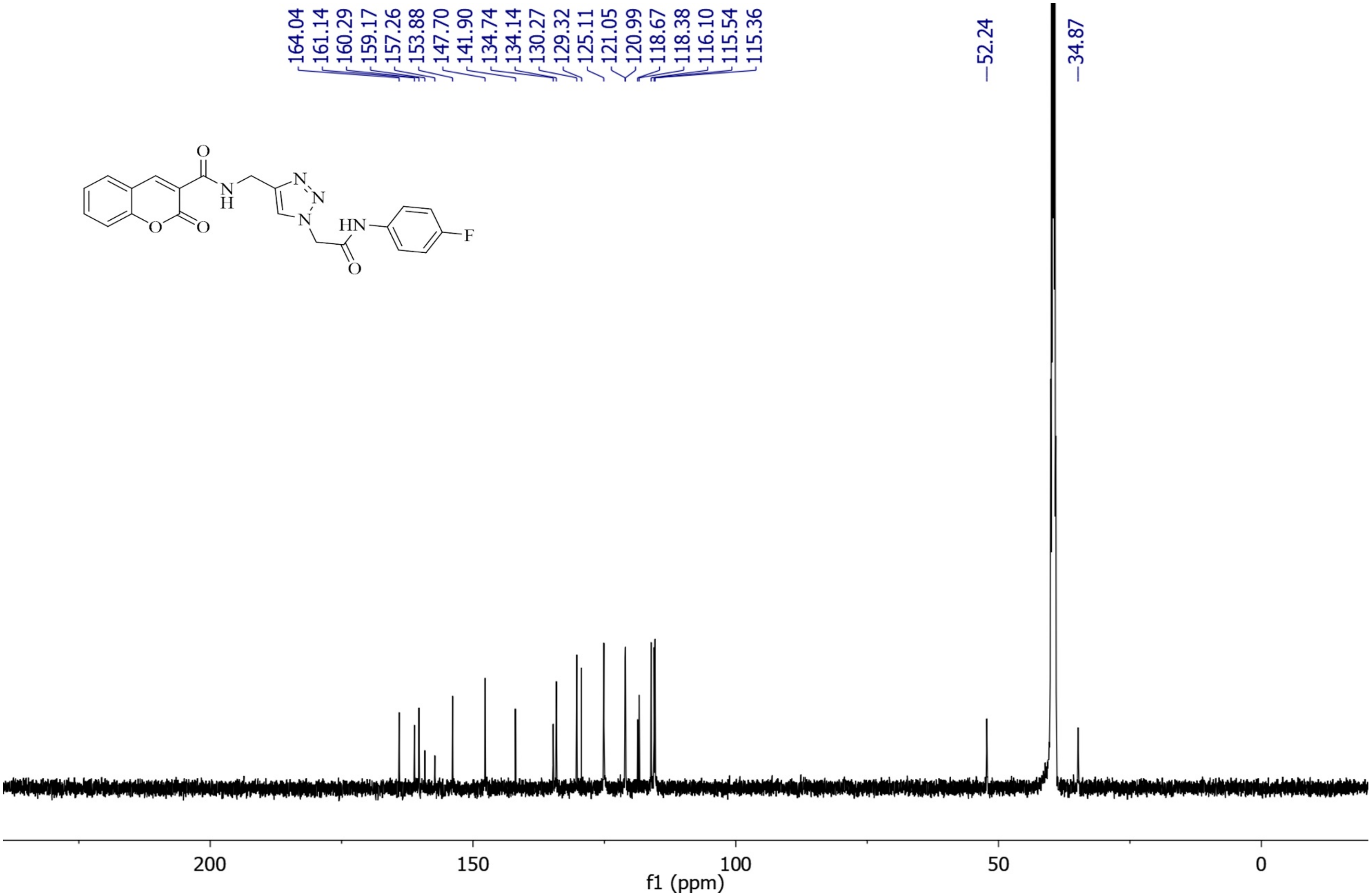

carboxamide (**12j**)

^{1}H NMR spectrum of N-((1-(2-((2-chlorophenyl)amino)-2-oxoethyl)-1H-1,2,3-triazol-4-yl)methyl)-2-oxo-2H-chromene-3-

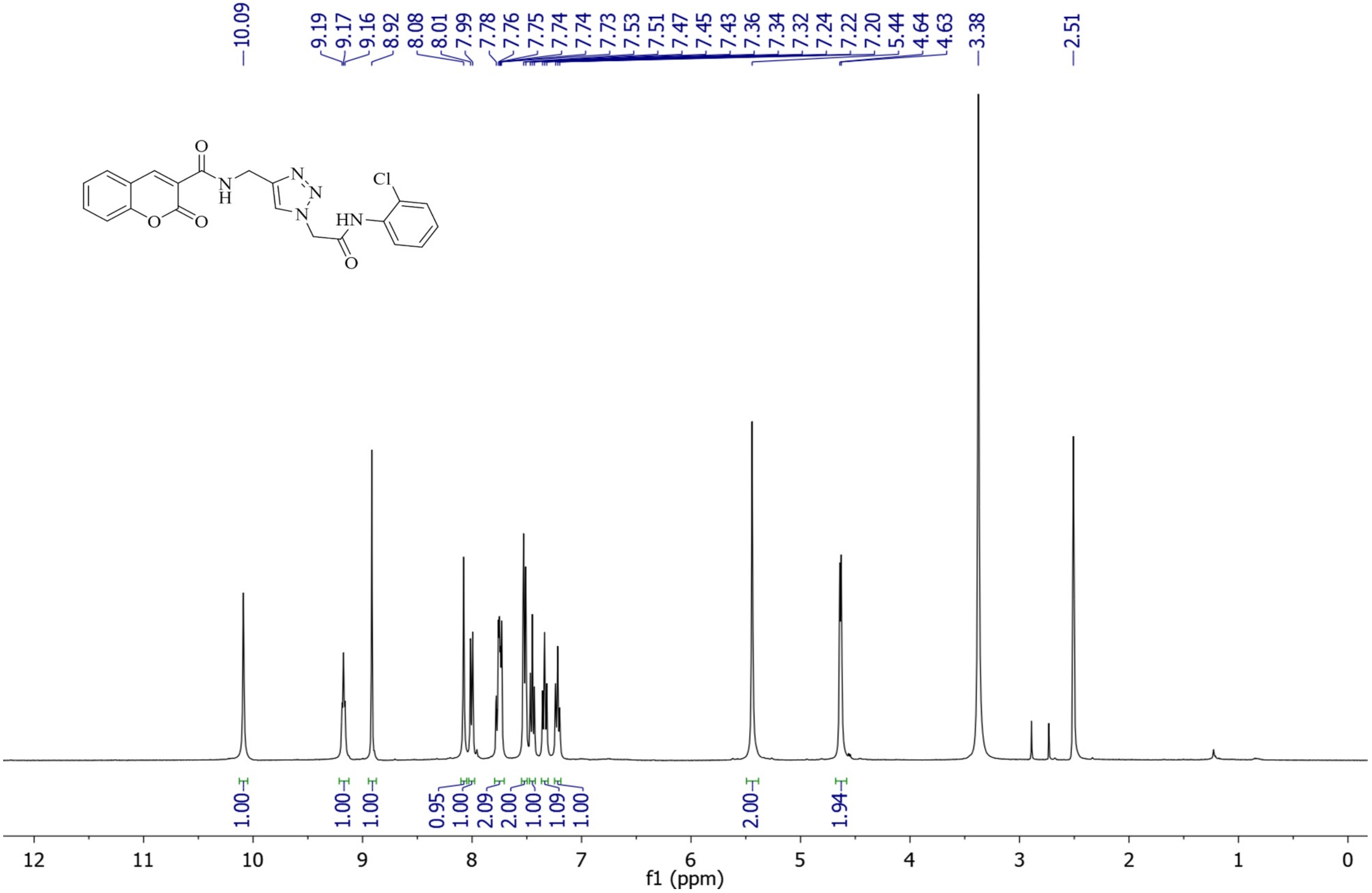

carboxamide (**12k**)

^{13}C NMR spectrum of N-((1-(2-((2-chlorophenyl)amino)-2-oxoethyl)-1H-1,2,3-triazol-4-yl)methyl)-2-oxo-2H-chromene-3-

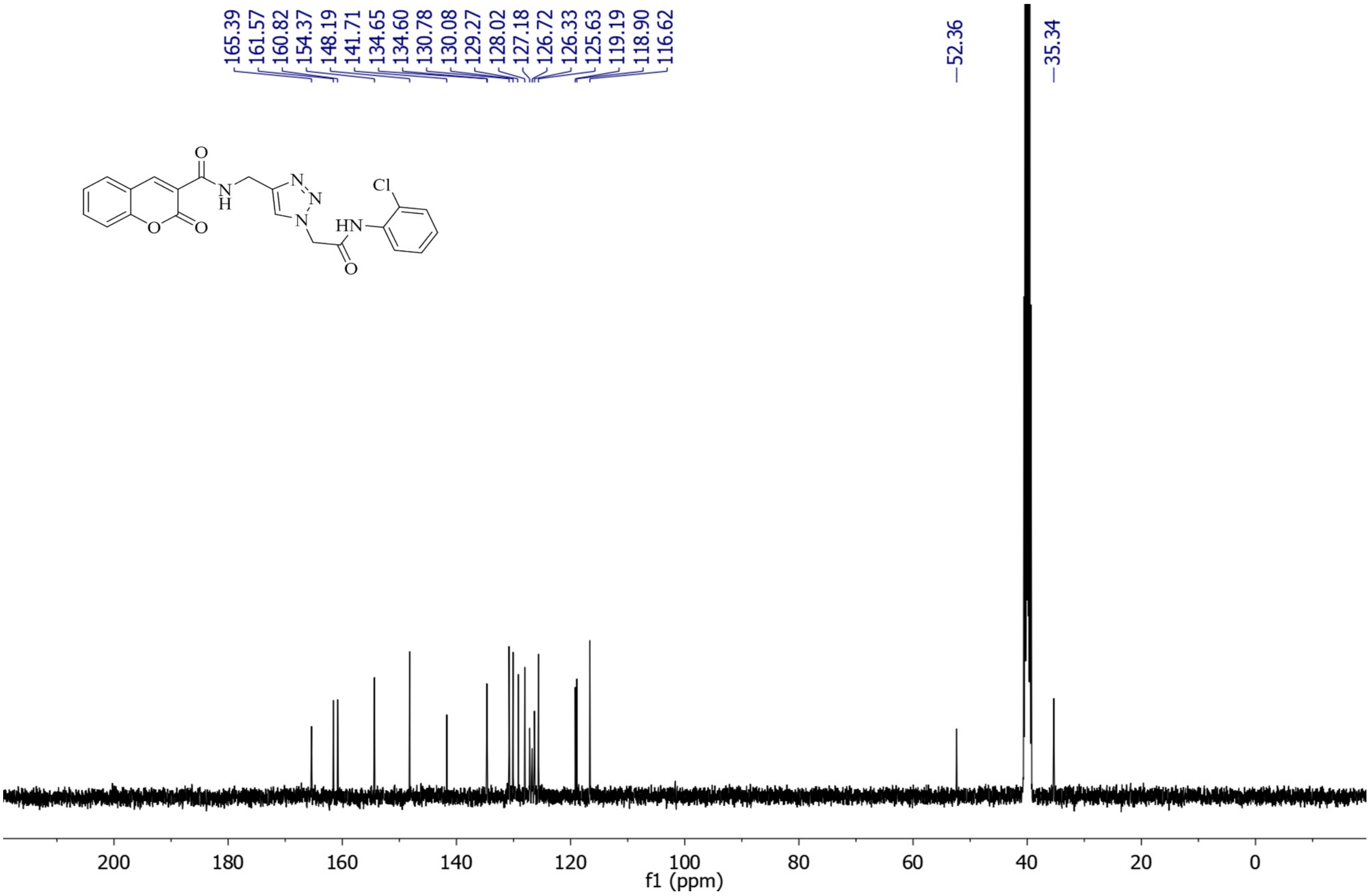

carboxamide (**12k**)

^{1}H NMR spectrum of N-((1-(2-((3-chlorophenyl)amino)-2-oxoethyl)-1H-1,2,3-triazol-4-yl)methyl)-2-oxo-2H-chromene-3-carboxamide (**12l**)

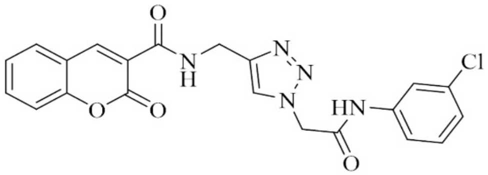

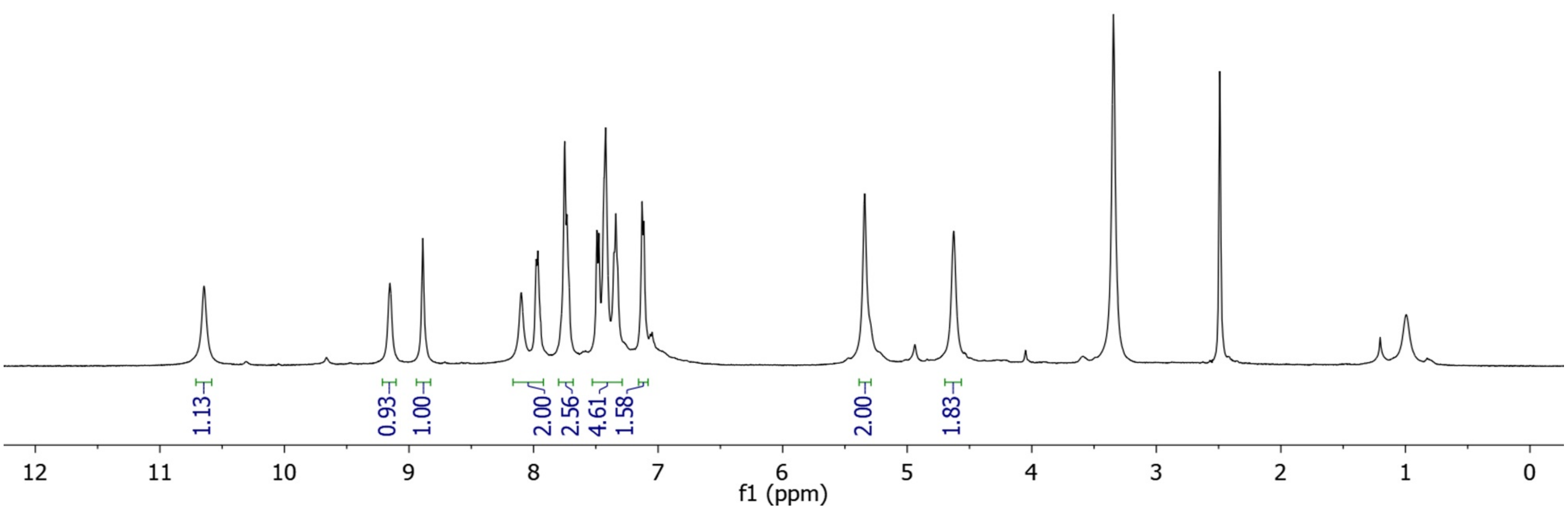

^{13}C NMR spectrum of N-((1-(2-((3-chlorophenyl)amino)-2-oxoethyl)-1H-1,2,3-triazol-4-yl)methyl)-2-oxo-2H-chromene-3-carboxamide (**12l**)

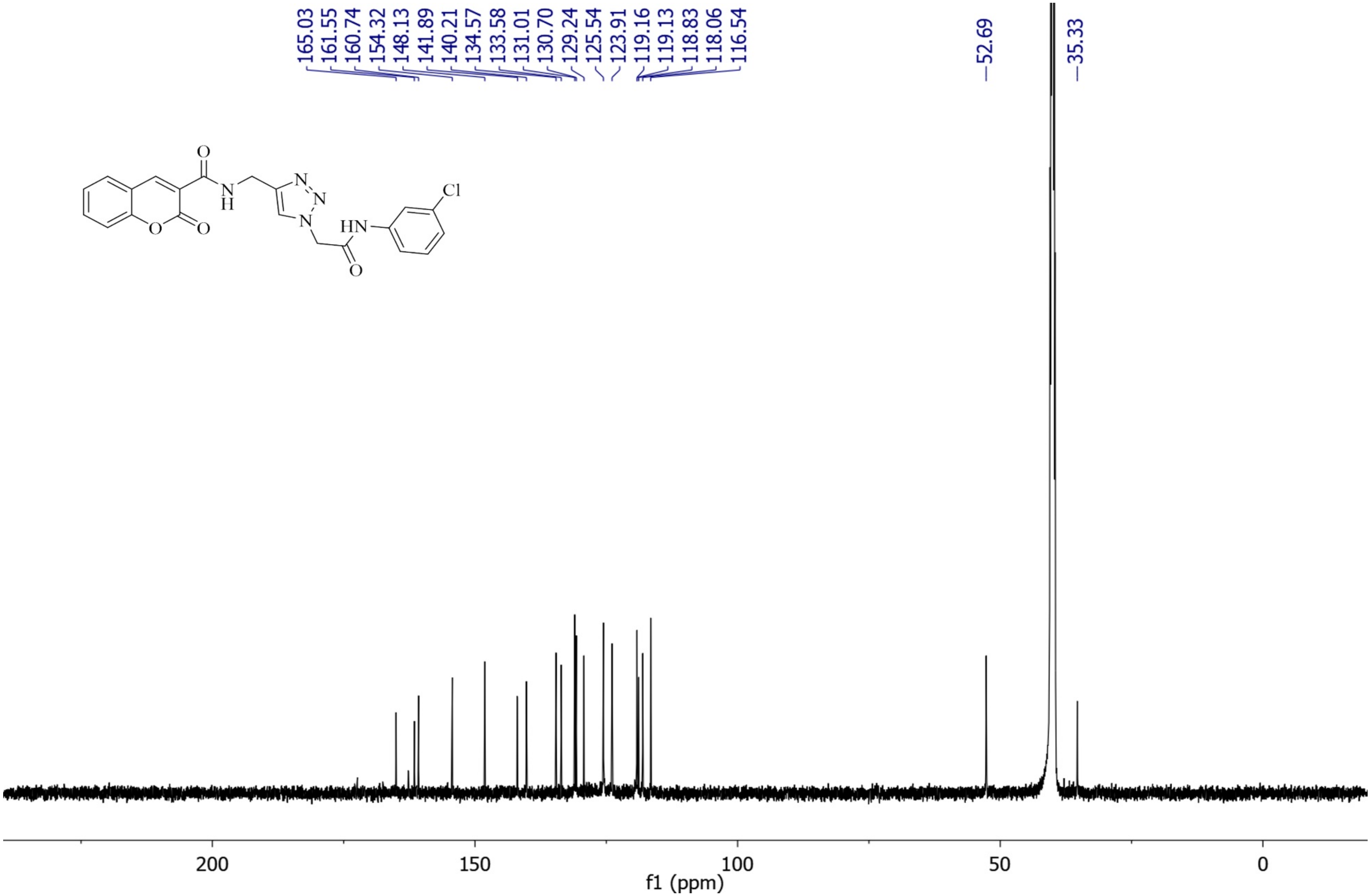

^{1}H NMR spectrum of N-((1-(2-((4-chlorophenyl)amino)-2-oxoethyl)-1H-1,2,3-triazol-4-yl)methyl)-2-oxo-2H-chromene-3-

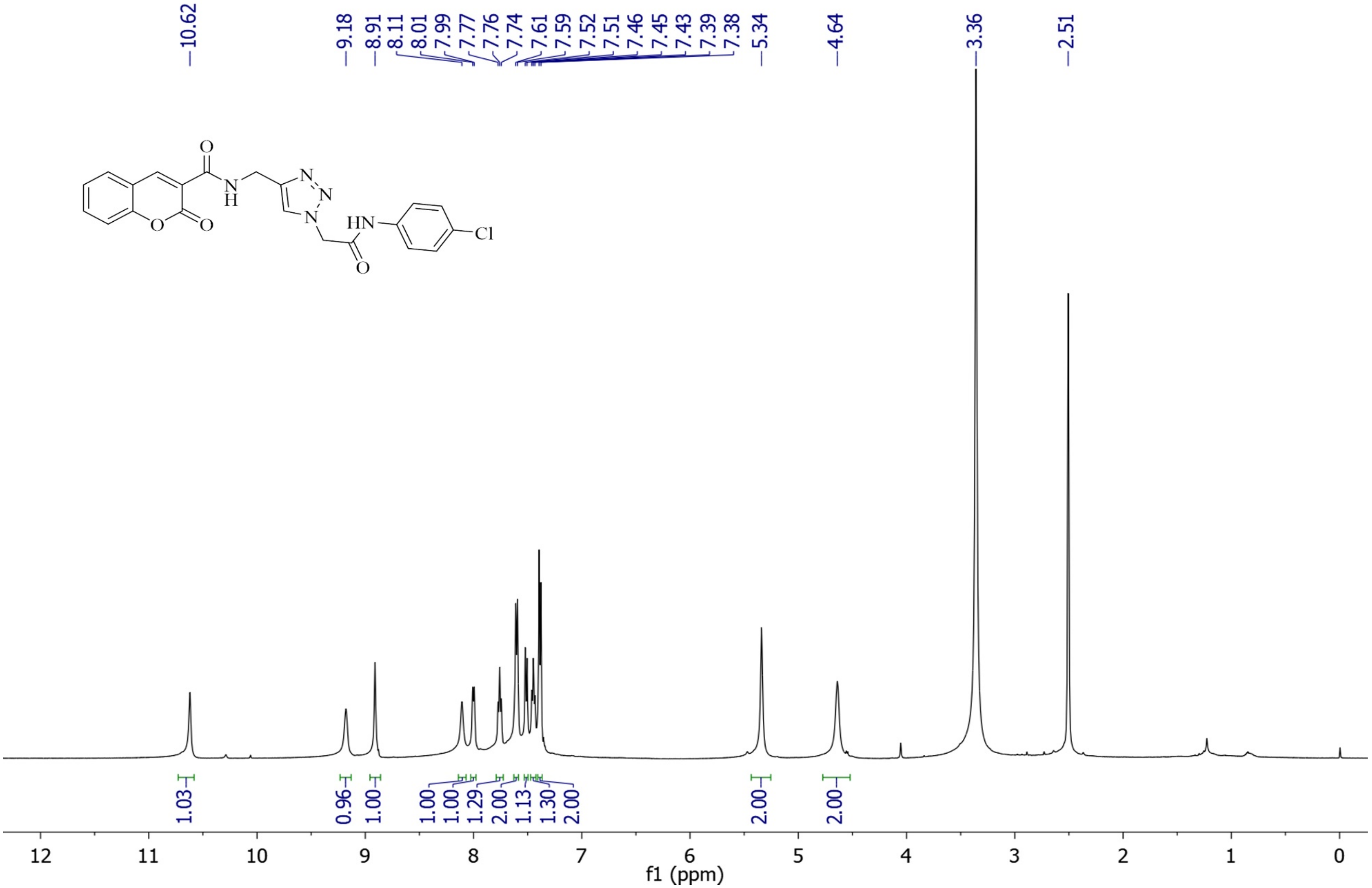

carboxamide (**12m**)

^{13}C NMR spectrum of N-((1-(2-((4-chlorophenyl)amino)-2-oxoethyl)-1H-1,2,3-triazol-4-yl)methyl)-2-oxo-2H-chromene-3-

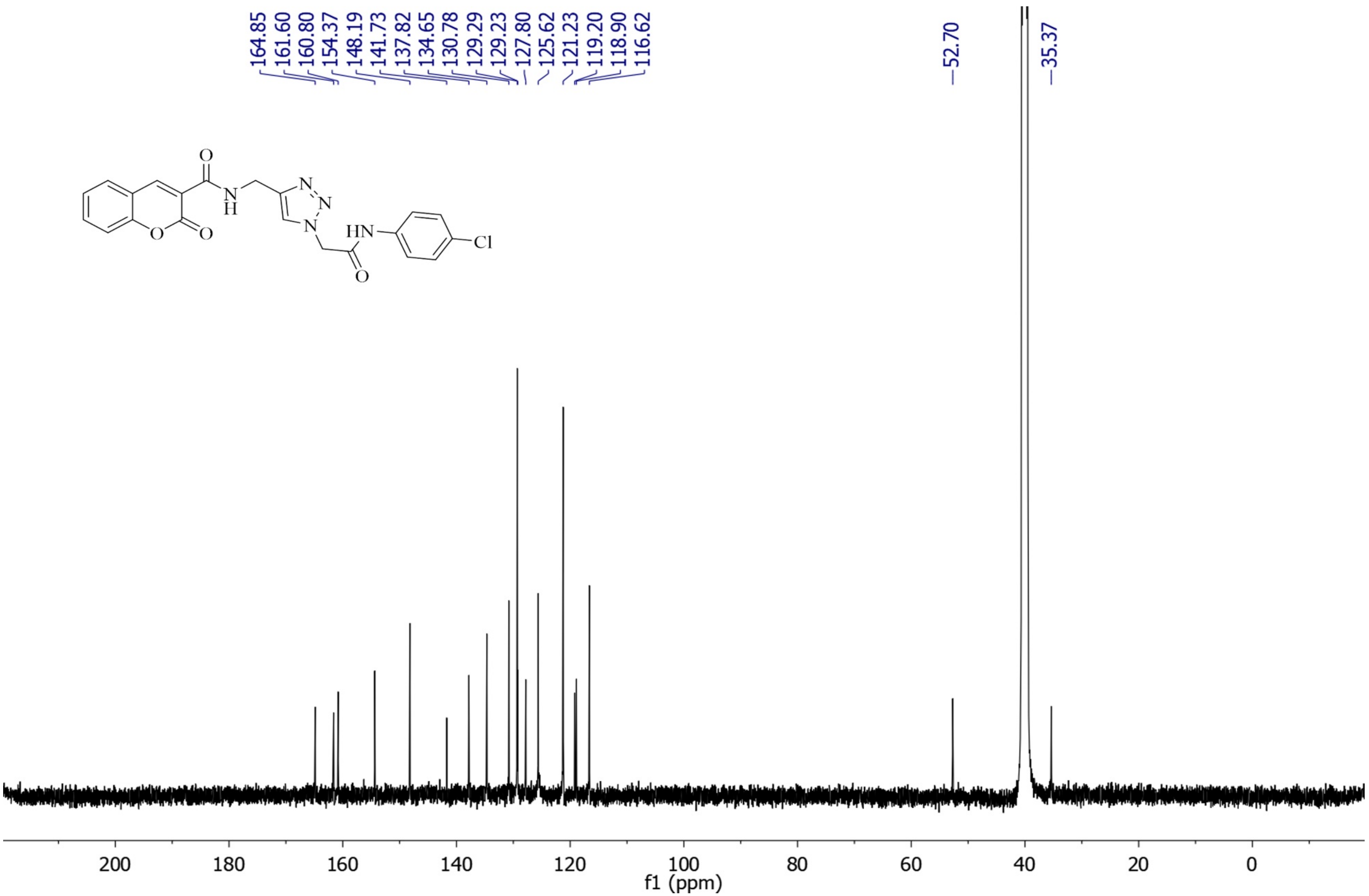

carboxamide (**12m**)

^{1}H NMR spectrum of N-((1-(2-((4-bromophenyl)amino)-2-oxoethyl)-1H-1,2,3-triazol-4-yl)methyl)-2-oxo-2H-chromene-3-

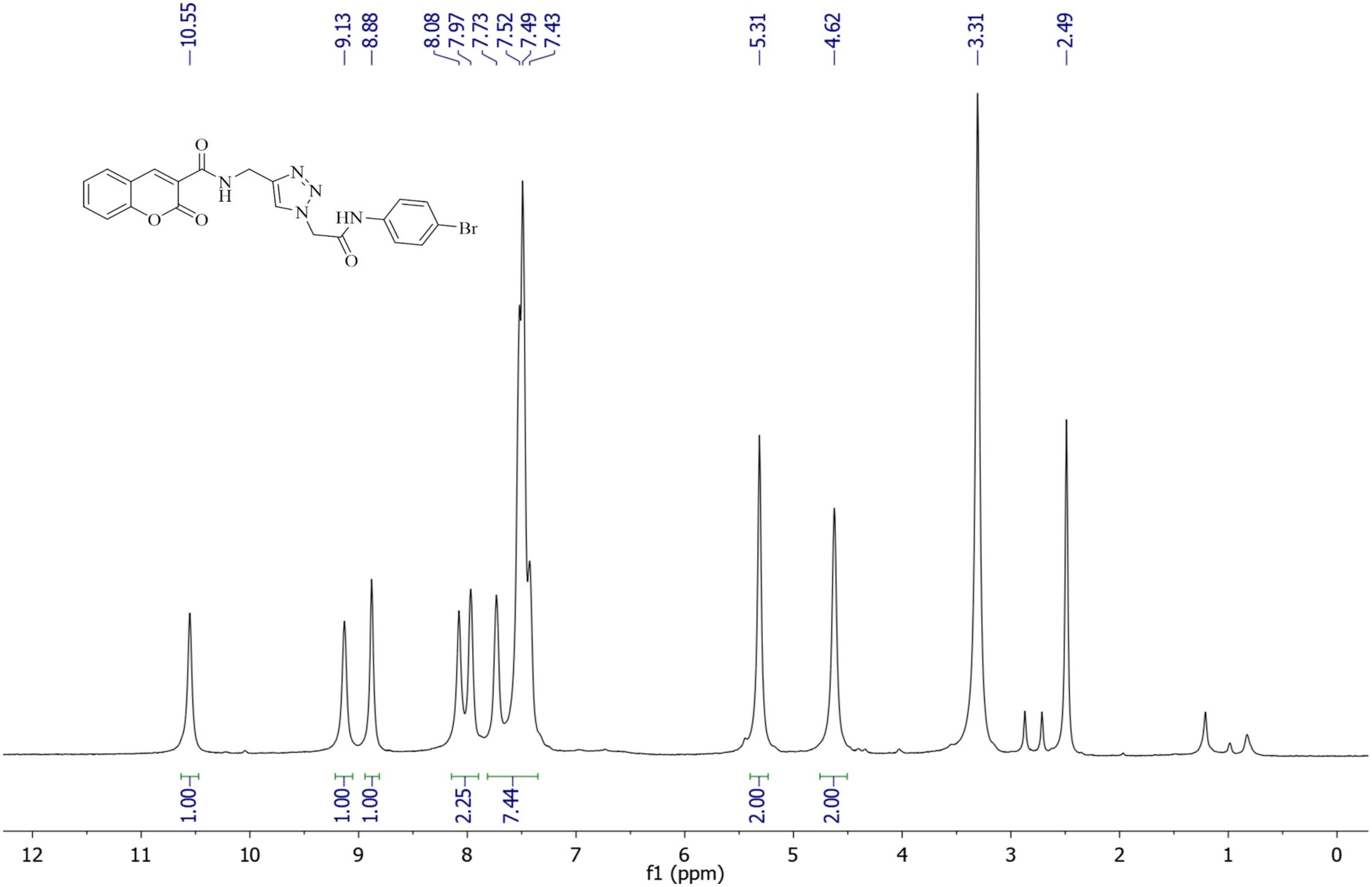

carboxamide (**12n**)

^{13}C NMR spectrum of N-((1-(2-((4-bromophenyl)amino)-2-oxoethyl)-1H-1,2,3-triazol-4-yl)methyl)-2-oxo-2H-chromene-3-

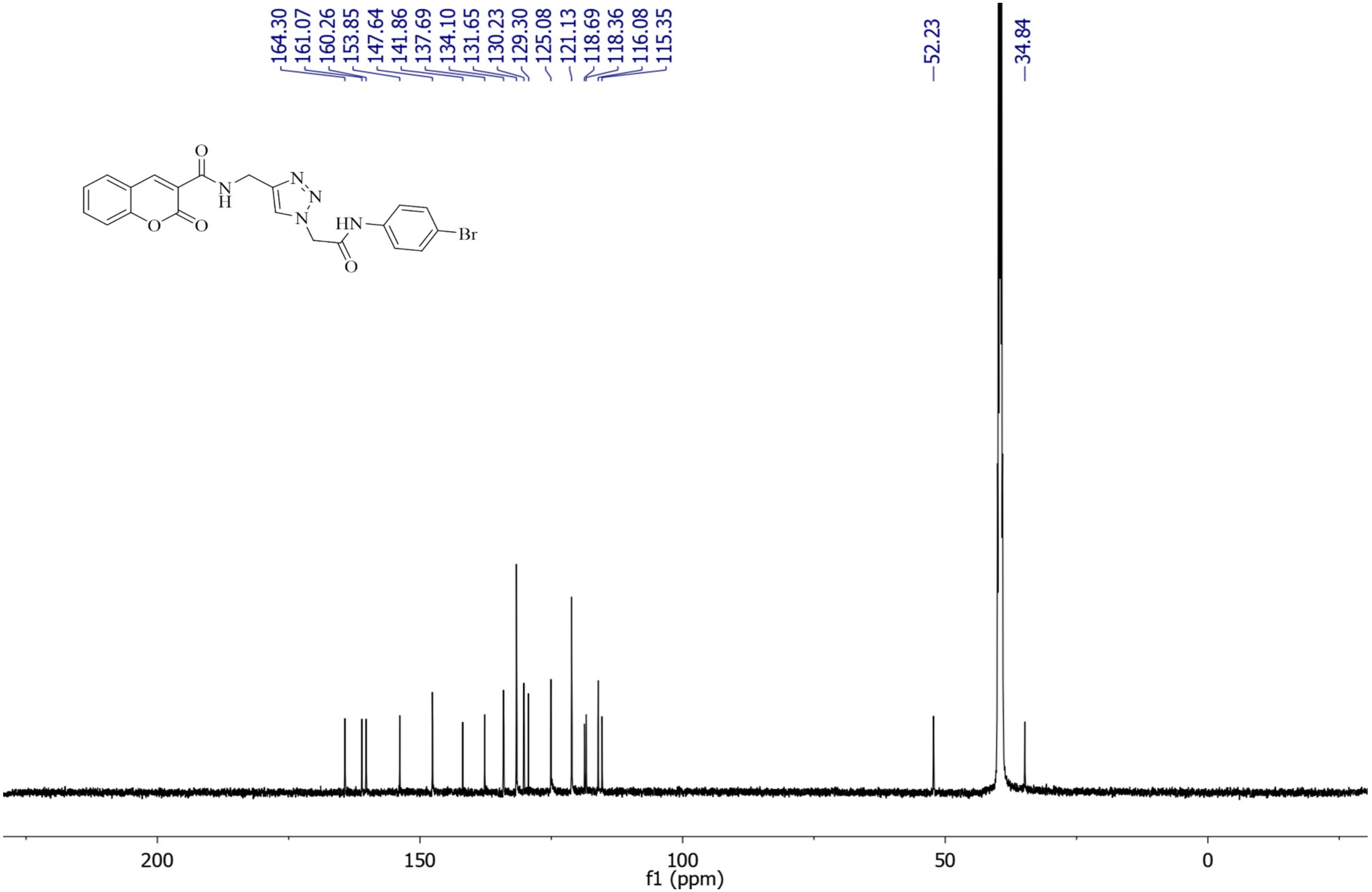

carboxamide (**12n**)

^{1}H NMR spectrum of 2-oxo-N-((1-(2-oxo-2-((4-(trifluoromethyl)phenyl)amino)ethyl)-1H-1,2,3-triazol-4-yl)methyl)-2H-chromene-3-carboxamide (**12o**)

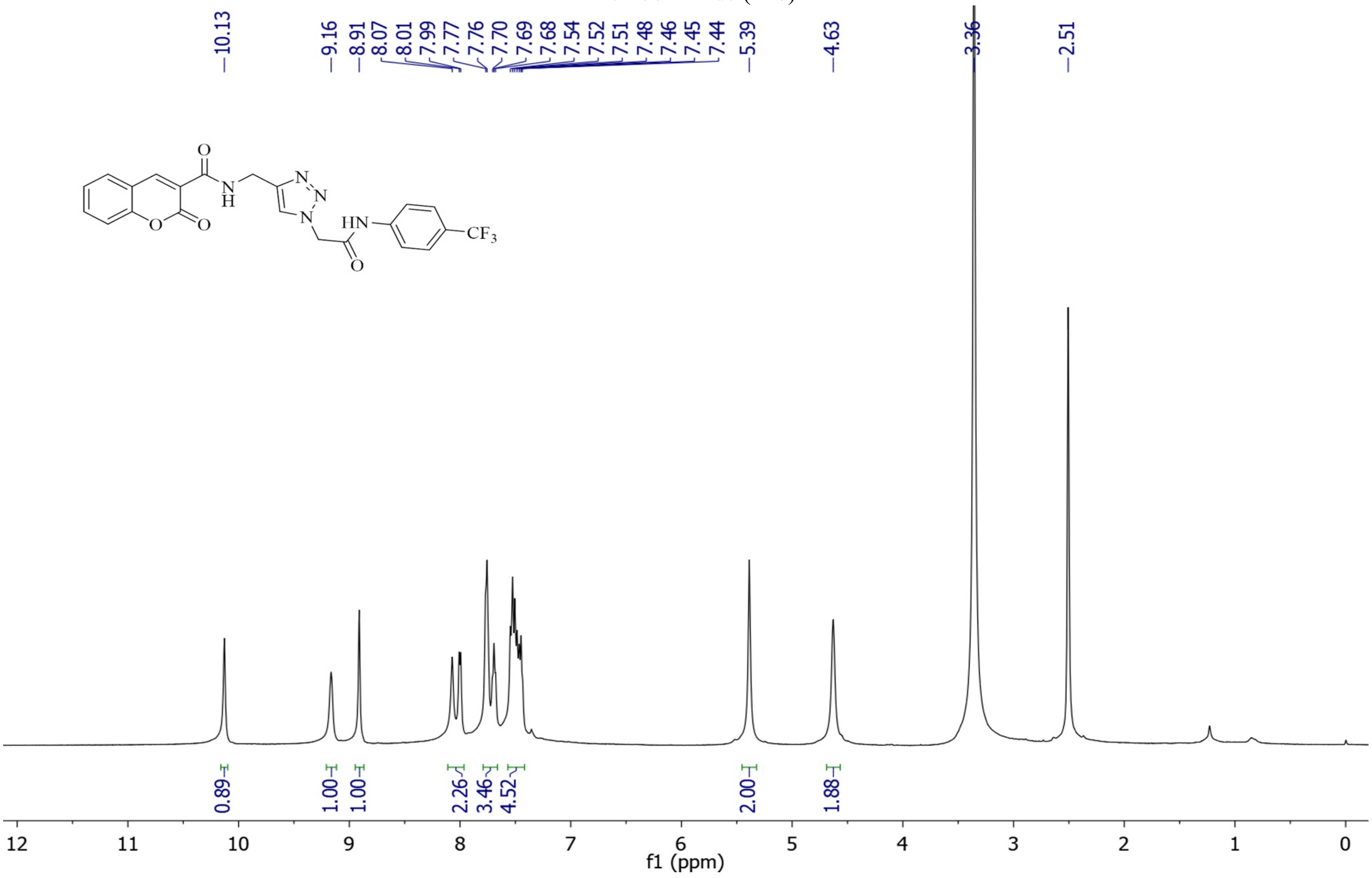

^{13}C NMR spectrum of 2-oxo-N-((1-(2-oxo-2-((4-(trifluoromethyl)phenyl)amino)ethyl)-1H-1,2,3-triazol-4-yl)methyl)-2H-chromene-3-carboxamide (**12o**)

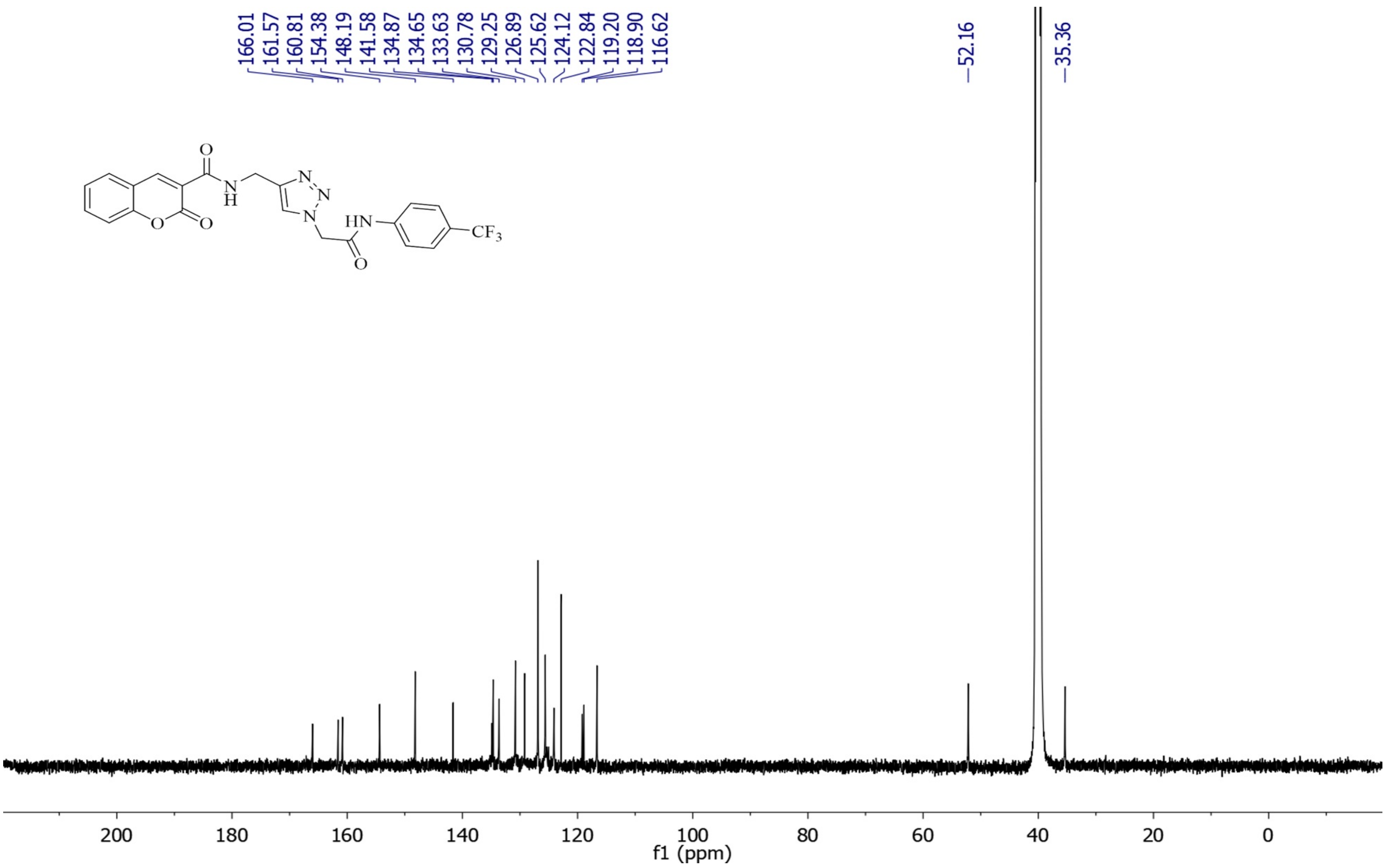

^{1}H NMR spectrum of N-((1-(2-((2-cyanophenyl)amino)-2-oxoethyl)-1H-1,2,3-triazol-4-yl)methyl)-2-oxo-2H-chromene-3-carboxamide (**12p**)

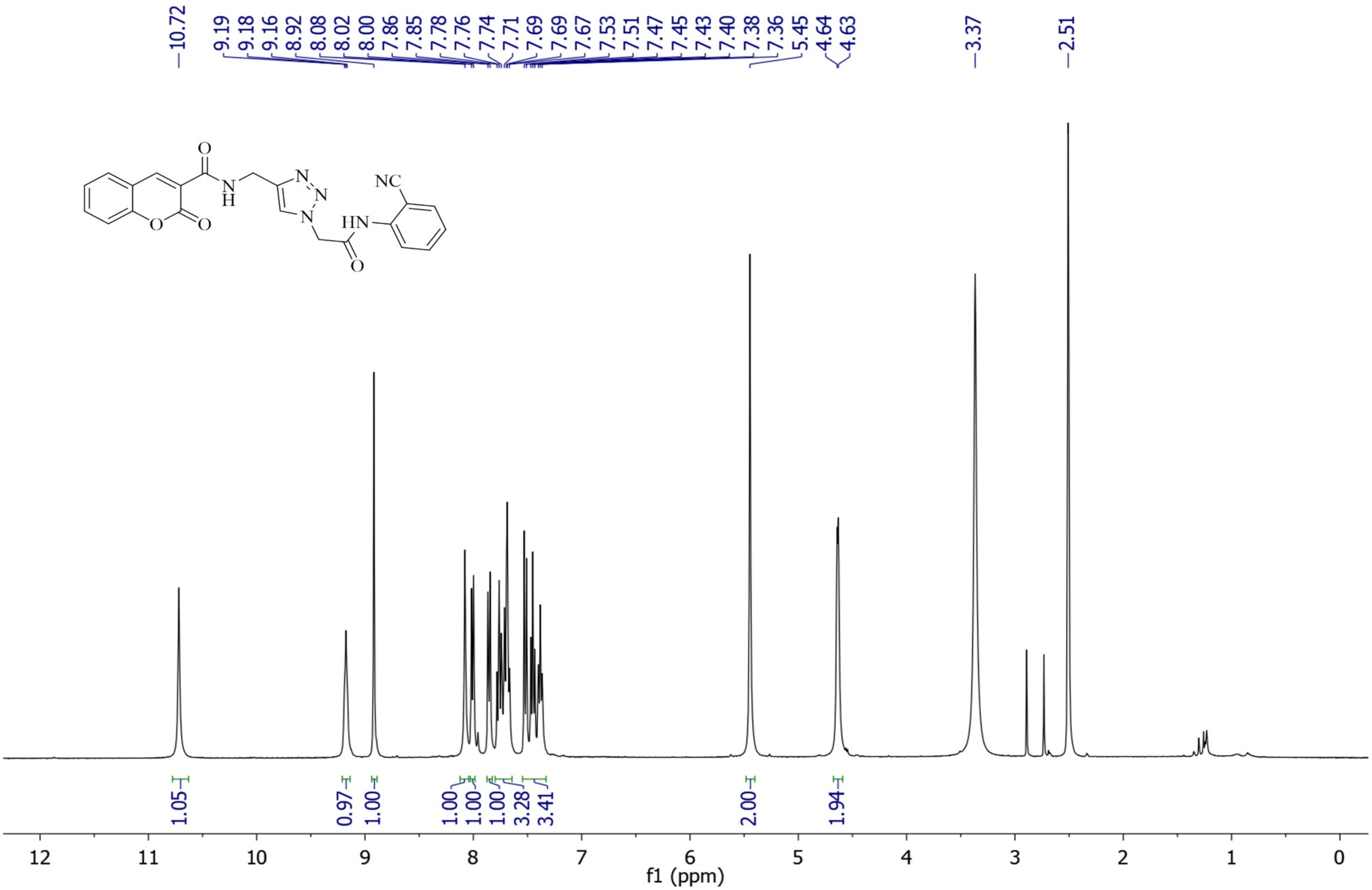

^{13}C NMR spectrum of N-((1-(2-((2-cyanophenyl)amino)-2-oxoethyl)-1H-1,2,3-triazol-4-yl)methyl)-2-oxo-2H-chromene-3-

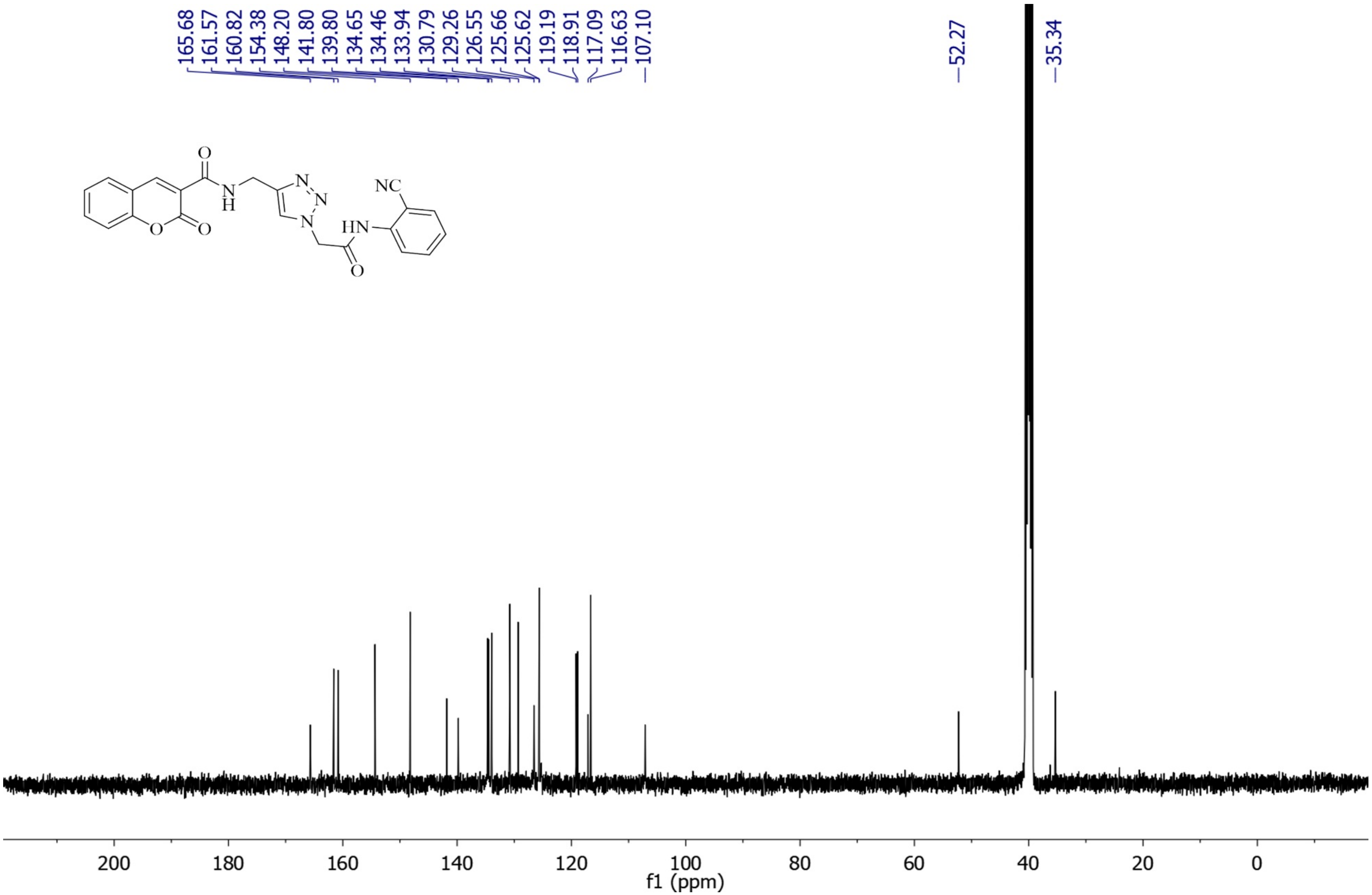

carboxamide (**12p**)

^{1}H NMR spectrum of N-((1-(2-((3-cyanophenyl)amino)-2-oxoethyl)-1H-1,2,3-triazol-4-yl)methyl)-2-oxo-2H-chromene-3-

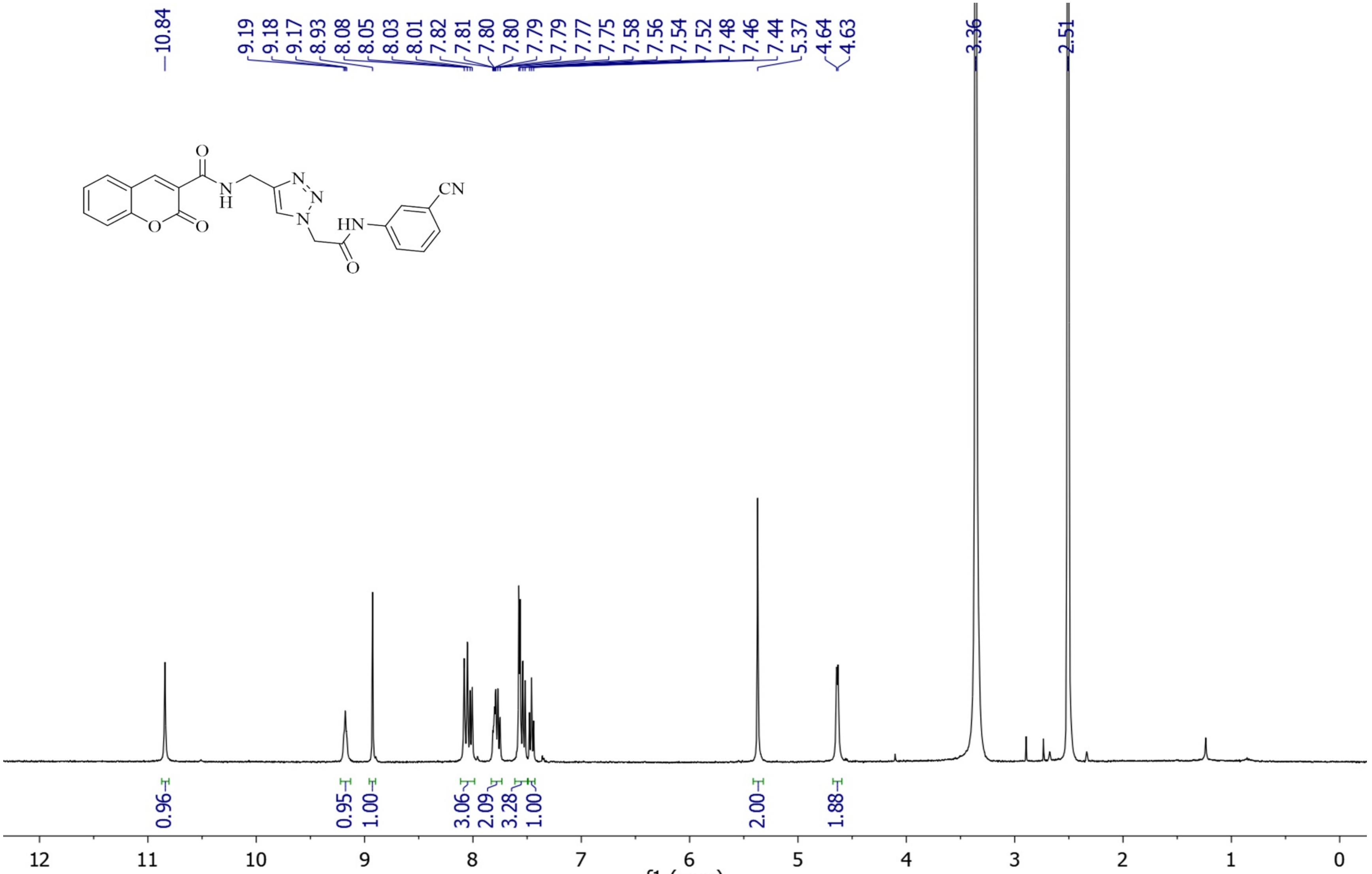

carboxamide (**12q**)

^{13}C NMR spectrum of N-((1-(2-((3-cyanophenyl)amino)-2-oxoethyl)-1H-1,2,3-triazol-4-yl)methyl)-2-oxo-2H-chromene-3-

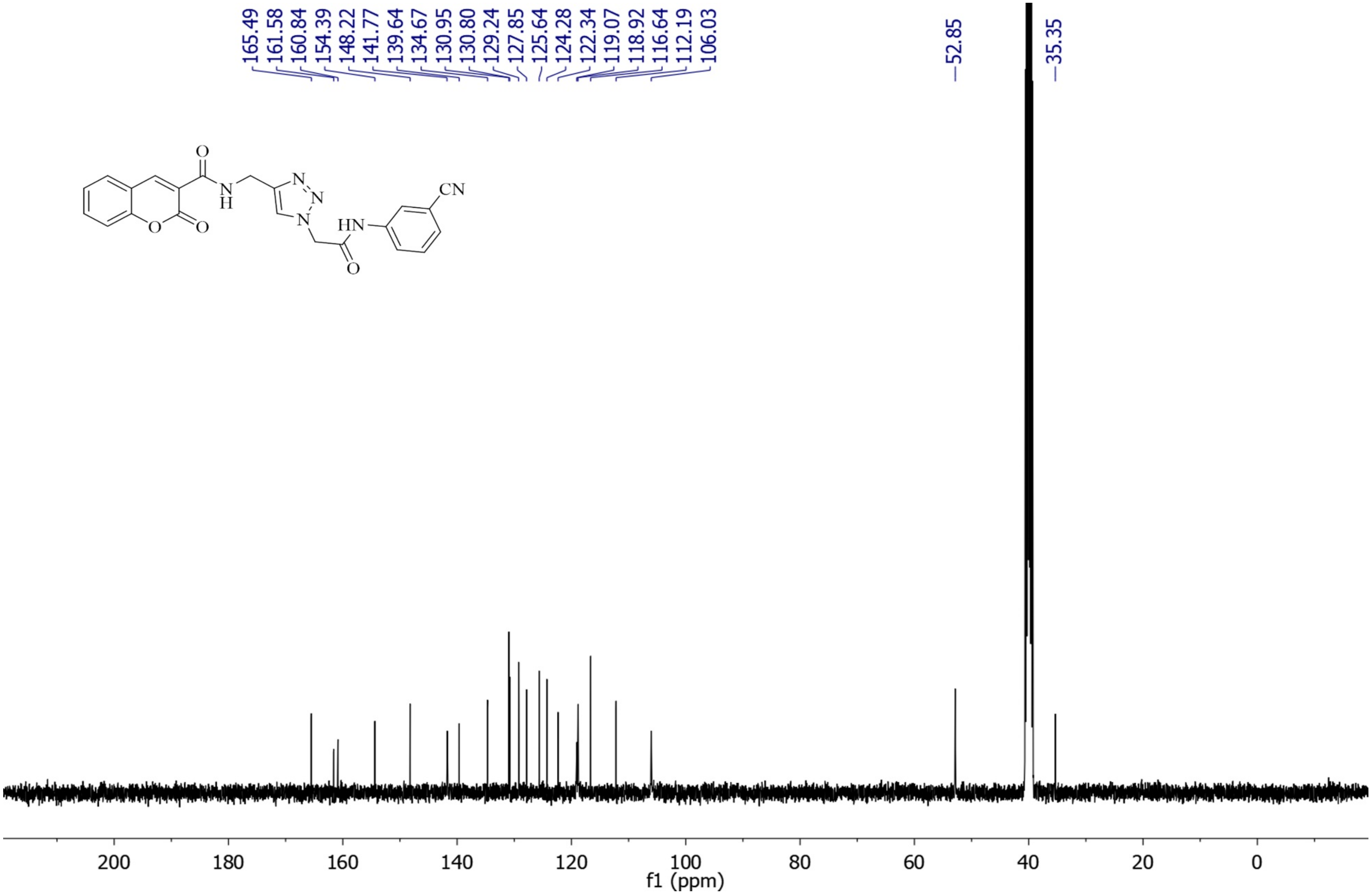

carboxamide (**12q**)

^{1}H NMR spectrum of N-((1-(2-((4-cyanophenyl)amino)-2-oxoethyl)-1H-1,2,3-triazol-4-yl)methyl)-2-oxo-2H-chromene-3-

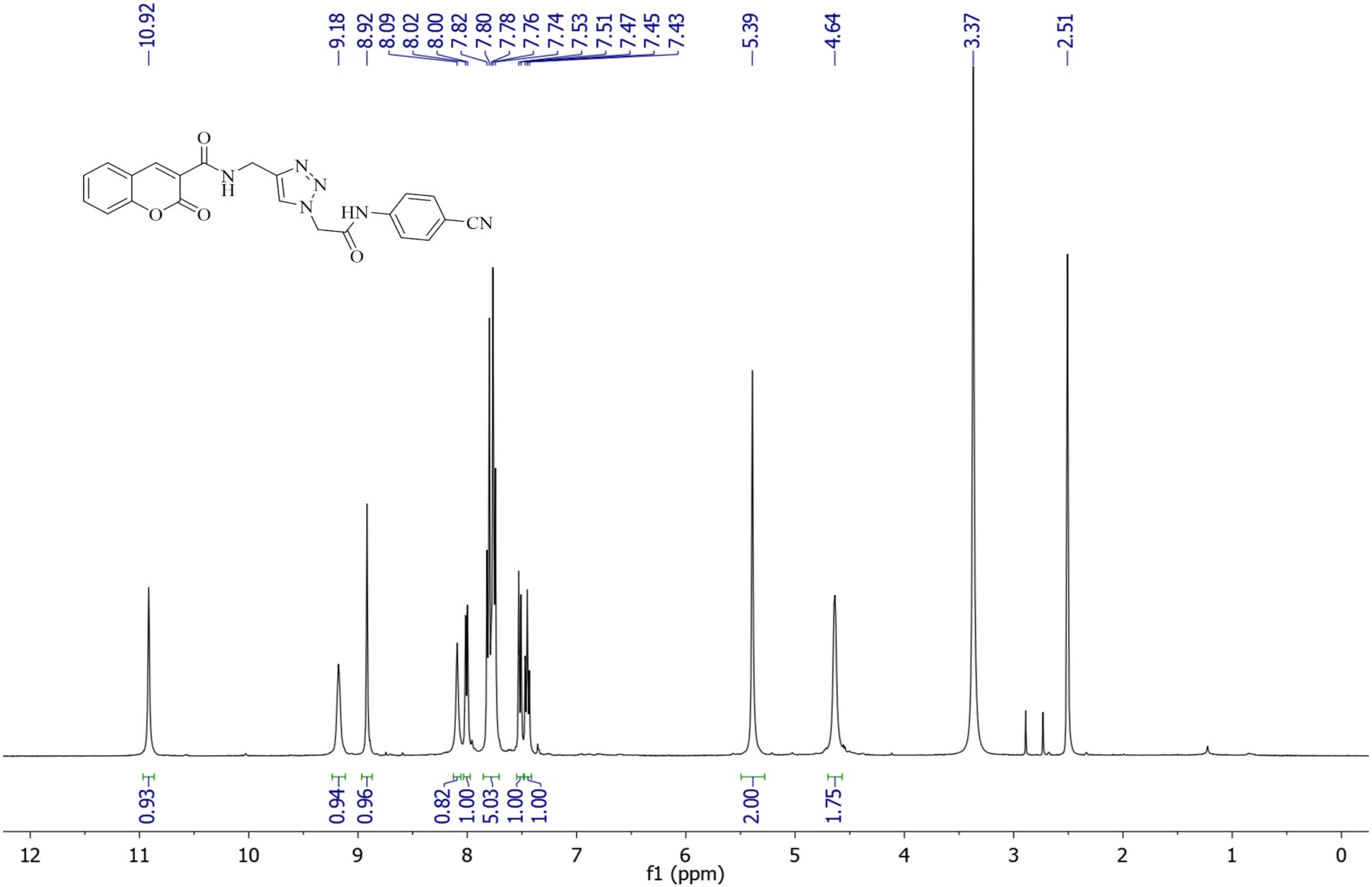

carboxamide (**12r**)

^{13}C NMR spectrum of N-((1-(2-((4-cyanophenyl)amino)-2-oxoethyl)-1H-1,2,3-triazol-4-yl)methyl)-2-oxo-2H-chromene-3-

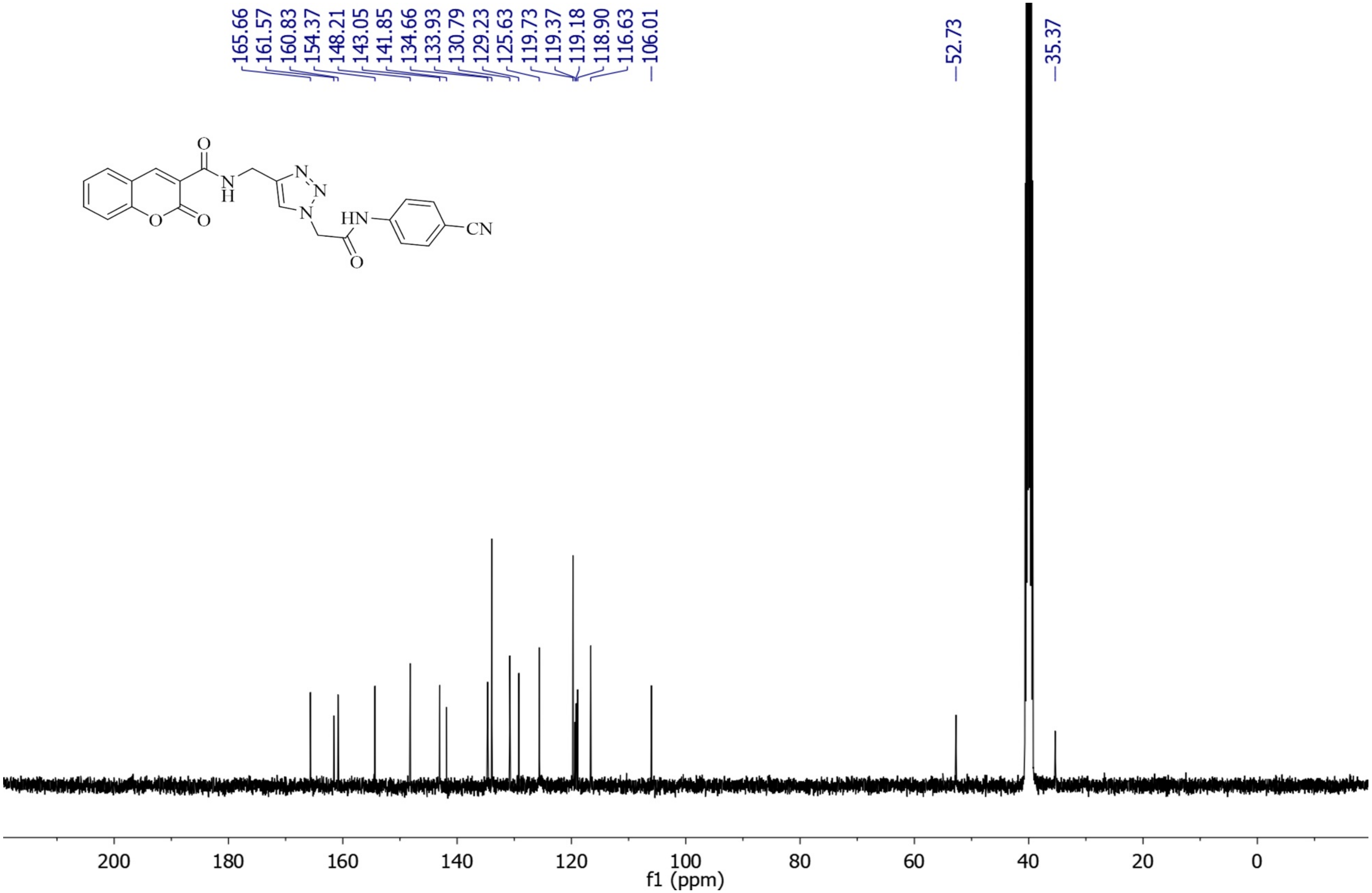

carboxamide (**12r**)

^{1}H NMR spectrum of N-((1-benzyl-1H-1,2,3-triazol-4-yl)methyl)-2-oxo-2H-chromene-3-carboxamide (**13**)

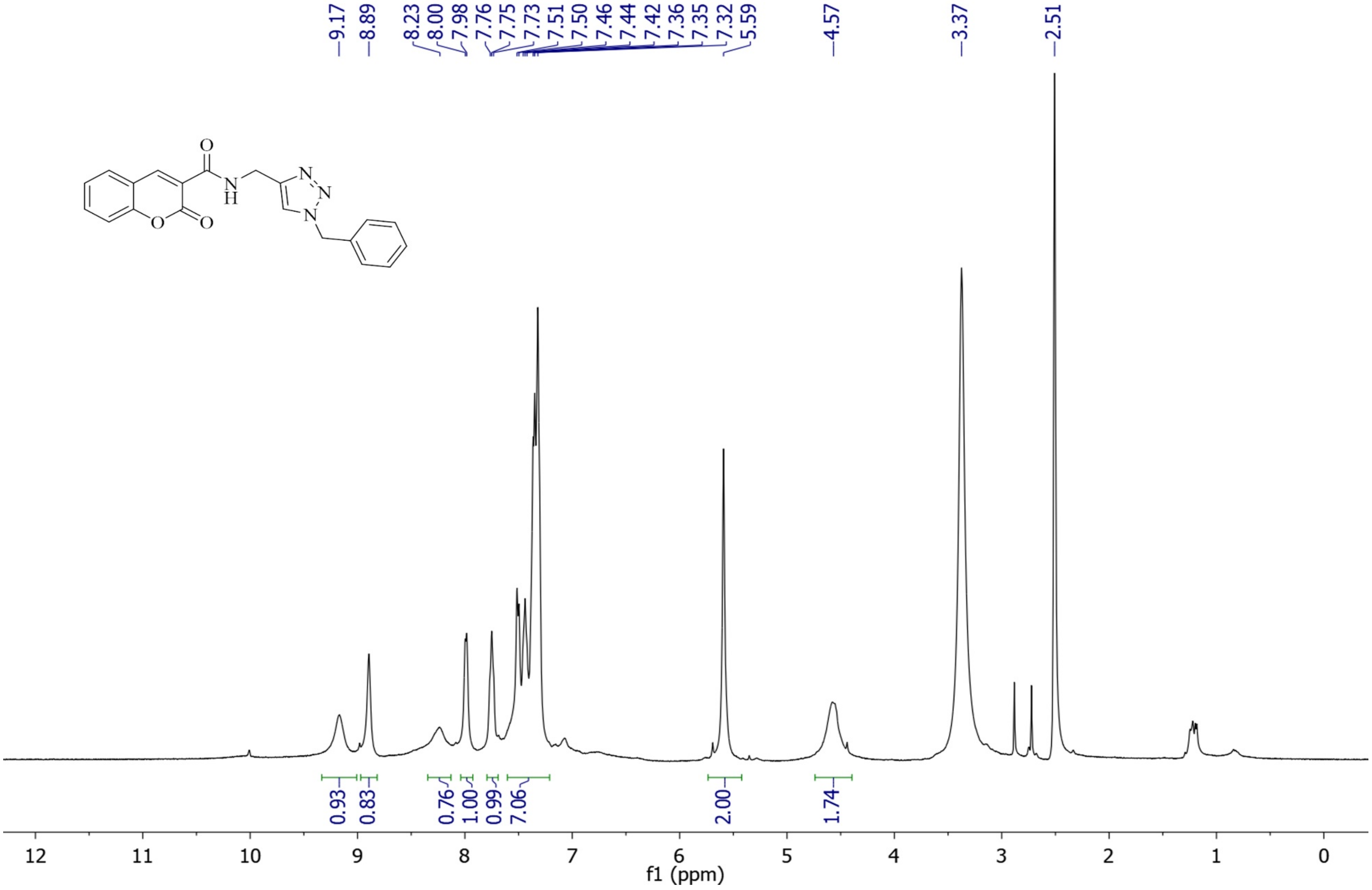

^{13}C NMR spectrum of N-((1-benzyl-1H-1,2,3-triazol-4-yl)methyl)-2-oxo-2H-chromene-3-carboxamide (**13**)

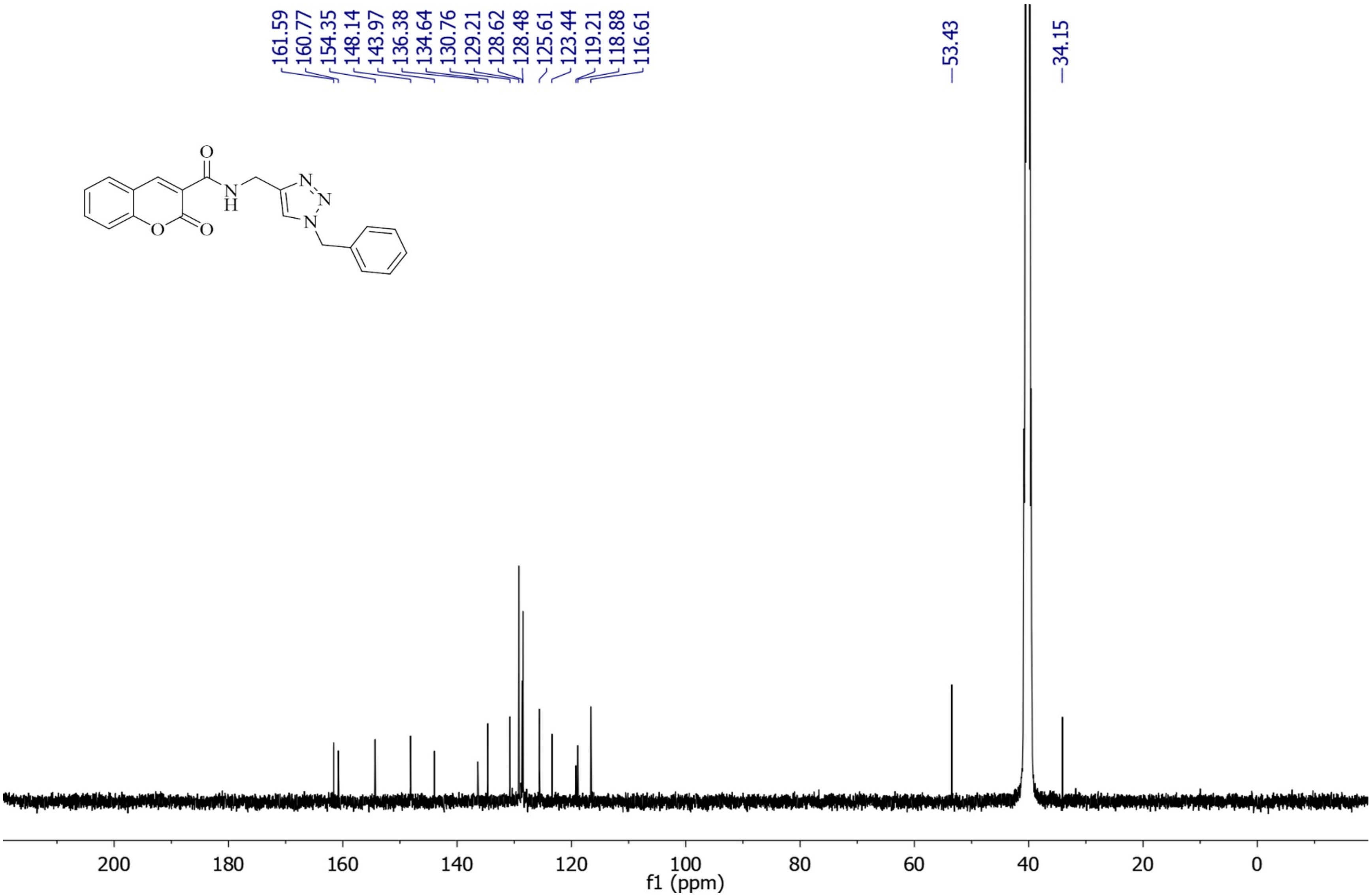

^{1}H NMR spectrum of N-((1-(4-chlorobenzyl)-1H-1,2,3-triazol-4-yl)methyl)-2-oxo-2H-chromene-3-carboxamide (**14**)

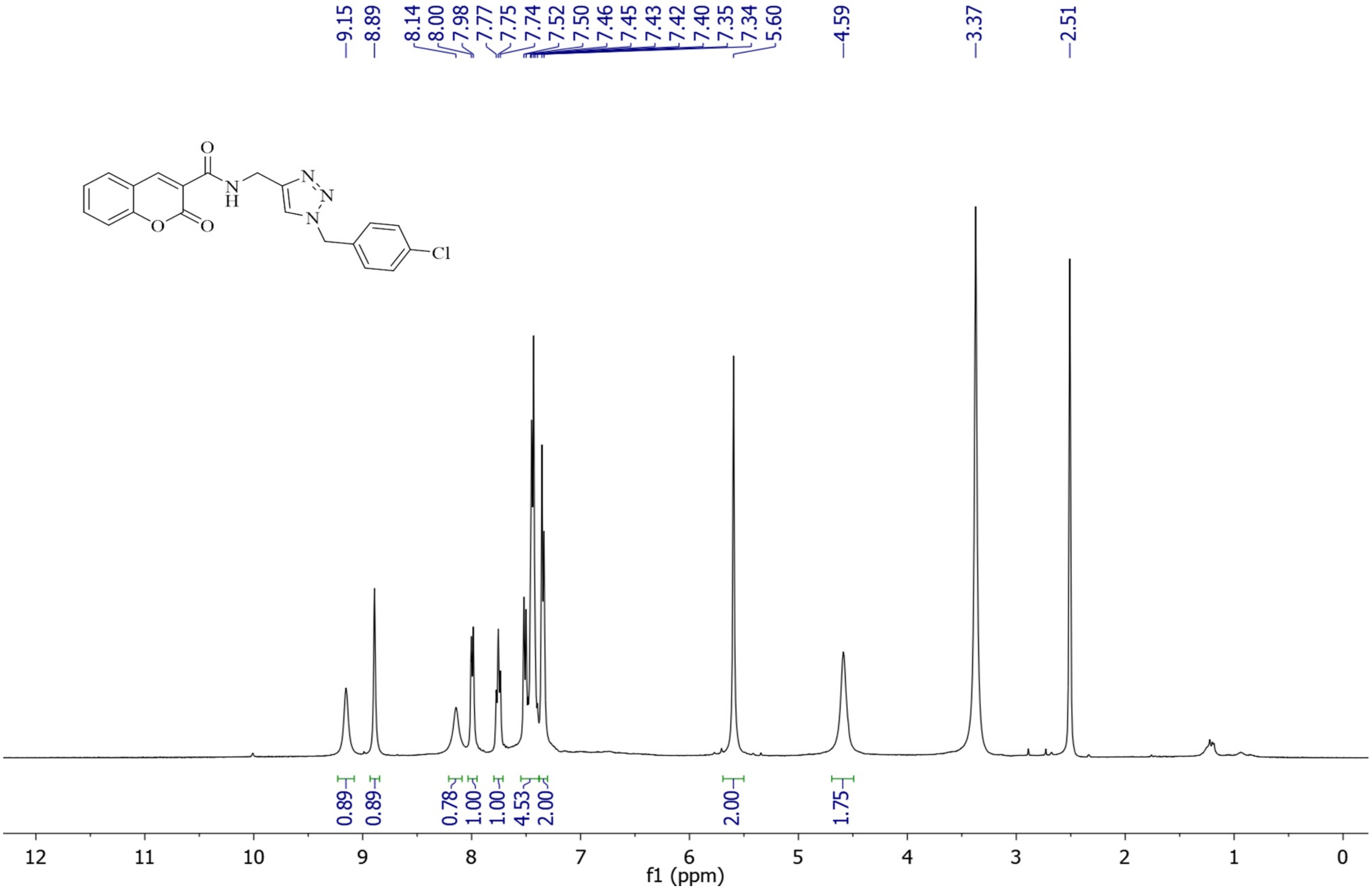

^{13}C NMR spectrum of N-((1-(4-chlorobenzyl)-1H-1,2,3-triazol-4-yl)methyl)-2-oxo-2H-chromene-3-carboxamide (**14**)

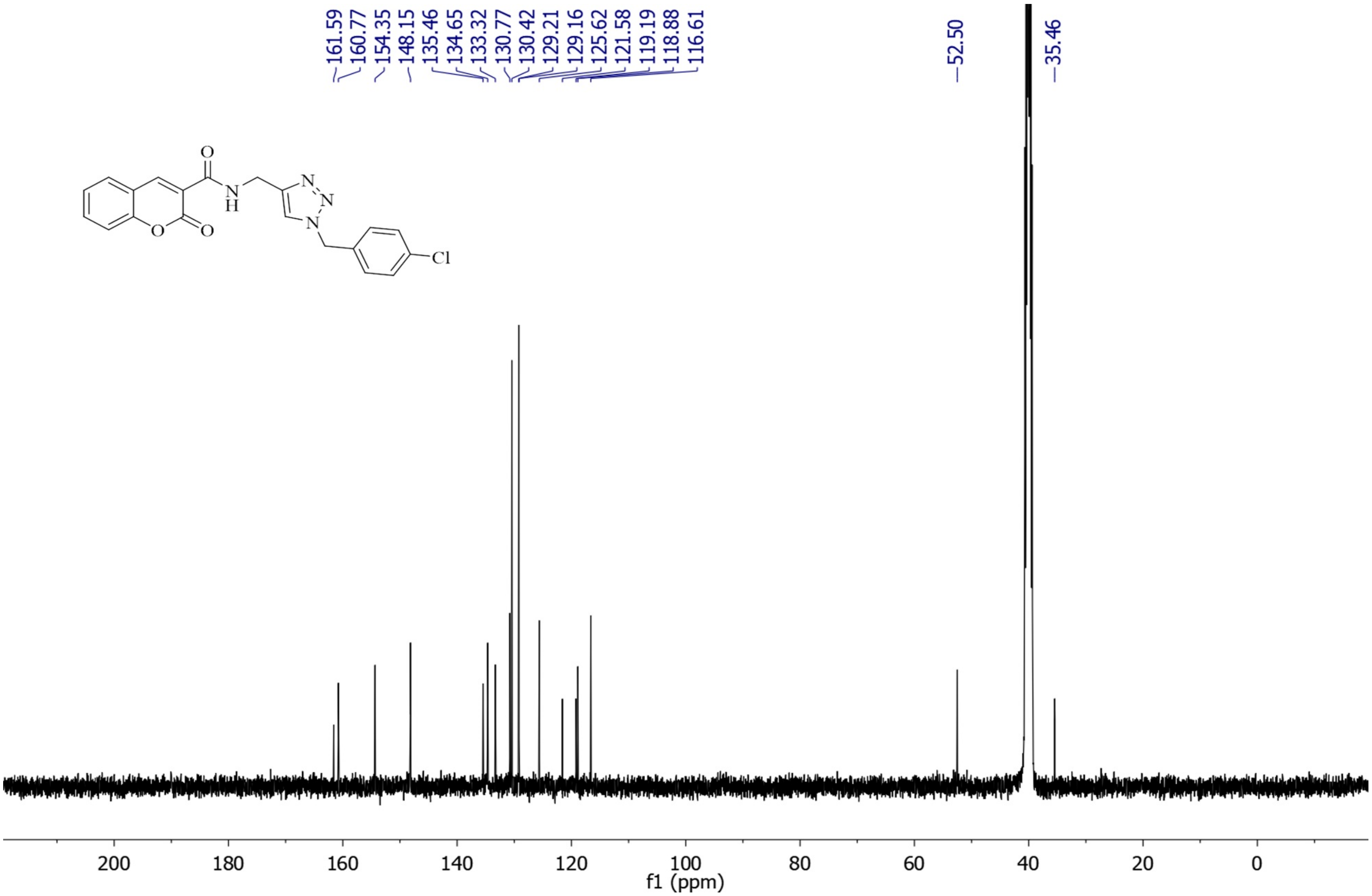

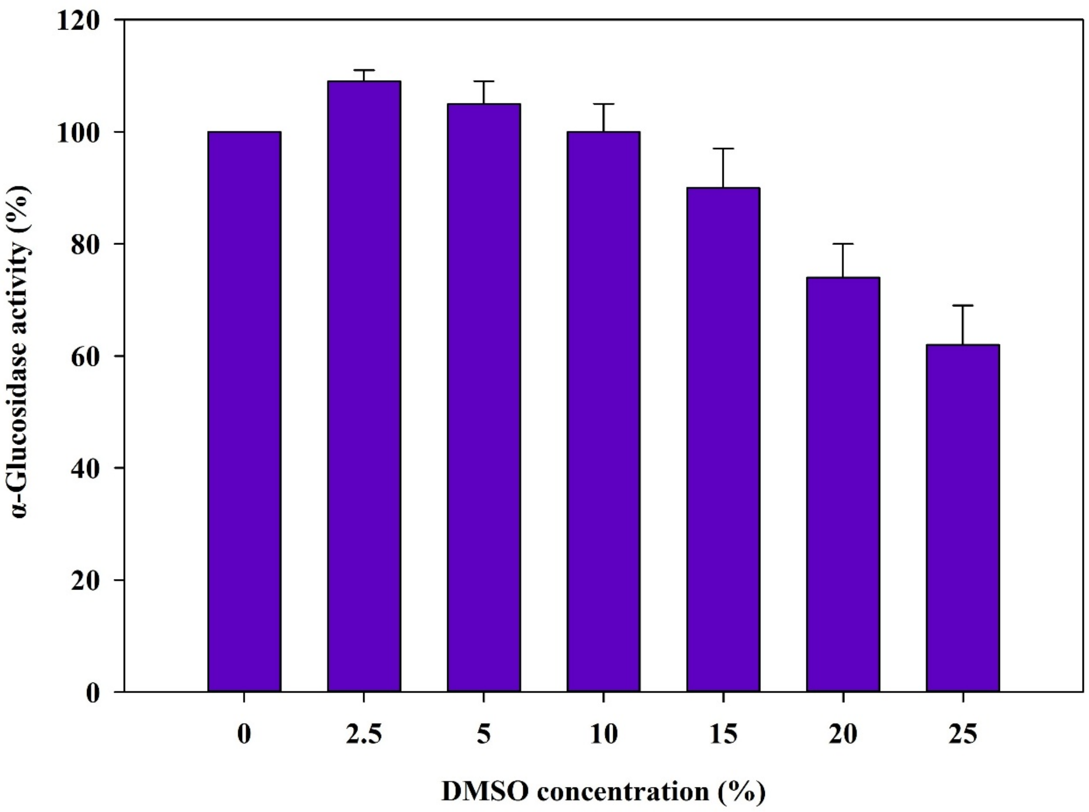

Figure S-1. Enzyme activity was measured in the presence of increasing DMSO concentrations (0–25%) to determine the solvent level that does not interfere with assay performance.

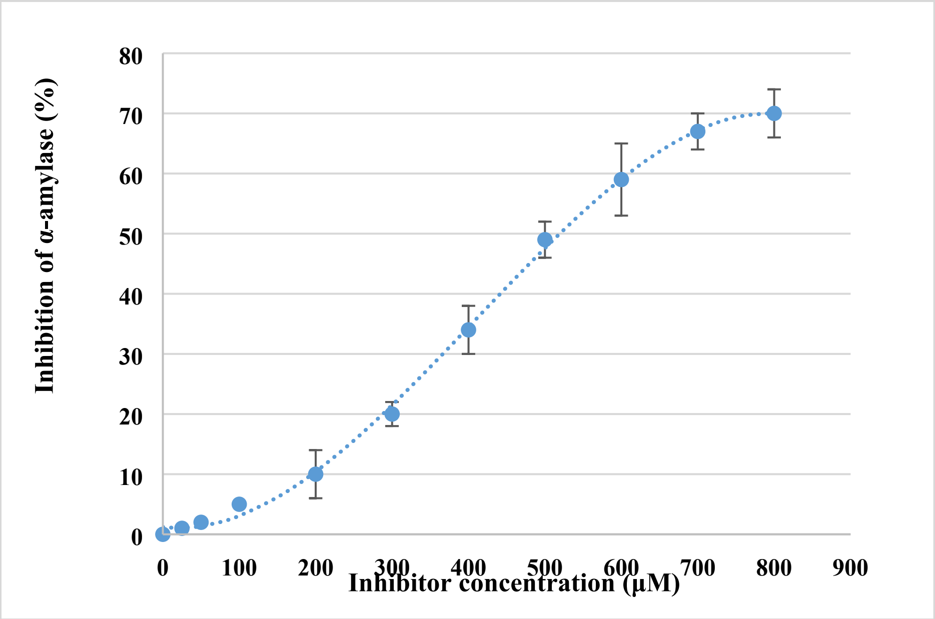

Figure S-2. Dose–response curves for α-amylase inhibition by **12q**.

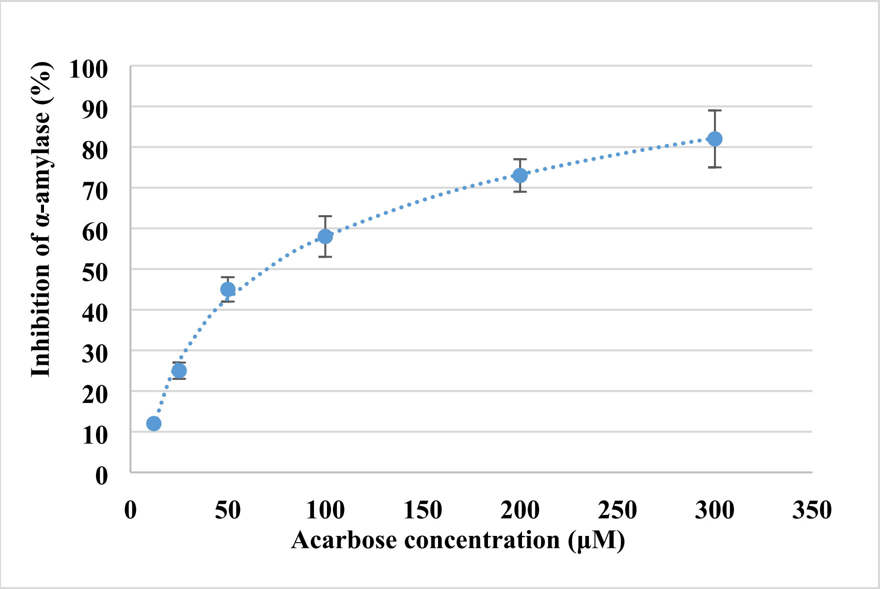

Figure S-3. Dose–response curves for α-amylase inhibition by acarbose

Table S1. CD spectral data of the solvent blank (water with DMSO) used for baseline correction.

Wavelength (nm)	CD (mdeg)
260	1.1963
259.5	1.19802
259	1.1982
258.5	1.20673
258	1.16295
257.5	1.09961
257	1.08518
256.5	1.10869
256	1.10527
255.5	1.0702
255	1.04418
254.5	0.993123
254	0.9929
253.5	1.01156
253	0.972287
252.5	0.95024
252	0.920639
251.5	0.936825
251	0.926388
250.5	0.97096
250	1.02373
249.5	1.07491
249	1.04214
248.5	1.05364
248	1.08169
247.5	1.13542
247	1.13507
246.5	1.11929
246	1.13051
245.5	1.11745

Wavelength (nm)	CD (mdeg)
245	1.11939
244.5	1.07691
244	1.00911
243.5	0.878117
243	0.784745
242.5	0.779528
242	0.72541
241.5	0.597201
241	0.488612
240.5	0.429016
240	0.30033
239.5	0.247962
239	0.205678
238.5	0.180211
238	0.091691
237.5	-0.01252
237	-0.05811
236.5	-0.14555
236	-0.24777
235.5	-0.31106
235	-0.30598
234.5	-0.49671
234	-0.62714
233.5	-0.64007
233	-0.76206
232.5	-0.91259
232	-1.08762
231.5	-1.17577
231	-1.25956
230.5	-1.32298
230	-1.31352

Wavelength (nm)	CD (mdeg)
229.5	-1.35574
229	-1.38129
228.5	-1.5988
228	-1.50233
227.5	-1.33652
227	-1.42413
226.5	-1.33822
226	-1.06087
225.5	-1.03964
225	-0.88397
224.5	-0.77679
224	-0.82783
223.5	-0.56831
223	-0.15279
222.5	0.25508
222	0.679651
221.5	1.04706
221	1.23776
220.5	1.25732
220	1.9003
219.5	2.06704
219	2.12142
218.5	2.54505
218	2.8199
217.5	3.25677
217	3.52899
216.5	3.58734
216	3.30788
215.5	2.94046
215	3.05161
214.5	3.36373

**Wavelength (nm)**	**CD (mdeg)**
214	2.65477
213.5	2.06609
213	1.45921
212.5	1.63032
212	1.71812
211.5	1.92864
211	1.7839
210.5	1.60022
210	1.43445
209.5	1.14506
209	2.30544
208.5	2.74118
208	3.26309
207.5	4.54237
207	5.68096
206.5	6.92148
206	7.27819
205.5	7.82798
205	7.69413
204.5	7.35949
204	8.6152
203.5	9.49045
203	10.6722
202.5	10.9402
202	11.9375
201.5	11.9339
201	12.207
200.5	12.6552
200	12.4207
199.5	12.3234
199	13.2958

Wavelength (nm)	CD (mdeg)
198.5	14.1087
198	16.1749
197.5	17.6393
197	19.0784
196.5	18.8805
196	19.5819
195.5	14.5158
195	15.0761
194.5	14.7716
194	9.67434
193.5	7.61862
193	10.5225
192.5	15.5618
192	15.1041
191.5	15.2314
191	8.63095
190.5	1.54781
190	-6.44348

Table S2. Raw CD spectral data of α-glucosidase in the presence of inhibitor in water containing DMSO.

Wavelength (nm)	CD (mdeg)
260	1.47841
259.5	1.49801
259	1.4527
258.5	1.46355
258	1.42172
257.5	1.42919
257	1.34776
256.5	1.36061

**Wavelength (nm)**	**CD (mdeg)**
256	1.30789
255.5	1.29593
255	1.2904
254.5	1.27279
254	1.24084
253.5	1.24212
253	1.21946
252.5	1.18912
252	1.183
251.5	1.16528
251	1.12078
250.5	1.05767
250	0.984872
249.5	0.956555
249	0.923476
248.5	0.932481
248	0.862227
247.5	0.784867
247	0.779057
246.5	0.75924
246	0.731561
245.5	0.680909
245	0.639178
244.5	0.586439
244	0.620979
243.5	0.668407
243	0.685207
242.5	0.692979
242	0.648941
241.5	0.578315

**Wavelength (nm)**	**CD (mdeg)**
241	0.608456
240.5	0.552338
240	0.498001
239.5	0.444381
239	0.440189
238.5	0.382521
238	0.343761
237.5	0.246739
237	0.081972
236.5	-0.06679
236	-0.18944
235.5	-0.29811
235	-0.45043
234.5	-0.58531
234	-0.68964
233.5	-0.82703
233	-0.96908
232.5	-1.10571
232	-1.29221
231.5	-1.3456
231	-1.46331
230.5	-1.56886
230	-1.69475
229.5	-1.7805
229	-1.92364
228.5	-2.02268
228	-2.11756
227.5	-2.19798
227	-2.33187
226.5	-2.39658

Wavelength (nm)	CD (mdeg)
226	-2.53175
225.5	-2.56103
225	-2.63893
224.5	-2.85936
224	-2.88697
223.5	-2.87772
223	-2.86211
222.5	-2.95564
222	-2.97639
221.5	-3.0412
221	-3.13459
220.5	-3.13486
220	-3.1969
219.5	-3.17366
219	-3.2287
218.5	-3.34293
218	-3.25812
217.5	-3.27514
217	-3.42239
216.5	-3.54212
216	-3.61422
215.5	-3.60352
215	-3.60034
214.5	-3.53252
214	-3.55986
213.5	-3.55932
213	-3.68448
212.5	-3.76709
212	-3.6864
211.5	-3.6934

Wavelength (nm)	CD (mdeg)
211	-3.68824
210.5	-3.63003
210	-3.6286
209.5	-3.55444
209	-3.53782
208.5	-3.50163
208	-3.4257
207.5	-3.36125
207	-3.34708
206.5	-3.16491
206	-2.97628
205.5	-2.81102
205	-2.66173
204.5	-2.48741
204	-2.24661
203.5	-1.93502
203	-1.62853
202.5	-1.26665
202	-0.9005
201.5	-0.46838
201	-0.0534
200.5	0.418504
200	0.857472
199.5	1.39156
199	1.82096
198.5	2.30547
198	2.7436
197.5	3.21379
197	3.67389
196.5	4.10429

Wavelength (nm)	CD (mdeg)
196	4.51021
195.5	4.73912
195	5.01079
194.5	5.28736
194	5.48059
193.5	5.7101
193	5.71539
192.5	5.78238
192	5.92154
191.5	5.96635
191	6.05968
190.5	5.9902
190	6.03496

Table S3. CD spectral data of the inhibitor solution in water containing DMSO in the absence of enzyme.

Wavelength (nm)	CD (mdeg)
260	1.1963
259.5	1.19802
259	1.1982
258.5	1.20673
258	1.16295
257.5	1.09961
257	1.08518
256.5	1.10869
256	1.10527
255.5	1.0702
255	1.04418
254.5	0.993123
254	0.9929

**Wavelength (nm)**	**CD (mdeg)**
253.5	1.01156
253	0.972287
252.5	0.95024
252	0.920639
251.5	0.936825
251	0.926388
250.5	0.97096
250	1.02373
249.5	1.07491
249	1.04214
248.5	1.05364
248	1.08169
247.5	1.13542
247	1.13507
246.5	1.11929
246	1.13051
245.5	1.11745
245	1.11939
244.5	1.07691
244	1.00911
243.5	0.878117
243	0.784745
242.5	0.779528
242	0.72541
241.5	0.597201
241	0.488612
240.5	0.429016
240	0.30033
239.5	0.247962
239	0.205678
238.5	0.180211

**Wavelength (nm)**	**CD (mdeg)**
238	0.091691
237.5	-0.01252
237	-0.05811
236.5	-0.14555
236	-0.24777
235.5	-0.31106
235	-0.30598
234.5	-0.49671
234	-0.62714
233.5	-0.64007
233	-0.76206
232.5	-0.91259
232	-1.08762
231.5	-1.17577
231	-1.25956
230.5	-1.32298
230	-1.31352
229.5	-1.35574
229	-1.38129
228.5	-1.5988
228	-1.50233
227.5	-1.33652
227	-1.42413
226.5	-1.33822
226	-1.06087
225.5	-1.03964
225	-0.88397
224.5	-0.77679
224	-0.82783
223.5	-0.56831
223	-0.15279

**Wavelength (nm)**	**CD (mdeg)**
222.5	0.25508
222	0.679651
221.5	1.04706
221	1.23776
220.5	1.25732
220	1.9003
219.5	2.06704
219	2.12142
218.5	2.54505
218	2.8199
217.5	3.25677
217	3.52899
216.5	3.58734
216	3.30788
215.5	2.94046
215	3.05161
214.5	3.36373
214	2.65477
213.5	2.06609
213	1.45921
212.5	1.63032
212	1.71812
211.5	1.92864
211	1.7839
210.5	1.60022
210	1.43445
209.5	1.14506
209	2.30544
208.5	2.74118
208	3.26309
207.5	4.54237

Wavelength (nm)	CD (mdeg)
207	5.68096
206.5	6.92148
206	7.27819
205.5	7.82798
205	7.69413
204.5	7.35949
204	8.6152
203.5	9.49045
203	10.6722
202.5	10.9402
202	11.9375
201.5	11.9339
201	12.207
200.5	12.6552
200	12.4207
199.5	12.3234
199	13.2958
198.5	14.1087
198	16.1749
197.5	17.6393
197	19.0784
196.5	18.8805
196	19.5819
195.5	14.5158
195	15.0761
194.5	14.7716
194	9.67434
193.5	7.61862
193	10.5225
192.5	15.5618
192	15.1041

Wavelength (nm)	CD (mdeg)
191.5	15.2314
191	8.63095
190.5	1.54781
190	-6.44348

Table S4. CD spectral data of the inhibitor solution in water containing DMSO and enzyme.

Wavelength (nm)	CD (mdeg)
260	2.2767514
259.5	2.3069354
259	2.237158
258.5	2.253867
258	2.1894488
257.5	2.2009526
257	2.0755504
256.5	2.0953394
256	2.0141506
255.5	1.9957322
255	1.987216
254.5	1.9600966
254	1.9108936
253.5	1.9128648
253	1.8779684
252.5	1.8312448
252	1.82182
251.5	1.7945312
251	1.7260012
250.5	1.6288118
250	1.51670288
249.5	1.4730947
249	1.42215304

**Wavelength (nm)**	**CD (mdeg)**
248.5	1.43602074
248	1.32782958
247.5	1.20869518
247	1.19974778
246.5	1.1692296
246	1.12660394
245.5	1.04859986
245	0.98433412
244.5	0.90311606
244	0.95630766
243.5	1.02934678
243	1.05521878
242.5	1.06718766
242	0.99936914
241.5	0.8906051
241	0.93702224
240.5	0.85060052
240	0.76692154
239.5	0.68434674
239	0.67789106
238.5	0.58908234
238	0.52939194
237.5	0.37997806
237	0.12623688
236.5	-0.1028566
236	-0.2917376
235.5	-0.4590894
235	-0.6936622
234.5	-0.9013774
234	-1.0620456
233.5	-1.2736262

**Wavelength (nm)**	**CD (mdeg)**
233	-1.4923832
232.5	-1.7027934
232	-1.9900034
231.5	-2.072224
231	-2.2534974
230.5	-2.4160444
230	-2.609915
229.5	-2.74197
229	-2.9624056
228.5	-3.1149272
228	-3.2610424
227.5	-3.3848892
227	-3.5910798
226.5	-3.6907332
226	-3.898895
225.5	-3.9439862
225	-4.0639522
224.5	-4.4034144
224	-4.4459338
223.5	-4.4316888
223	-4.4076494
222.5	-4.5516856
222	-4.5836406
221.5	-4.683448
221	-4.8272686
220.5	-4.8276844
220	-4.923226
219.5	-4.8874364
219	-4.972198
218.5	-5.1481122
218	-5.0175048

Wavelength (nm)	CD (mdeg)
217.5	-5.0437156
217	-5.2704806
216.5	-5.4548648
216	-5.5658988
215.5	-5.5494208
215	-5.5445236
214.5	-5.4400808
214	-5.4821844
213.5	-5.4813528
213	-5.6740992
212.5	-5.8013186
212	-5.677056
211.5	-5.687836
211	-5.6798896
210.5	-5.5902462
210	-5.588044
209.5	-5.4738376
209	-5.4482428
208.5	-5.3925102
208	-5.275578
207.5	-5.176325
207	-5.1545032
206.5	-4.8739614
206	-4.5834712
205.5	-4.3289708
205	-4.0990642
204.5	-3.8306114
204	-3.4597794
203.5	-2.9799308
203	-2.5079362
202.5	-1.950641

Wavelength (nm)	CD (mdeg)
202	-1.38677
201.5	-0.7213052
201	-0.082236
200.5	0.64449616
200	1.32050688
199.5	2.1430024
199	2.8042784
198.5	3.5504238
198	4.225144
197.5	4.9492366
197	5.6577906
196.5	6.3206066
196	6.9457234
195.5	7.2982448
195	7.7166166
194.5	8.1425344
194	8.4401086
193.5	8.793554
193	8.8017006
192.5	8.9048652
192	9.1191716
191.5	9.188179
191	9.3319072
190.5	9.224908
190	9.2938384

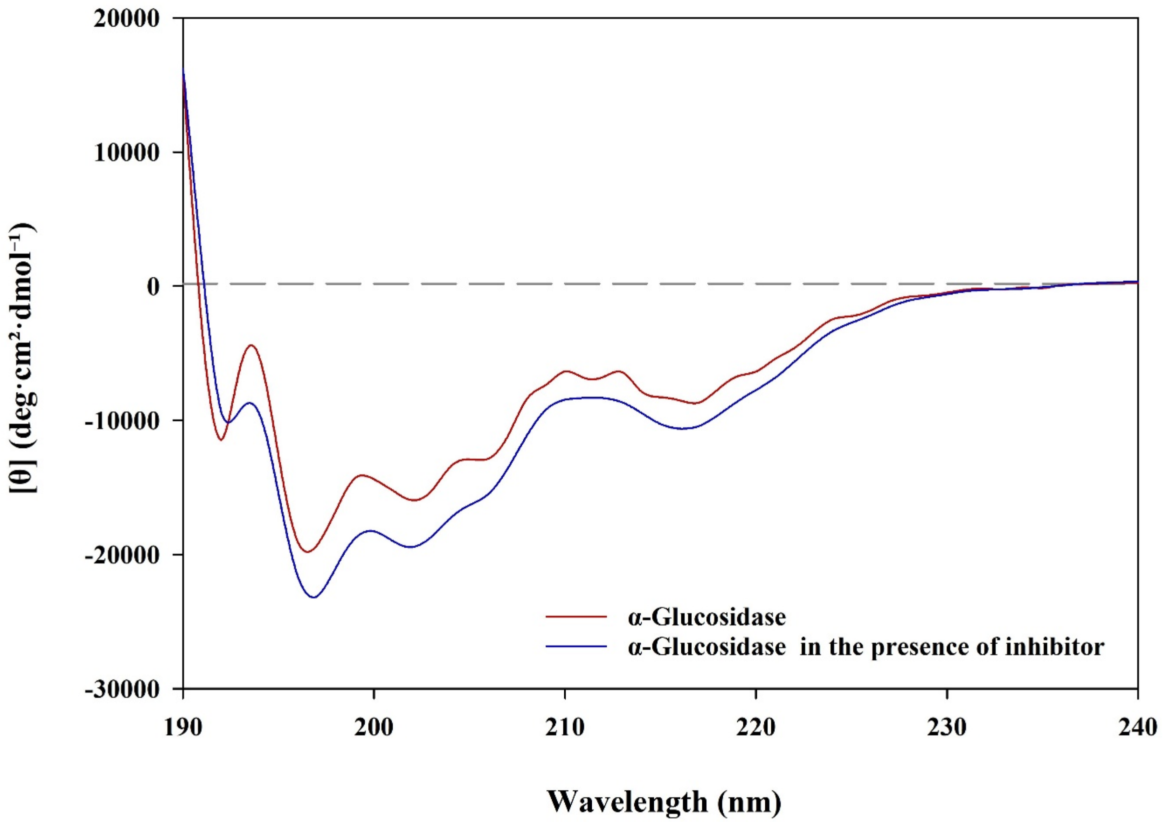

Figure S-4. Circular dichroism (CD) spectra of α-glucosidase in the absence and presence of inhibitor.

Nitrosamine risk assessment for each and every step of the reactions

Step (a)

Reaction: Knoevenagel condensation
Reagents: Diethyl malonate, piperidine, absolute EtOH, reflux, 4h

Although piperidine is a secondary amine and in the presence of a nitrosating agent, it can form N-Nitrosopiperidine (NPIP) [1]. ince the reaction took place under basic conditions (piperidine-catalyzed condensation), nitrosation is kinetically unfavorable. Furthermore, the subsequent work-up and the harsh saponification conditions in the next step ($NaOH_{(aq)}$, reflux) ensure that any potential volatile nitrosamine impurity is washed away.

Step (b)

Reaction: Alkaline ester hydrolysis
Reagents: NaOH (10% aq.), EtOH, reflux, 2h

No amine was used in this step. Industrial-grade NaOH can contain trace nitrite as an impurity, but pharmaceutical grade material (used in our laboratory) is very low in nitrites. Moreover, the use of 10% NaOH causes the destruction of any nitrites formed in the previous step.

Step (c)

Reaction: Amidation
Reagents: Propargylamine, TBTU, DIPEA, DMF, r.t., overnight

Propargylamine may form unstable N-nitrosopropargylamine, which rapidly deaminates. DIPEA (N,N-Diisopropylethylamine) is a tertiary amine, which is not directly nitrosatable. DMF, however, can degrade to dimethylamine (secondary amine), that is a precursor to the potent nitrosamine NDMA. Since the conditions are mild (r.t.) in this step, the basic reaction environment made nitrosation extremely unlikely. The aqueous work-up and purification processes remove any volatile amine impurities.

Step (d)

Reaction: Chloroacetylation
Reagents: Chloroacetyl chloride, TEA, acetone, r.t., overnight

Triethylamine (TEA) is a tertiary amine and therefore not directly nitrosatable. Nitrosation requires a secondary amine to form a stable N-nitrosamine. TEA can contain traces of diethylamine (a secondary amine) as a manufacturing impurity or degradation product. If present, diethylamine could theoretically form N-nitrosodiethylamine (NDEA). However, the basic reaction environment prevents nitrosation, and the subsequent water wash effectively remove any trace amine impurities.

Step (e)

Reaction: Nucleophilic azidation

Reagents: Sodium azide, DMF, r.t., overnight

No secondary or tertiary amine is deliberately added. DMF decomposition to dimethylamine is negligible at room temperature. Even if trace dimethylamine and nitrite collided, the neutral pH kinetically blocks nitrosation. Aqueous work-up removes any volatile amines.

Step (f)

Reaction: Click reaction
Reagents: $CuSO_4 \cdot 5H_2O$, sodium ascorbate, DMF, r.t., overnight

Same as the previous step, the absence of any added secondary amine and the negligible DMF degradation at room temperature prevent any direct nitrosamine formation pathway. Therefore, this step does not present a nitrosamine risk.

A thorough, stepwise evaluation of the synthetic route to the coumarin–triazole conjugates was performed, focusing on the potential formation of nitrosamines from secondary amines (piperidine, diethylamine from TEA, dimethylamine from DMF) in the presence of nitrosating agents. Considering the reaction conditions and purification processes, the likelihood of nitrosamine formation and its presence in the final coumarin–triazole conjugates is negligible. Moreover, the NMR graph of the final compounds don't show peaks related to such nitrosamines.

References:

1. De Mey, E., De Maere, H., Goemaere, O., Steen, L., Peeters, M.-C., Derdelinckx, G., Paelinck, H., Fraeye, I. (2014). Evaluation of N-nitrosopiperidine formation from biogenic amines during the production of dry fermented sausages. Food and bioprocess technology, 7: 1269-1280. https://doi.org/10.1007/s11947-013-1125-5.
